# Hi-Compass: a depth-aware deep learning framework for predicting cell-type-specific 3D genome organization from single-cell to spatial resolution

**DOI:** 10.1038/s41467-026-71877-z

**Published:** 2026-04-14

**Authors:** Yuan-Chen Sun, Wen-Jie Jiang, Kang-Wen Cai, Na-Na Wei, Fu-Ting Lai, Hao-Jie Wang, Rui-Xiang Gao, Ze-Yu Kuang, Jia-Lu Zhou, An Liu, Han-Wen Zhu, Yu-Juan Wang, Ming Xu, Hua-Jun Wu

**Affiliations:** 1 Key laboratory of Carcinogenesis and Translational Research (Ministry of Education/Beijing), Peking University Cancer Hospital & Institute, Beijing, China; 2 Department of Cardiology and Institute of Vascular Medicine, Peking University Third Hospital, State Key Laboratory of Vascular Homeostasis and Remodeling, NHC Key Laboratory of Cardiovascular Molecular Biology and Regulatory Peptides, Beijing Key Laboratory of Cardiovascular Receptors Research, Peking University, Beijing, China; 3 Key laboratory of Carcinogenesis and Translational Research (Ministry of Education/Beijing), Department of Lymphoma, Peking University Cancer Hospital & Institute, Beijing, China; 4 Department of Biomedical Informatics, School of Basic Medical Sciences, Peking University Health Science Center, Beijing, China; 5 Department of Gynecology and Obstetrics, Chinese PLA General Hospital, Beijing, China; 6 Research Unit of Medical Science Research Management/Basic and Clinical Research of Metabolic Cardiovascular Diseases, Chinese Academy of Medical Sciences, Beijing, China; 7 Beijing Advanced Center of Cellular Homeostasis and Aging-Related Diseases, Center for Precision Medicine Multi-Omics Research, Institute of Advanced Clinical Medicine, Peking University, Beijing, China

**Keywords:** Computational models, Machine learning, Epigenomics, Chromatin remodelling, Chromatin analysis

## Abstract

Three-dimensional genome organization controls cell-type-specific gene expression through chromatin interactions, yet systematic analysis across diverse cellular contexts remains limited by experimental constraints. Here we present Hi-Compass, a depth-aware deep learning framework that predicts cell-type-specific chromatin organization using only chromatin accessibility data as cell-type-specific input. By dynamically accommodating variability in sequencing depth, Hi-Compass enables robust predictions across the full spectrum of data scales, from sparse single-cell to high-coverage bulk profiles. Benchmarking shows that Hi-Compass achieves superior concordance with experimental Hi-C data compared to existing methods, with particularly strong recovery of high-confidence chromatin loops. Applied to peripheral blood and embryonic heart datasets, Hi-Compass resolves cell-type-specific chromatin interactions and systematically links disease-associated variants to putative target genes. The framework further enables spatially resolved chromatin interaction prediction in hippocampal tissue and demonstrates cross-species applicability through fine-tuning to mouse systems. Hi-Compass expands the capacity to study three-dimensional genome regulation across biological scales and species.

## Abstract (web summary)

Cell-type-specific 3D genome maps are hard to generate experimentally. Here, the authors develop Hi-Compass, a deep-learning framework that predicts chromatin interactions from accessibility data across variable sequencing depths, recovering chromatin loops and linking disease variants to target genes.

## Introduction

The three-dimensional (3D) organization of the genome is a critical regulator of gene expression, cellular function, and tissue development^1–6^. High-throughput techniques, particularly chromosome conformation capture (3C)-based methods, such as Hi-C, have revolutionized our understanding of chromatin architecture by mapping interactions at genome-wide scales^7,8^. These studies have revealed hierarchical structures, including A/B compartments, topologically associating domains (TADs), and chromatin loops^9–17^, which collectively orchestrate cell-type-specific gene expression through enhancer-promoter interactions. Such insights are essential for understanding regulatory mechanisms underlying development and disease^6,18–21^.

Multiple factors contribute to the formation of 3D genome architecture. DNA sequences serve as the fundamental blueprint, with specific motifs directing nucleosome positioning, protein complex binding, and the establishment of regulatory domains^18,22,23^. Chromatin accessibility, which reflects the activity of regulatory elements, such as enhancers and promoters, further modulates chromosomal interactions^24–26^. Architectural proteins, including CTCF, play a pivotal role by facilitating chromatin looping and boundary formation^27–30^. The dynamic interplay of these elements creates cell-type-specific 3D organization patterns which are essential for maintaining cellular identity and function^31,32^.

Despite its transformative impact, Hi-C technology faces practical challenges. Generating high-resolution Hi-C data requires substantial resources, specialized expertise, and extensive sequencing depth^33,34^. Therefore, computational approaches that predict 3D genome structure have emerged as a valuable and feasible solution. For instance, DeepC^35^ employs transfer learning to predict chromatin interactions from DNA sequences leveraging epigenomic pre-training. Akita^36^ uses a convolutional neural network to predict interactions at 2 kb resolution from 1 Mb DNA sequences, accounting for distance-dependent decay. C.Origami^33^ represents a multimodal architecture integrating DNA sequences, bulk CTCF ChIP-seq and ATAC-seq signals through CNN layers and self-attention blocks. More recently, AlphaGenome^37^ was developed as a multi-task foundation model that predicts diverse genomic features including contact maps from DNA sequence alone. In contrast, Epiphany^38^ and ChromaFold^39^ predict contact maps solely from epigenomic information, bypassing DNA sequences, through a much lightweight model.

The rapid expansion of single-cell and spatial epigenomic profiling technologies presents a promising opportunity to extend 3D genome prediction models to these emerging data types. However, existing computational methods face critical limitations when applied to single-cell and spatial datasets: (1) Requirement for jointly profiled ATAC-seq, CTCF ChIP-seq and other epigenomics data from the same biological sample; (2) Limited capacity to generate cell-type-specific predictions for unseen cell types; (3) Failure to account for data sparsity inherent in single-cell and spatial omics; (4) Lack of fine-grained structural prediction supporting downstream analytical applications.

To address these limitations, we introduce Hi-Compass, a deep learning framework designed to accurately predict cell-type-specific Hi-C maps across diverse experimental conditions. Hi-Compass uniquely leverages (sc)ATAC-seq data as its sole cell type-aware input, combined with DNA sequence and a generalized CTCF binding profile, to infer structural details of 3D genome organization. The model dynamically adjusts to varying sequencing depths through a depth-aware module, enabling robust performance across diverse experimental settings, including bulk, single cell and spatial assays. Hi-Compass uses a multi-cell-type training strategy, enabling zero-shot generalization to unseen cell types. Benchmarking analyses demonstrate that Hi-Compass outperforms existing methods in prediction accuracy for bulk samples. Furthermore, extensive evaluations in single-cell and spatial contexts demonstrate the method’s capability to accurately predict cell-type-specific 3D genome structures across diverse biological scenarios, including immune cell differentiation, tissue heterogeneity and spatial organization. Moreover, Hi-Compass enables systematic annotation of long-range regulatory connections between disease-associated non-coding variants and their target genes, facilitating functional interpretation of genetic associations. Lastly, we show that Hi-Compass generalizes to the mouse genome through fine-tuning, enabling cross-species applications. By bridging accessible chromatin profile with 3D genome architecture, Hi-Compass expands the range of biological questions that can be addressed through Hi-C prediction models.

## Results

### Hi-Compass enables Hi-C prediction from multi-depth ATAC-seq data

**Fig. 1:**
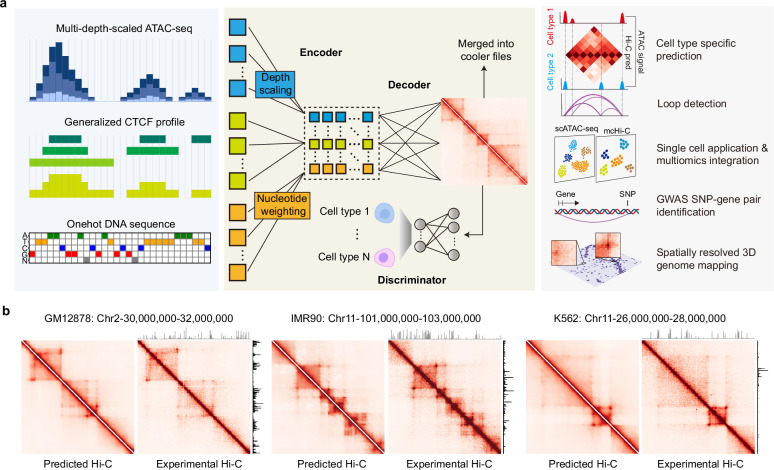
Hi-Compass predicts 3D genome structure in a sequencing depth-aware manner. **a** Overview of the Hi-Compass methodology framework. The model takes three types of sequence information as input to predict corresponding Hi-C structures, integrates them into whole-chromosome.cool files, and enables different downstream analysis scenarios for various applications. **b** Comparison between Hi-Compass-predicted Hi-C and experimental ground truth Hi-C for GM12878, IMR90 and K562 in representative regions of the validation set (Chr2) and test set (Chr11). The plot on the right side of the heatmap depicts the chromatin accessibilities of the corresponding regions. The plot on the top depicts the generalized CTCF binding signals.

To address the challenge of predicting 3D genome structure from single-cell chromatin accessibility data, we developed Hi-Compass (Fig. 1a), a deep learning model integrating four key inputs: ATAC-seq signal, ATAC-seq sequencing depth, DNA sequence and a generalized CTCF binding profile. This multi-modal input design allows the model to understand both the general principles of genome folding from static genomic features and cell-type-specific genome folding from chromatin accessibility patterns. Hi-Compass is designed to adapt to ATAC-seq data of varying sequencing depths, enabling the generation of high-quality, cell-type-specific Hi-C predictions without requiring additional experimental data.

To overcome the limited availability of single-cell CTCF ChIP-seq data, we used a generalized CTCF binding profile derived from pan-tissue samples. Specifically, we integrated bulk CTCF ChIP-seq data from 165 human samples (Supplementary Data 1) and constructed a comprehensive CTCF binding map through systematic peak integration. This pan-tissue reference map preserves fundamental CTCF binding principles, serving as a framework to guide ATAC-seq signals in identifying cell-type-specific CTCF binding patterns. For DNA sequence, we employed a five-channel one-hot encoding representing A, T, C, G, and N.

The core architecture of Hi-Compass combines CNN and Transformer modules (Supplementary Fig. 1). At the input layer, three encoders composed of convolutional layers extract features from DNA sequences, ATAC-seq signals, and generalized CTCF binding signals. These features are integrated to provide a comprehensive representation of genomic information. The ATAC encoder includes a scaling module that adjusts for total sequencing depth, enhancing the model’s adaptability to varying depths through learnable parameters. The decoder employs a Transformer architecture to process the extracted genomic features and generates the final two-dimensional Hi-C matrix. To improve cell-type-specific prediction accuracy, Hi-Compass incorporates a discriminator network that evaluates the cell-type specificity of the output. Prior to training, Hi-C contact matrices are preprocessed using a contrast stretching normalization approach. Raw Hi-C contact matrices typically exhibit low contrast with interaction signals concentrated in a narrow intensity range, which limits the model’s ability to learn fine-scale chromatin structures. Our preprocessing procedure rescales each chromosomal contact matrix relative to its 98th percentile, effectively expanding the contrast while preserving the relative differences between strong and weak interactions. This normalization enhances the visibility of biologically relevant chromatin features without distorting the underlying structural patterns (Supplementary Fig. 1). The loss function combines mean-squared error (MSE) and information-weighted structural similarity^40,41^ (SSIM) to supervise numerical accuracy and structural features, along with the discriminator’s cross-entropy (CE) to optimize cell type specificity.

Hi-Compass predicts Hi-C fragments of 2 Mb in size at 10 kb resolution. Hi-Compass predictions reproduced the topological structural details of experimental Hi-C with high fidelity across different cell types and genomic regions (Fig. 1b and Supplementary Fig. 2). By sliding the prediction window along chromosomes and adopting an optimized stitching strategy, we built a complete pipeline (Fig. 1a) capable of generating genome-wide Hi-C map predictions. These predictions are integrated into standard cool format files for subsequent analyses, such as insulation score calculation and loop identification, by using standard tools.

Since only matched bulk ATAC-seq and Hi-C data are currently available, we developed a computational strategy to simulate multi-depth ATAC-seq profiles spanning a continuum from single-cell to bulk-level resolution. To determine the optimal threshold, we systematically explored the impact of sequencing depth on signal preservation. Depth reduction preserves most genomic features from bulk sequencing profiles (Supplementary Fig. 3a), with high reproducibility observed between different sampling replicates at equivalent depths (Supplementary Fig. 3b, c) until reaching critical depth threshold. Based on these assessments, we implemented a minimum ATAC-seq depth threshold of 2e5 reads (~ 0.01X genome coverage) during model training to ensure robust and specific ATAC-seq signals.

We curated 14 human high-quality matched Hi-C and ATAC-seq datasets (Supplementary Data 2) covering multiple tissue types. To ensure rigorous evaluation, we implemented a dual-axis data splitting strategy: cell types were divided into training (six samples) and test groups, while chromosomes were partitioned into training, validation (Chr2), and test (Chr11) sets (Supplementary Fig. 4a and Supplementary Data 3).

### Systematic evaluation of Hi-Compass performance

**Fig. 2:**
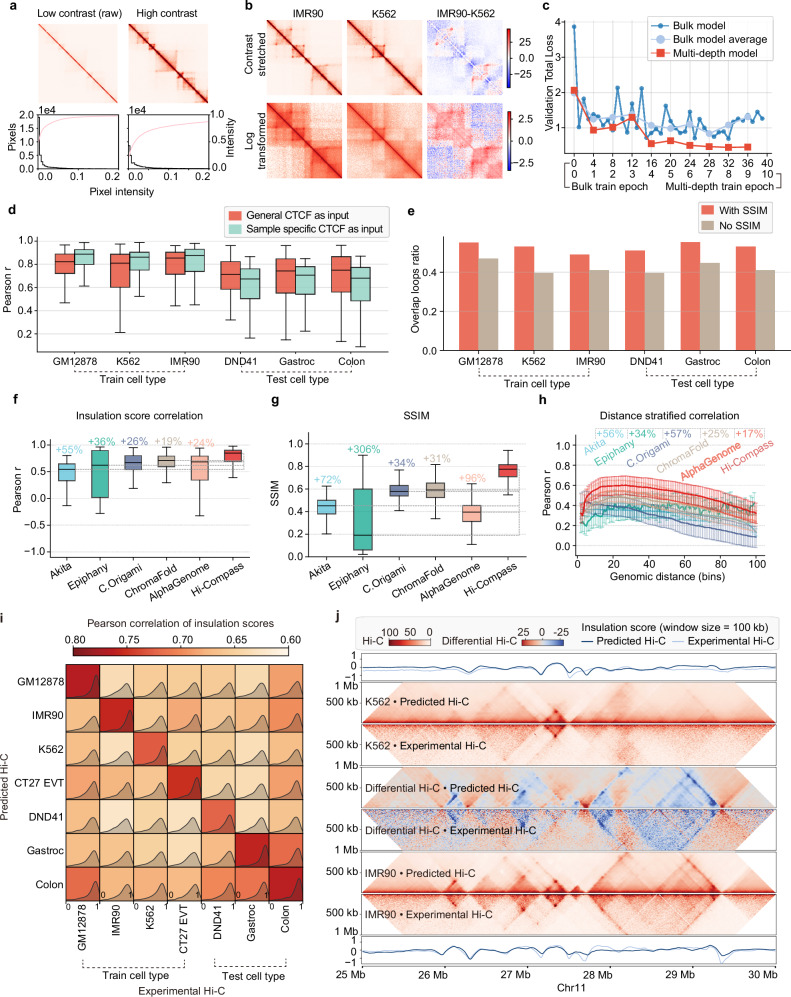
Systematic evaluation of Hi-Compass performance. **a** Comparison of raw and contrast-stretched Hi-C matrices with corresponding grayscale histograms and cumulative distribution curves. **b** Comparison of contrast stretching and log transformation for IMR90 and K562 at Chr11 26.5–28.5 Mb, including differential Hi-C matrices. **c** Validation loss curves for bulk-only versus multi-depth training, with x-axes normalized to equivalent iteration counts. **d** Insulation score correlations on test chromosomes using general versus cell-type-specific CTCF ChIP-seq input. The first three samples are training cell lines; the last three are novel cell lines (*n* = 1215 segments per sample). **e** Overlap ratio of predicted and experimental Hi-C loops, comparing models with and without SSIM loss. **f–h** Performance comparison with Akita, Epiphany, C.Origami, ChromaFold and AlphaGenome, showing insulation score correlation (**f**), SSIM (**g**) and distance-stratified correlation (**h**). In (**f** and **g**), *n* = 7840 for Akita, 2566 for AlphaGenome, 1058 for Epiphany, 1011 for C.Origami, 1253 for ChromaFold, 1215 for Hi-Compass. In (**h**), lines and shaded bands represent mean and standard deviation. **i** Heatmap of insulation score correlations across chromosomes between predictions and experimental Hi-C. The first four samples are from the training set; the last three are from the test set. Window size = 10 bins, first two diagonals excluded. Gastroc: Gastrocnemius; Colon: Transverse colon; CT27 EVT: CT27 Extravillous Trophoblast. **j** Predicted (upper triangle) versus experimental (lower triangle) Hi-C for K562 and IMR90 at a 5 Mb region on Chr11, with differential heatmaps and insulation score profiles (window size = 10 bins). In all box plots (**d**,**f**,**g**), the center line represents the median, box bounds the 25 and 75th percentiles, and whiskers extend to 1.5 times the interquartile range.

To understand the contribution of different components in Hi-Compass, we conducted systematic ablation studies examining key aspects of our methodology. We evaluated the impact of our image preprocessing approach by comparing contrast stretching normalization against log transformation on experimental Hi-C data (Fig. 2a, b). Contrast stretching enhanced the visibility of fine-scale chromatin structures while preserving global interaction patterns, as demonstrated by improved contrast and structural clarity in representative genomic regions. The pixel intensity histograms showed that contrast stretching achieved better normalization of signal distribution compared to raw data, particularly for detecting and enhancing subtle but biologically relevant interactions (Fig. 2a). Visual comparison of experimental Hi-C maps between IMR90 and K562 cell lines revealed that contrast stretching better preserved cell type-specific differences in chromatin organization, with markedly improved discrimination between the two cell types (Fig. 2b).

We next assessed the effectiveness of our multi-depth training strategy by comparing models trained exclusively on bulk ATAC-seq data versus those incorporating multi-depth sampling approach (Fig. 2c). The multi-depth training not only conferred adaptability across the full spectrum of sequencing depths (from sparse single-cell to high-coverage bulk data) but also demonstrated superior training dynamics, as evidenced by faster convergence and reduced final validation loss compared to bulk-only training. Quantitative analysis revealed that the multi-depth approach achieved equivalent performance with fewer training epochs when normalized by iteration count, suggesting that exposure to depth variation during training enhances both model robustness and learning efficiency.

We further investigated the impact of CTCF binding specificity by comparing prediction performance using cell-type-specific CTCF ChIP-seq data versus our generalized CTCF profile (Fig. 2d). Although cell-type-specific CTCF data yielded marginally higher correlation coefficients for training cell types, the generalized profile showed significantly improved robustness when applied to novel cell types, demonstrating superior performance on unseen samples. These results validate our implementation of a generalized CTCF binding profile, which maintains competitive accuracy for known cell types while exhibiting enhanced generalizability to cell types lacking CTCF ChIP-seq data. This design choice substantially expands Hi-Compass’s applicability across diverse biological systems and experimental conditions.

Lastly, we examined the contribution of the SSIM component in our loss function by comparing model performance with and without it (Fig. 2e). The SSIM-incorporated models consistently demonstrated enhanced performance in chromatin loop detection, showing 5–15% higher overlap ratios with experimental Hi-C data across all tested samples. This improvement highlights SSIM’s crucial role in maintaining the structural fidelity of predicted chromatin interactions that are essential for accurate loop identification. Taken together, these results establish that all components of Hi-Compass contribute synergistically to its robust prediction performance, with the multi-depth training strategy and SSIM-based loss function providing the most significant gains in both prediction accuracy and biological interpretability.

To comprehensively evaluate Hi-Compass generalization across different experimental contexts, we analyzed model performance stratified by both cell type and chromosome category. Predictions on held-out test chromosome (Chr11) across test cell types represented the most challenging scenario. Notably, even under this stringent evaluation setting, Hi-Compass consistently maintained robust performance with insulation score correlations exceeding 0.6 (Supplementary Fig. 4b). We then conducted distance-stratified correlation analysis, calculating the correlation between predictions and experimental data at varying genomic distances (Supplementary Fig. 5). Across all tested samples, Hi-Compass exhibited stable prediction accuracy. This performance demonstrates that Hi-Compass can reliably resemble chromatin interactions across diverse cell types, effectively learning generalizable principles of chromatin organization rather than simply memorizing training examples.

Building on these validations, we then systematically compared Hi-Compass with state-of-the-art Hi-C prediction methods, including Akita^36^, C.Origami^33^, Epiphany^38^, ChromaFold^39^, and AlphaGenome^37^. To ensure a fair evaluation, we used identical test datasets and assessment metrics for all methods, with insulation scores calculated at each method’s native resolution (2 kb for Akita and AlphaGenome; 10 kb for others). Insulation score^42^ correlation, SSIM and distance-stratified correlation analysis were applied as the evaluation metric due to their ability to effectively capture chromatin structural features and their robustness to batch effects^33^.

Hi-Compass significantly outperformed other methods in insulation score correlation with experimentally Hi-C data (Fig. 2f–h). In genome-wide evaluations based on 2 Mb windows, Hi-Compass achieved a median Pearson correlation coefficient >0.7, representing a 19% improvement over the second-best performing method (Fig. 2f). SSIM analysis further confirmed Hi-Compass’s superior ability to preserve chromatin structural features, with median scores >0.7, representing a 31% increase compared to the next best alternative method (Fig. 2g). Distance-stratified correlation analysis revealed that Hi-Compass maintained consistent prediction accuracy across genomic distances from 10 kb to 1 Mb, demonstrating robust performance at both local and long-range chromatin interactions (Fig. 2h). These performance advantages were consistent when evaluated using Spearman correlation (Supplementary Fig. 6a, b).

To visually demonstrate the performance of Hi-Compass, we selected multiple representative genomic regions and compared Hi-Compass predictions side-by-side with those from existing methods (Supplementary Fig. 7). These regions cover diverse chromatin structural features, including TAD boundaries and local interaction patterns. Visual comparisons clearly revealed that Hi-Compass-generated Hi-C matrices closely resembled experimentally measured Hi-C data, particularly in capturing fine-scale structural details. In contrast, while other methods captured major structural features, they often exhibited loss of microstructure details, artificial segmentations and blurred profiles.

### De novo inference of cell-type-specific Hi-C maps with visible chromatin loop structures

Having established Hi-Compass’s overall prediction accuracy and generalizability, we next evaluated its ability to capture cell-type-specific chromatin organization. Using bulk ATAC-seq data from diverse samples, we predicted their genome-wide Hi-C maps and calculated the correlation of insulation scores between predictions and experimental data. Hi-Compass predictions exhibited strong cell type specificity, with high correlations along the diagonal of the heatmap (Fig. 2i, Supplementary Fig. 6c, Supplementary Fig. 8a and Supplementary Fig. 9).

Visual comparisons of Hi-C maps revealed that Hi-Compass predictions not only achieved high overall accuracy but also precisely preserved cell-type-specific structural features (Fig. 2j). The predicted maps closely matched the corresponding experimental data, accurately capturing key features, such as compartmental structures, TAD boundaries, and chromatin loops. Notably, these genome-wide Hi-C maps were constructed by stitching multiple 2 Mb prediction windows along chromosomes, yet the resulting maps showed no visible artifacts or discontinuities at the junction boundaries, demonstrating Hi-Compass’s remarkable consistency and spatial coherence in chromatin interaction prediction across large genomic scales.

To quantitatively assess the accuracy of chromatin domain predictions, we evaluated TAD boundary calling performance using experimental Hi-C data as ground truth (Supplementary Fig. 8b). Across all tested samples, Hi-Compass achieved overall TAD boundary recall rates exceeding 65%, demonstrating reliable identification of topological domain structures.

The high-quality predictions of Hi-Compass enable robust downstream analyses. We performed loop detection on both Hi-Compass-predicted and experimental Hi-C data and analyzed their overlap (Supplementary Fig. 8c). The average loop prediction consistency approached the 60% reported between high-quality experimental replicates of GM12878^18^. We further examined the loop detection performance across different cell types (Supplementary Fig. 8d). The number of loops identified from predicted Hi-C showed consistent trends with that from experimental Hi-C across cell types, suggesting that Hi-Compass captures cell-type-specific differences in chromatin loop abundance. Stratified analysis by statistical significance of loops from experimental Hi-C revealed that Hi-Compass achieved substantially higher recovery rates for high-confidence loops. At a stringent FDR cutoff (FDR < 0.01), Hi-Compass recovered approximately 75% of experimentally identified loops, indicating its ability to preferentially capture the most robust chromatin interactions.

To further validate that Hi-Compass predictions capture protein-mediated chromatin contacts, we performed aggregate peak analysis (APA)^18^ using two orthogonal cohesin-mediated interaction datasets in GM12878: cohesin HiChIP loops (*n* = 5337)^43^ and RAD21 ChIA-PET loops (*n* = 5983)^44^. Both experimental and Hi-Compass-predicted Hi-C contact maps exhibited clear focal enrichment at the positions of these loops (Supplementary Fig. 10), confirming that Hi-Compass predictions are consistent with chromatin interactions independently identified by protein-directed conformation capture technologies.

### Cross-species generalization through fine-tuning

To assess the cross-species applicability of Hi‑Compass, we collected another four matched ATAC‑seq and Hi‑C datasets from mouse cell lines (Supplementary Data 2). Using a transfer learning strategy, we fine‑tuned the pre‑trained human model on mouse data, training on two mouse cell types (TT2 and mESC) and testing on two additional cell types (Patski and Retina) (Supplementary Fig. 11a). Chromosomes 15–17 were reserved for validation and chromosomes 18–19 as the test set.

After fine-tuning, Hi-Compass demonstrated robust predictive performance on mouse data, achieving insulation score correlations comparable to its results in human samples (Supplementary Fig. 11b). Distance-stratified correlation analysis further confirmed consistent prediction accuracy across genomic distances in mouse (Supplementary Fig. 12). These findings demonstrate that the chromatin folding principles learned by Hi-Compass are transferable between human and mouse, and that the model has the potential to adapt to new organisms through fine-tuning with limited species-specific training data.

### Hi-Compass overcomes single-cell data sparsity **for** robust Hi-C prediction

Building on Hi-Compass’s high performance with bulk data, we extended its application to single-cell settings. Leveraging insights from our ATAC-seq depth analysis (Supplementary Fig. 3), we implemented a meta-cell strategy to address the inherent sparsity of single-cell data (Supplementary Fig. 13a). Specifically, we integrated ATAC-seq signals from adjacent single cells into meta-cells, ensuring that the input data met the depth threshold required for accurate, cell-type-specific predictions.

**Fig. 3:**
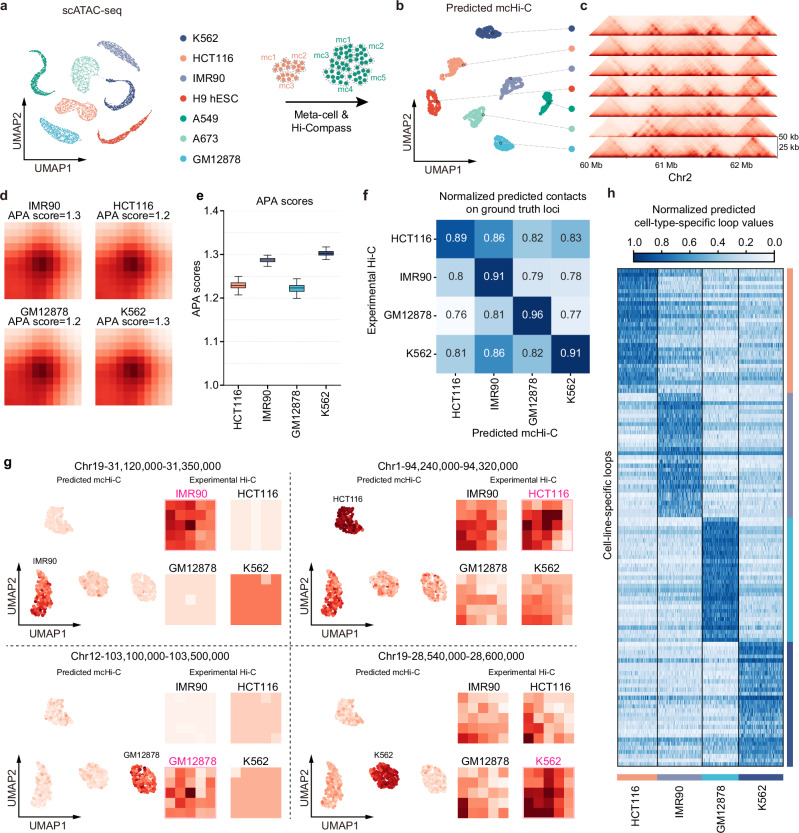
Hi-Compass predicts cell-type-specific 3D chromatin interactions using scATAC-seq. **a** UMAP projection of scATAC-seq profiles of seven cell lines. **b** UMAP visualization of predicted mcHi-C. **c** Example predicted mcHi-C on a genomic region from Chr2. Lateral Hi-C plot showing one meta cell profile per cell type. **d** APA plots show the aggregate signals of predicted mcHi-C at loop sites detected from experimental Hi-C in four cell lines (IMR90, HCT116, GM12878, K562). The plots are generated by summing submatrices of predicted contacts of mcHi-C surrounding loop peaks detected in experimental Hi-C data. **e** Box plots showing the distribution of APA scores across meta cells for each cell line (*n* = 182 for IMR90, 185 for HCT116, 150 for GM12878, 181 for K562). The center line represents the median, box bounds represent the 25 and 75th percentiles, and whiskers extend to 1.5 times the interquartile range. **f** Cross-validation of cell-type-specificity depicted by normalized predicted contacts at loop peaks detected from experimental Hi-C across cell lines. **g** Representative cell line-specific loops visualized by feature plots of predicted mcHi-C data and loop signal plots of experimental Hi-C data in four cell lines. Each panel shows one cell line-specific chromatin loop as example. Feature plot (left) displays the normalized predicted contacts at the loop peak in each of all meta cells. Loop signal plot (right) displays the experimental Hi-C signals at the same loop peak. **h** Heatmap displays normalized predicted contacts at cell line-specific loop peaks. Each row represents an individual loop peak, while columns represent predicted contacts at loop peaks across different cell lines.

We applied Hi-Compass to a scATAC-seq dataset comprising seven cell lines (Supplementary Data 4), four of which (IMR90, GM12878, K562, and HCT116) had corresponding bulk Hi-C data available (Fig. 3a). Meta-cell Hi-C (mcHi-C) was predicted from meta-cell ATAC-seq, with each meta-cell containing an average of ten cells (range: 5–15) to ensure a basic ATAC-seq depth for Hi-Compass. We extracted the first 50 diagonals from each predicted Hi-C matrix as feature vectors, performed PCA dimensionality reduction, and visualized the results using UMAP. The mcHi-C predictions from the same cell line formed distinct clusters (Fig. 3b), confirming the high consistency and cell type specificity of Hi-Compass predictions. Additionally, the Hi-C predictions for each meta-cell showed clear chromatin structures and cell-type-specific patterns across the genome, as illustrated in a representative genomic region (Fig. 3c).

To quantitatively assess Hi-Compass’s performance, we employed APA. First, we called chromatin loops in the four matched experimental Hi-C datasets. These loop coordinates were then mapped to Hi-Compass predictions, and local contact matrices were extracted around each loop peak. APA profiles were generated by superimposing these matrices, revealing significant signal enrichment at actual loop peaks, with contact frequencies at the center point markedly higher than in surrounding regions (Fig. 3d). These results showed strong concordance with the APA profiles derived from experimental Hi-C data (Supplementary Fig. 13b). Importantly, each independent mcHi-C prediction achieved strong APA scores (APA score > 1) (Fig. 3e), with high consistency across meta-cell predictions from the same cell line.

Next, we sought to investigate whether Hi-Compass could accurately predict cell-type-specific chromatin loops. APA analysis was performed on cell-type-specific loops in the four cell lines, revealing that predicted profiles exhibited the strongest signals at their corresponding true loop peaks (Fig. 3f and Supplementary Fig. 14a). Predicted loop peak signals were highly consistent within the same cell line and aligned well with experimental data, as illustrated by representative loops (Fig. 3g and Supplementary Fig. 14b). Further analysis revealed that over half of the cell-type-specific loops identified from experimental Hi-C were precisely captured by mcHi-C predictions with statistical significance (Mann-Whitney U test, effect size > 0.6, *p* < 0.05) (Fig. 3h and Supplementary Fig. 14c). These results collectively validate Hi-Compass’s reliability and robustness in predicting cell-type-specific chromatin structures from sparse scATAC-seq data, establishing its utility for single-cell 3D genomic applications.

### Hi-Compass reconstructs cell type chromatin dynamics in complex tissues

**Fig. 4:**
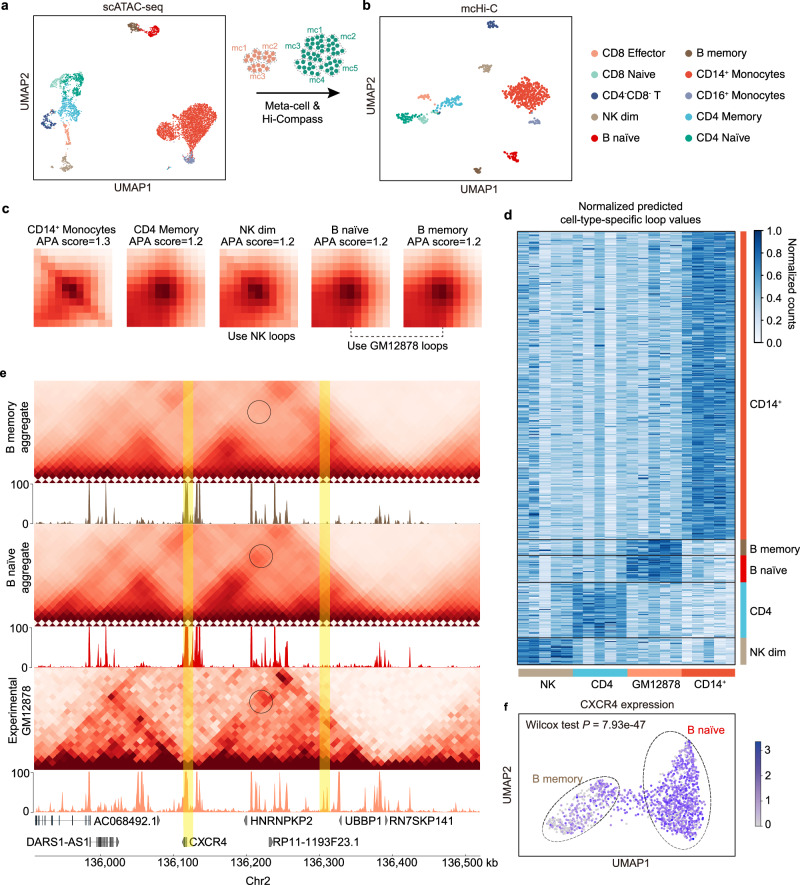
Hi-Compass generates accurate cell-type-specific prediction in complex tissues. **a** UMAP projection of PBMC scATAC-seq profiles, with different colors representing immune cell subtypes. **b** UMAP visualization of predicted mcHi-C. **c** APA plots show the aggregate signals of predicted mcHi-C at loops detected from experimental Hi-C in four representative immune cell types (CD14^+^ Monocytes, CD4 Memory, NK, and GM12878). The plots are generated by summing submatrices of predicted contacts of mcHi-C surrounding loop peaks detected in experimental Hi-C data. **d** Heatmap displays normalized predicted contacts at cell-type-specific loop peaks. Each column represents an individual loop peak, while rows represent predicted contacts at loop peaks across different cell types. **e** Predicted Hi-C profiles and corresponding ATAC-seq signal tracks for B cell subtypes and GM12878 in the CXCR4 region of Chr2. The loop anchored at CXCR4 TSS is marked in black. Loop anchors are highlighted in yellow. **f** Feature plot showing CXCR4 expression levels within two B cell subtypes.

To evaluate the performance of Hi-Compass in complex tissues, we applied it to a peripheral blood mononuclear cells (PBMC) scATAC-seq dataset^45^. Cell type annotations were assigned using label transfer from an annotated PBMC scRNA-seq dataset. After filtering out cell types with insufficient cell numbers, we retained 10 major immune cell subtypes for subsequent analysis (Fig. 4a). A similar meta-cell strategy followed by Hi-Compass were applied. The predicted mcHi-C effectively preserved the hierarchical relationships of immune cell types, as visualized by UMAP. Meta-cells within the same cell type exhibited strong correlations while maintaining distinct separation from other cell types (Fig. 4b). These results were not biased by variations in ATAC-seq sequencing depth across meta-cells (Supplementary Fig. 15a). To evaluate prediction accuracy, we collected publicly available bulk Hi-C data for four key immune cell types: CD4 Memory T cells, CD14^+^ monocytes, NK cells (corresponding to NK dim), and GM12878 (corresponding to B naïve and B memory cells). These verification datasets encompassed major immune cell lineages and diverse functional characteristics, providing an ideal reference for assessing Hi-Compass’s performance in complex immune microenvironments. APA analysis at 10 kb revealed significant enrichment of predicted mcHi-C signals at true loop peaks across immune cell subtypes (Fig. 4c and Supplementary Fig. 15b), with APA scores consistently exceeding 1 for all meta cells (Supplementary Fig. 16a, b). Further analyses unveiled that Hi-Compass accurately recovered cell-type-specific loop signals (Fig. 4d and Supplementary Fig. 16c, d).

To elucidate the capability of Hi-Compass in detecting 3D genome dynamics in closely related cell states, we examined the predicted mcHi-C profiles of two B cell states: B memory and B naïve. GM12878 experimental data served as a reference. We focused on dynamic chromatin reorganization around key factors in B cell differentiation. Hi-Compass predictions identified a cell state-specific chromatin loop connecting the CXCR4 promoter with a distal regulatory element in naïve B cells that was absent in memory B cells (Fig. 4e). Notably, while chromatin accessibility at the regulatory region showed subtle differences between these cell states, the presence of this Hi-C interaction in naïve B cells correlated strongly with significantly higher CXCR4 expression levels (Fig. 4f), validating the functional relevance of the predicted conformation change. These results demonstrate Hi-Compass’s sensitivity in detecting fine-scale but biologically significant 3D genome reconfigurations that underlie gene expression differences in closely related cell states.

As a critical chemokine receptor involved in B cell development, homing, and tissue distribution, CXCR4 exhibited cell-state-specific chromatin conformation changes that likely reflect its fine-tuned transcriptional regulation^46–48^. This multi-omics integrated analysis not only showcases Hi-Compass’s utility in addressing biological questions but also demonstrates its capability to reliably predict 3D genomic structures from scATAC-seq data in novel cell states.

### Hi-Compass resolves chromatin states in mouse T cell subtypes

To further demonstrate the broad applicability of Hi‑Compass across mammalian systems, we applied the fine-tuned mouse model to a published scATAC-seq dataset of mouse splenic CD4^+^ T cells^49^. Based on Foxp3 gene activity, we classified cells into two functionally distinct subtypes: regulatory T cells (Treg, Foxp3^+^) and conventional T cells (Tcon, Foxp3^-^) (Supplementary Fig. 17a, b). These two populations represent critical components of immune homeostasis, with Tregs playing essential roles in maintaining self-tolerance and preventing autoimmunity.

Using our established meta‑cell strategy, we predicted mcHi-C for both T cell subtypes. UMAP visualization of the predicted mcHi-C profiles demonstrated clear separation between Treg and Tcon cells (Supplementary Fig. 17c), indicating that Hi-Compass captures subtype-specific chromatin organization patterns even in closely related cell subtypes. Similar to our observations in human PBMC data, we evaluated prediction accuracy using matched experimental Hi-C data from mouse T cell subtypes. APA analysis confirmed that predicted mcHi-C signals showed significant enrichment at experimentally validated loop peaks, with APA scores exceeding 1.1 for both subtypes (Supplementary Fig. 17d). Analysis of cell-type-specific chromatin loops revealed distinct interaction patterns between Treg and Tcon cells (Supplementary Fig. 17e), and representative subtype-specific loops were visualized showing clear differential signals between the two populations (Supplementary Fig. 17f). These results demonstrate that the fine-tuned Hi-Compass model retains high performance on mouse single-cell data, providing a powerful approach for investigating chromatin‑based mechanisms in mammal.

### Hi-Compass integrates single-cell multiome data to predict functional associations

**Fig. 5:**
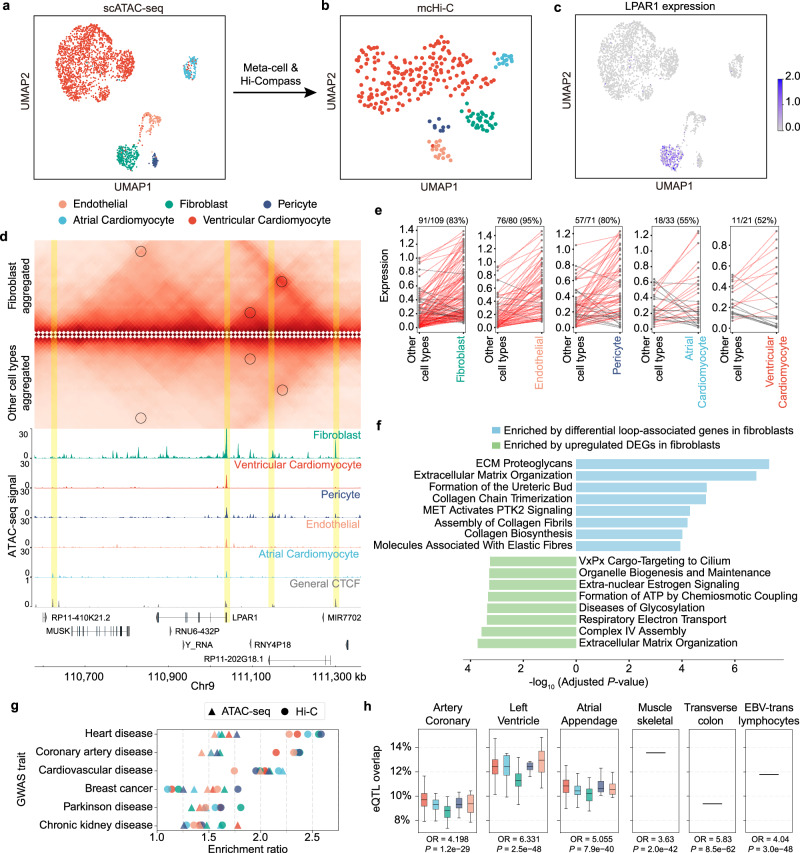
Hi-Compass integrates single-cell multiome data to reveal functional associations. **a** UMAP projection of scATAC-seq profile from human embryonic heart tissue, with different colors representing distinct cell types. **b** UMAP visualization of predicted mcHi-C, demonstrating the prediction preserved the original cell type distribution pattern. **c** Feature plot showing LPAR1 is specifically expressed in fibroblasts. **d** Predicted Hi-C profiles and corresponding ATAC-seq signal tracks for fibroblasts versus other cell types in the LPAR1 genomic region. The loops anchored at LPAR1 TSS are marked in black. Loop anchors are highlighted in yellow. **e** Genes associated with cell-type-specific loops are frequently upregulated in corresponding cell types (marked by red lines). Genes whose promoters located in a cell-type-specific loop anchor are included in the analysis. **f** Reactome pathway enrichment analysis of differential loop-associated genes (blue) and upregulated DEGs (green) in fibroblasts. **g** Enrichment analysis of GWAS variants across disease categories. Dot plot showing enrichment scores for cardiac versus non-cardiac traits, comparing SNP annotations based on: loop anchors from predicted Hi-C (triangular) versus ATAC-seq peaks (circular). Cardiac disease categories show significantly stronger enrichment for loop-annotated variants. **h** Validation of SNP-gene regulatory links against GTEx eQTL data. Box plots showing overlap percentages between loop-annotated SNP-gene links and eQTL data in different tissues. The left three panels display results using Hi-Compass-predicted Hi-C data, while the right three show results derived from experimental bulk Hi-C data. Each box represents the distribution of eQTL overlap percentages across meta cells within each cell type (*n* = 188 for Ventricular Cardiomyocyte, 14 for Atrial Cardiomyocyte, 31 for Fibroblast, 8 for Pericyte, 17 for Endothelial). The center line represents the median, box bounds represent the 25 and 75th percentiles, and whiskers extend to 1.5 times the interquartile range; outliers are not shown. Statistical significance of SNP-to-gene links was assessed using two-sided Fisher’s exact test.

To further validate Hi-Compass’s utility, we applied it to a single-cell multiome dataset that sequenced a 120-day female human embryonic heart tissue. The data contains jointly profiled gene expression and chromatin accessibility in the same single cells. We processed the data using standard procedures, and annotated cell types based on gene expression profiles. Unsupervised clustering of scATAC-seq data revealed high consistency with transcriptome-annotated cell classifications (Fig. 5a), confirming the internal coherence of the dataset. Following the previous workflow, we generated mcHi-C predictions from scATAC-seq data. UMAP dimensionality reduction demonstrated that predicted mcHi-C maintained distribution patterns similar to scATAC-seq data (Fig. 5b), indicating that Hi-Compass successfully captured chromatin conformation characteristics across different cell types. We then identified differential loops for each cell type and integrated gene expression data from the same cells to validate the biological relevance and reliability of Hi-Compass predictions.

Taking the fibroblast marker gene LPAR1 as an example, it exhibited significantly higher expression in fibroblasts compared to other cell types (Fig. 5c). Hi-Compass predictions revealed fibroblast-specific chromatin loops anchored at the LPAR1 TSS (Fig. 5d). These loops were supported by cell-type-specific ATAC peak signals, with chromatin accessibility at loop anchors significantly higher in fibroblasts. Similar biologically interpretable loops were predicted across other cell types. For instance, in endothelial, the PTPRM gene region displayed cell-type-specific chromatin interactions (Supplementary Fig. 18a), consistent with its high expression in these cells (Supplementary Fig. 18b). Similarly, atrial cardiomyocytes exhibited cell-type-specific chromatin conformations in the MAPK4 gene region (Supplementary Fig. 18c), corresponding to its cell-type-specific expression pattern (Supplementary Fig. 18d). Systematic analysis of all cell-type-specific loops revealed that genes associated with these loops generally exhibited higher expression in their corresponding cell types (Fig. 5e). This observation supports the expected relationship between cell-type-specific chromatin structures and gene expression regulation, further validating the accuracy of Hi-Compass predictions.

By comparing the overlap of predicted mcHi-C loops across cell types, we identified unique chromatin interaction patterns that may indicate functional associations (Supplementary Fig. 19a). The shared loops between fibroblasts and pericytes were the second most abundant, surpassed only by those between two cardiomyocyte types, suggesting potential interconnected regulatory networks between the two cell types in embryonic heart development. Pathway analysis of differentially expressed genes (DEGs) in fibroblasts and fibroblast-specific chromatin loop-associated genes both revealed extracellular matrix (ECM) organization as the top enriched pathway (Fig. 5f). Intersectional analysis identified 22 genes shared between fibroblast-specific loop-associated genes and DEGs, including critical ECM components (COL14A1, FBN1), transcriptional regulators (TBX18, GLI2, GLIS3), and cell migration and adhesion molecules (DCC, CDH19) (Supplementary Fig. 19b). Notably, loop-associated genes were uniquely enriched for fiber-associated pathways, suggesting direct cell identity regulation through 3D genome organization. These results validate Hi-Compass’s capability to accurately predict chromatin structures and their associations with gene regulation in single-cell multiome datasets.

### Hi-Compass enables systematic annotation of disease variant-gene associations

Beyond predicting cell-type-specific regulatory networks, we evaluated the utility of Hi-Compass for annotating disease-associated genetic variants. Using predicted chromatin interactions from embryonic heart tissue, we systematically analyzed SNP-to-gene regulatory relationships by connecting non-coding regulatory elements with gene promoters through predicted loops. Enrichment analysis of loop anchors from predicted Hi-C on GWAS variants demonstrated expected specificity for cardiac-related disorders (Fig. 5g). For each disease trait, we calculated the enrichment ratio defined as the proportion of GWAS SNPs located within one anchor of a predicted chromatin loop whose paired anchor contained a gene promoter. Heart disease, coronary artery disease, and cardiovascular disease showed significantly higher enrichment than non-cardiac traits. Crucially, for these cardiac traits, variants annotated via chromatin loops derived from predicted Hi-C showed substantially stronger enrichment than those annotated solely based on ATAC-seq peaks, highlighting the added functional resolution provided by 3D chromatin architecture in prioritizing disease-associated non-coding variants.

We next compared Hi-Compass-derived SNP-to-gene links with tissue-specific expression quantitative trait loci (eQTLs) from the Genotype-Tissue Expression (GTEx) project. In artery coronary, left ventricle, and atrial appendage, Hi-Compass-derived links showed an average of 9%, 13%, and 11% overlap rates with eQTLs, respectively (Fig. 5h). These numbers are very close to the results obtained by using experimental bulk Hi-C, confirming the high fidelity of our predictions.

Further investigation revealed specific cases of cardiac disease-associated variants physically connected to known heart disease-related genes^50–54^ through Hi-Compass-derived loops (Supplementary Fig. 20a–c). We identified regulatory connections linking GWAS variants to established cardiac disease genes, including CDK8, FLRT2, and SSPN. These SNP-to-gene regulatory relationships are supported by both Hi-Compass-derived chromatin interactions and heart tissue eQTL evidence, suggesting potential role of these non-coding variants in cardiac pathology. These findings validate Hi-Compass’s utility for systematic annotation of regulatory connections involving disease-associated variants, providing a computational framework for linking genetic associations to potential target genes.

### Hi-Compass reconstructs spatially resolved Hi-C maps

Spatial omics technologies have rapidly developed in recent years^55–57^, with spatial transcriptomics making significant progress^58,59^ and spatial ATAC-seq emerging as a powerful tool^60,61^. However, the lack of spatial 3D genomic technologies has hindered our understanding of spatially organized chromatin regulatory networks. Here, we evaluate the capability of Hi-Compass to predict spatially resolved 3D Hi-C maps from spatial ATAC-seq data.

**Fig. 6:**
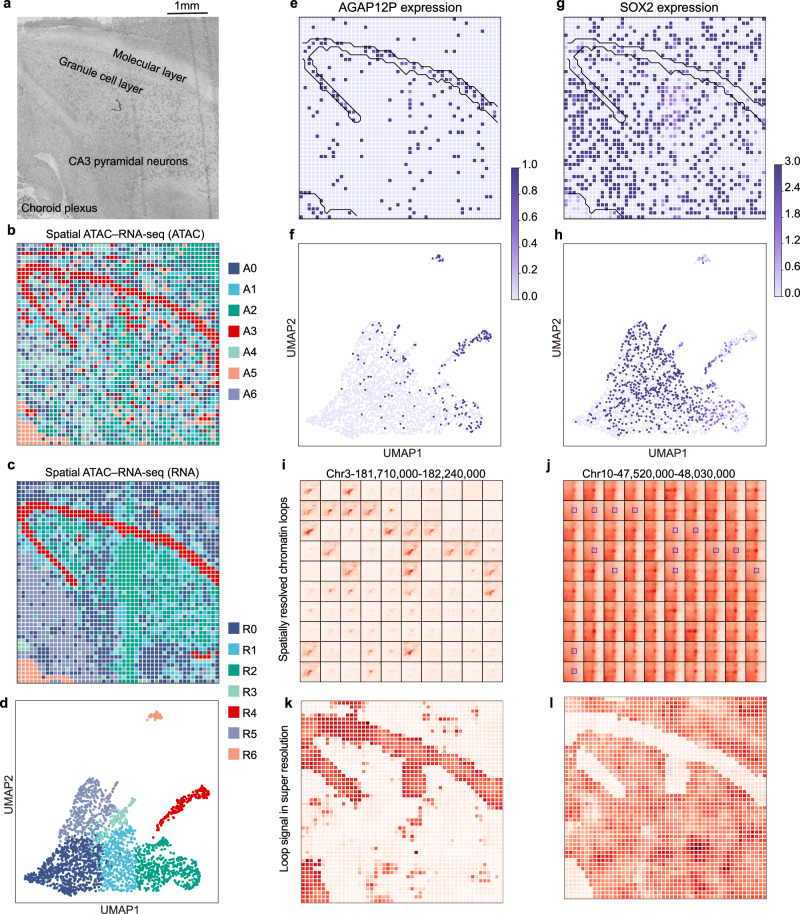
Hi-Compass reconstructs spatially resolved Hi-C maps. **a** H&E staining image of an adult male hippocampal tissue section, displaying histological characteristics of the granule cell layer, molecular layer, choroid plexus and other regions. The image was obtained from published spatial ATAC-RNA-seq data^61^. Scale bar, 1 mm. **b**,**c** Spatial distribution of ATAC-seq (**b**) and RNA-seq (**c**) clusters, with different colors representing distinct ATAC (A0–A6) and RNA (R0–R6) clusters. Granule cell layer (ATAC: A3, RNA: R4) and choroid plexus (ATAC: A5, RNA: R6) showing clear region-specific signals and high spatial consistency. **d** UMAP visualization of spatial RNA clusters. **e**,**g** Spatial expression patterns of AGAP12P (**e**) and SOX2 (**g**) genes, with color intensity indicating expression levels. **f**,**h** UMAP visualization of AGAP12P (**f**) and SOX2 (**h)** gene expression. **i**,**j** Spatially resolved chromatin loops anchored at AGAP12P (**i**) and SOX2 (**j**) gene TSS regions, obtained from predicted meta-spot Hi-C. k,l Super-resolution spatial Hi-C predictions at the AGAP12P (**k**) and SOX2 (**l**) anchored loops.

We used a spatial ATAC-RNA co-sequencing dataset from an adult male hippocampus^61^ (Fig. 6a) for illustration. Within this dataset, we identified two key regions with clear spatial patterns: the granular layer (ATAC cluster A3, RNA cluster R4) and the choroid plexus (ATAC cluster A5, RNA cluster R6) (Fig. 6b–d). A major challenge in spatial ATAC-seq is its relatively low sequencing depth, which falls below the input requirement of Hi-Compass. To overcome this limitation, we developed a spatial signal integration strategy similar to the meta-cell approach: merging ATAC-seq data from adjacent areas into “meta spots” to enhance local signal intensity. We then applied Hi-Compass to predict Hi-C for each meta spot, generating spatially resolved chromatin interaction maps for the human hippocampus.

Analysis of the prediction results showed distinct chromatin conformations in the granule cell layer and choroid plexus compared to other hippocampal regions (Supplementary Fig. 21a, b). To illustrate Hi-Compass’s ability to capture spatially distinct chromatin interactions, we examined two genes with opposing spatial expression patterns: AGAP12P, which showed elevated expression in the granule cell layer and choroid plexus (Fig. 6e, f), and SOX2, a well-characterized transcription factor essential for neural stem cell maintenance and neurogenesis, which displayed a complementary pattern with depletion in these regions (Fig. 6g, h). Spatially resolved chromatin loop analysis revealed that the promoters of these genes were anchored in region-specific chromatin loops that matched their respective expression domains (Fig. 6i, j**;** Supplementary Fig. 21c, d). These results demonstrate that Hi-Compass can identify biologically relevant spatial heterogeneity in chromatin organization through analyzing spatially resolved ATAC-seq data.

To enhance spatial resolution and more intuitively demonstrate these differential interactions, we employed a “super-resolution” computational method. While the initial meta-spot analysis divided the tissue into non-overlapping 5×5 regions (100 meta-spots in total), the super-resolution method instead applied a 5×5 sliding window with a step size of one spot across the original spatial positions. This strategy generates a Hi-C prediction for each original spot by integrating signals from its surrounding neighborhood, thereby preserving the native spatial resolution of the original spatial ATAC-seq data while maintaining sufficient sequencing depth for robust prediction (Fig. 6k, l).

These results validate the feasibility of computationally predicting spatially resolved 3D chromatin structures from spatial epigenomic data, establishing a computational framework for investigating spatial gene regulation in complex tissues. Although current spatial ATAC-seq datasets are limited, ongoing advances in spatial epigenomic sequencing technologies will expand the applicability of Hi-Compass to broader tissue types and pathological states. This will provide a valuable tool for analyzing the relationship between chromatin structure and gene regulation in spatially resolved contexts.

## Discussion

In this paper, we developed Hi-Compass, a generalizable computational framework for predicting 3D chromatin architecture from epigenomic data. Our approach tackles several critical limitations of existing methods by requiring only ATAC-seq signal as experimental input, implementing a meta-cell strategy to overcome single-cell data sparsity, and incorporating a depth-aware module that dynamically adjusts to varying sequencing depths. These designs collectively enable Hi-Compass to generate near-optimal, cell-type-specific Hi-C predictions across diverse biological contexts.

The superior performance of Hi-Compass compared to previous methods is evident in both quantitative metrics and visual inspections of predicted Hi-C matrices. While existing approaches have made important contributions, they typically depend on multiple input modalities and struggle with the inherent data sparsity when generalized to unseen samples and single-cell data. Hi-Compass overcomes these challenges, and effectively captures both local and global chromatin interaction patterns even from limited data inputs. The high correlation between our predictions and experimentally measured Hi-C data, particularly in the identification of TADs and chromatin loops, validates the biological relevance of our predictions.

Besides basic prediction tasks over bulk-level data, Hi-Compass demonstrates remarkable versatility across different scales of biological organizations. At the single-cell level, our meta-cell strategy successfully reconstructs cell-type-specific chromatin interactions from scATAC-seq data, as evidenced by the clear clustering patterns observed in dimensionality reduction analyses and the high concordance of predicted contacts with experimentally validated interactions. In complex tissue environments, such as PBMCs, Hi-Compass accurately captures lineage-specific chromatin conformations, enabling the identification of regulatory features that distinguish closely related cell states. Our detection of differential CXCR4 chromatin looping between B cell subtypes exemplifies the resolution and sensitivity that can be achieved through this computational approach.

The compatibility of Hi-Compass predictions with downstream multi-omics analyses further extends its utility for biological discovery. By combining predicted Hi-C structures with gene expression data in embryonic heart tissue, we identified cell-type-specific regulatory networks with functional relevance. The observation that predicted chromatin loops in fibroblasts are specifically associated with fiber-related pathways, demonstrates Hi-Compass’s ability to capture biologically relevant chromatin organization patterns that complement transcriptional analysis. This computational capability provides a valuable tool for investigating coordinated regulatory mechanisms underlying developmental processes and cell-type-specific functions. Furthermore, by connecting chromatin loops to non-coding SNP and gene promoter pairs, we revealed potential regulatory mechanisms underlying genetic associations with cardiac diseases. These SNP-to-gene links showed concordance with tissue-specific eQTL data, achieving similar consistency to experimental Hi-C approaches. This capacity to bridge genetic association studies with mechanistic understanding provides a valuable framework for interpreting non-coding variants in complex diseases and offers new opportunities for developing targeted therapeutic strategies.

The application of Hi-Compass to spatial epigenomic data represents a new attempt. By integrating spatial ATAC-seq signals into meta-spots, we demonstrate the feasibility of computationally predicting spatially-resolved 3D chromatin structures within intact tissues. The successful identification of region-specific chromatin interactions in hippocampus, with clear correspondence to local gene expression patterns, demonstrates the method’s capability to capture spatial heterogeneity in chromatin organization. This computational bridge between spatial epigenomics and 3D genome architecture fills a critical gap in spatial omics research, where experimental methods to directly measure chromatin conformation in a spatially resolved manner remain technically challenging.

Beyond human cells, we demonstrate that Hi‑Compass can be effectively adapted to mouse through lightweight fine‑tuning, achieving prediction accuracy comparable to that in human samples for bulk and resolving subtype‑specific chromatin dynamics in single-cell T-cell populations. These findings establish Hi‑Compass as a versatile tool for studying chromatin topology across species and support its potential in evolutionary studies of genome regulation.

Despite these advances, Hi-Compass has certain limitations that should be acknowledged. First, the resolution of our predictions (10 kb) is constrained by the available training data and computational resources. Although this resolution is sufficient for identifying most functional chromatin interactions, some fine-scale regulatory features might be missed. Second, while Hi-Compass was initially trained on human cell lines and successfully transferred to mouse data through fine-tuning, its applicability to more evolutionarily distant species remains to be systematically investigated. Third, while Hi-Compass can predict chromatin interactions from sparse data, there remains a minimum depth threshold (approximately 2e5 reads) below which prediction quality degrades, potentially limiting its applications to extremely rare cell populations or severely low-input samples.

In conclusion, Hi-Compass provides a powerful computational framework for exploring 3D genome architecture across biological scales and systems. By requiring only ATAC-seq data for high-quality chromatin interaction prediction, it dramatically expands the range of biological questions that can be addressed through 3D genomic analysis. From individual cell types to complex tissues and spatial contexts, Hi-Compass enables researchers to gain insights into the spatial organization of the genome and its role in gene regulation, offering new perspectives on cellular identity and function in health and disease.

## Methods

### Hi-C data processing

We collected high-quality in-situ Hi-C datasets of 14 human cell types and 4 mouse cell types from ENCODE^62^, 4DN^63^, and GEO^64^ (Supplementary Data 1), respectively. To ensure consistency and minimize technical variability in Hi-C data preprocessing, we implemented a standardized computational pipeline to process all data from raw FASTQ files. We aligned reads against hg38 reference genome by using BWA^65^ (v0.7.19) to obtain the bam files, then converted to pairs files using pairtools^66^ (v1.1.3), and created cool files using Cooler^67^ (v0.10.3) at 10 kb resolution. To facilitate downstream analysis compatibility and optimize the matrices for deep learning applications, we performed contrast stretching normalization by scaling each chromosome’s Hi-C matrix values relative to its 98th percentile, thereby standardizing all interaction frequencies to a uniform range of 0 to100.

### Bulk ATAC-seq data processing

We collected ATAC-seq datasets corresponding to the Hi-C samples and processed them through the following pipeline: First, FASTQ files were aligned to the reference genome using BWA to generate BAM files. These BAM files were then converted to bedGraph format using the genomecov command in bedtools^68^ (v2.28.0), followed by transformation into bigWig format using bedGraphToBigWig from the UCSC Genome Browser^69^ toolkit (v4) for subsequent downstream analyses. In addition to processing the original bulk ATAC-seq data, we developed a systematic downsampling strategy to enable Hi-Compass to accommodate ATAC-seq data of varying sequencing depths. Specifically, we randomly sampled reads from the original SAM files at different proportions to generate simulated datasets with sequencing depths ranging from 2e4 to 2e7 reads, with increments of 2e4 reads.

### CTCF ChIP-seq data processing

We collected CTCF ChIP-seq data from 165 human samples and 42 mouse samples from Cistrome DB, respectively. We downloaded the peak files in BED format. To construct generalized human and mouse CTCF binding profile, we integrated all peak files using the Multiinter function from bedtools, generating a consolidated bedGraph file where the value at each genomic position represents the frequency of CTCF binding events across samples. These raw counts were then normalized by the total sample number (*n* = 165 for human and 42 for mouse) to derive a binding probability score ranging from 0 to 1. Finally, we converted the normalized bedGraph files to bigWig format using bedGraphToBigWig for model input.

### DNA sequence processing

We obtained the human (hg38) and mouse (mm10) reference genome sequence from the UCSC Genome Browser database. The original FASTA file includes four types of nucleotides and ‘N’ for unknown type. During encoding, we retained the ‘N’ category and encoded it as the fifth “nucleotide”. After encoding, each nucleotide is represented as a five-channel one-hot vector representing ‘ATCGN’, respectively.

### Model architecture

Hi-Compass employs a CNN and Attention-based encoder-decoder framework. The model incorporates three parallel encoders that process DNA sequence, ATAC-seq signal, and generalized CTCF ChIP-seq signal, respectively.

The DNA sequence encoder (DNAEncoder) utilizes a series of one-dimensional convolutions and residual modules to extract DNA sequence features. It first transforms the 5-channel one-hot encoded DNA sequence into 32-channel features through a convolutional layer, followed by 12 residual convolutional modules that gradually increase feature channels from 32 to 256 while reducing sequence length. Each residual module includes batch normalization and ReLU activation functions, with residual connections preserving the original information. Additionally, the encoder implements a feature weighting mechanism that applies learnable parameters to weight different nucleotide features.

The ATAC-seq signal encoder (ATACDepthEncoder) has a similar structure but incorporates a depth adaptation module, with the entire encoder accepting both the ATAC-seq signal information and an additional scalar value representing the total read depth. The depth adaptation module first maps the total read depth to predefined depth range intervals, then generates depth feature vectors through an embedding layer, which are multiplied with the ATAC signal, enabling the model to dynamically adjust feature extraction based on the sequencing depth of input data.

The CTCF signal encoder (CTCFEncoder) shares a similar structure with the ATAC encoder but without the depth adaptation module, specifically designed to process generalized CTCF binding site signals.

The outputs from the three encoders are integrated along the feature dimension, with a module supporting attention-based fusion methods to combine information from different feature sources into a unified representation. The fused features are then fed into a Transformer module consisting of 8 self-attention layers, each using 8 attention heads and relative positional encoding to capture positional information.

The decoder consists of five two-dimensional residual convolutional layers with different dilation rates (2,4,8,16,32). These dilated convolutions are designed to ensure that each output pixel’s receptive field can cover a sufficiently large input region, reinforcing the modeling of interactions between different regions. Finally, the decoder transforms the 256×256 single-channel matrix through an adaptive average pooling layer into a 209×209 size, representing the Hi-C contact map of a 2 Mb (2,097,152 bp) range at 10 kb resolution. After outputting the contact map, the model further inputs the matrix into a discriminator composed of ResNet and fully connected layers. This discriminator attempts to predict and output the cell type based on the cell type specificity exhibited by the predicted Hi-C contact map. The model is constructed using Pytorch (v2.1.0).

### Loss function

To assess the quality of predicted Hi-C matrices within a 2 Mb genomic interval, we employed two complementary loss metrics: MSE and Information-Weighted Structural Similarity (IW-SSIM).

For MSE, the loss function ${\rm{Los}}{{\rm{s}}}_{{\rm{MSE}}}$ is formulated as:

1 $${\rm{Los}}{{\rm{s}}}_{{\rm{MSE}}}={mean}\left({\left({M}_{g}-{M}_{p}\right)}^{2}\right)$$

For IWSSIM, it is an advanced perceptual quality assessment approach derived from traditional Structural Similarity (SSIM) that more accurately captures the structural characteristics of Hi-C contact matrices. The traditional SSIM compares two images based on luminance, contrast, and structure. For two image patches x and y, SSIM is calculated as:

2 $${\rm{SSIM}}\left(x,y\right)=l{\left(x,y\right)}^{\alpha } \cdot c{\left(x,y\right)}^{\beta } \cdot s{\left(x,y\right)}^{\gamma }$$

where $l\left(x,y\right)$, $c\left(x,y\right)$, and $s\left(x,y\right)$ represent luminance, contrast, and structural similarity, respectively:

3 $$l\left(x,y\right)=\frac{2{\mu }_{x} \cdot {\mu }_{y}}{{{\mu }_{x}}^{2}+{{\mu }_{y}}^{2}}$$

4 $$c\left(x,y\right)=\frac{2{\sigma }_{x} \cdot {\sigma }_{y}}{{{\sigma }_{x}}^{2}+{{\sigma }_{y}}^{2}}$$

5 $$s\left(x,y\right)=\frac{2{\sigma }_{{xy}}}{{\sigma }_{x} \cdot {\sigma }_{y}}$$

where ${\mu }_{x}$, ${\mu }_{y}$ are means of $x$ and $y$, ${\sigma }_{x}$ and ${\sigma }_{y}$ are their standard deviations, and ${\sigma }_{{xy}}$ is their covariance. Typically, $\alpha=\beta=\gamma=1$, simplifying to:

6 $${\rm{SSIM}}\left(x,y\right)=l\left(x,y\right) \cdot c\left(x,y\right) \cdot s\left(x,y\right)$$

Conventional SSIM implementation uses a sliding window to segment an image into spatially distributed patches, calculates SSIM values for each patch, and averages these values to obtain the final SSIM score.

Multi-scale SSIM (MS-SSIM) extends this concept by incorporating structural similarity calculations at multiple spatial scales. For the $j$ th scale and $i$ th spatial location, ${\rm{SSI}}{{\rm{M}}}_{{\rm{j}}}$ is computed as:

7 $${\rm{MS}}-{\rm{SSI}}{{\rm{M}}}_{{\rm{j}}}\left(x,y\right)=\left\{\begin{array}{c}\frac{1}{M}\mathop{\sum }\limits_{i}c\left({x}_{{ji}},{y}_{{ji}}\right) \cdot s\left({x}_{{ji}},{y}_{{ji}}\right),j=1,\ldots,M-1\\ \frac{1}{M}\mathop{\sum }\limits_{i}l\left({x}_{{ji}},{y}_{{ji}}\right) \cdot c\left({x}_{{ji}},{y}_{{ji}}\right) \cdot s\left({x}_{{ji}},{y}_{{ji}}\right),j=M\end{array}\right.$$

The final MS-SSIM is defined as:

8 $${\rm{MS}}-{\rm{SSIM}}\left(x,y\right)=\mathop{\prod }\limits_{j=1}^{M}{\left({\rm{SSI}}{{\rm{M}}}_{{\rm{j}}}\right)}^{{\beta }_{j}}$$

where ${\beta }_{j}$ are hyperparameters for scale weighting.

The key innovation of IW-SSIM lies in its information content-based weighting strategy. This method, grounded in information theory principles of visual perception, gives higher importance to image regions with richer information content during similarity assessment.

Information content estimation is based on the Gaussian Scale Mixture (GSM) model, which represents local image structures as a product of a Gaussian vector and a positive scalar:

9 $$C=s \cdot U$$

where $U$ is a K-dimensional zero-mean Gaussian vector with covariance matrix ${C}_{U}$, and $s$ is a mixing multiplier representing local intensity. Through the GSM model, each local region’s information content $w$ is calculated as:

10 $$w=\frac{1}{2}{\log }_{2}\left\{\mathop{\prod }\limits_{k=1}^{K}\left[1+\frac{{\sigma }_{v}^{2}}{{\sigma }_{n}^{2}}+\left(\frac{{\sigma }_{v}^{2}}{{\sigma }_{n}^{4}}+\frac{{1+g}^{2}}{{\sigma }_{n}^{2}}\right){s}^{2}{\lambda }_{k}\right]\right\}$$

where ${\sigma }_{v}^{2}$ and ${\sigma }_{n}^{2}$ represent visual channel and noise variances respectively, $g$ is the gain factor, ${\lambda }_{k}$ is the kth eigenvalue of the covariance matrix ${C}_{U}$, and ${s}^{2}$ is the estimated mixing multiplier for the local region.

In IW-SSIM, each SSIM calculation at spatial position $i$ and scale $j$ is weighted by its corresponding information content:

11 $${\rm{IWSSI}}{{\rm{M}}}_{{\rm{j}}}\left(x,y\right)=\frac{\mathop{\sum }\limits_{i}{w}_{{ji}} \cdot {\rm{SSI}}{{\rm{M}}}_{{\rm{j}}}\left({x}_{i},{y}_{i}\right)}{\sum {w}_{{ji}}}$$

The final IW-SSIM is computed as:

12 $${\rm{IWSSIM}}\left(x,y\right)=\mathop{\prod }\limits_{j=1}^{M}{\left({\rm{IWSSI}}{{\rm{M}}}_{{\rm{j}}}\right)}^{{\beta }_{j}}$$

For implementation in Hi-Compass, given ground truth Hi-C matrix ${M}_{g}$ and predicted Hi-C matrix ${M}_{p}$, we define the IW-SSIM loss function as:

13 $${\rm{Los}}{{\rm{s}}}_{{\rm{IWSSIM}}}=1-{\rm{IWSSIM}}\left({M}_{g},{M}_{p}\right)$$

We adopted the five-scale decomposition approach from the original paper, with scale weights ${{\rm{\beta }}}_{1}=0.0448,{{\rm{\beta }}}_{2}=0.2856,\,{{\rm{\beta }}}_{3}=0.3001,{{\rm{\beta }}}_{4}=0.2363,{{\rm{\beta }}}_{5}=0.1333$.

In addition to the two aforementioned loss functions for matrix prediction, a cross-entropy loss function is also applied between the predicted cell line types from the Hi-Compass discriminator and the actual cell line type of each fragment. Given C cell line types in the training set, where ${g}_{c}$ represents the ground truth label for the sample belonging to cell line type $c$, and ${p}_{c}$ denotes the probability predicted by the discriminator that this sample belongs to cell line type $c$, the ${\rm{Los}}{{\rm{s}}}_{{\rm{CE}}}$ can be formulated as:

14 $${\rm{Los}}{{\rm{s}}}_{{\rm{CE}}}=-\mathop{\sum }\limits_{c=1}^{C}{g}_{c}\log \left({p}_{c}\right)$$

The final loss function can be described as:

15 $${\rm{Loss}}={\lambda }_{{\rm{CE}}}{\rm{Los}}{{\rm{s}}}_{{\rm{CE}}}+{\lambda }_{{\rm{MSE}}}{\rm{Los}}{{\rm{s}}}_{{\rm{MSE}}}+{\lambda }_{{\rm{IWSSIM}}}{\rm{Los}}{{\rm{s}}}_{{\rm{IWSSIM}}}$$

The Hi-Compass model trained in this paper set ${\lambda }_{{\rm{CE}}}=0.2,{\lambda }_{{\rm{MSE}}}=0.4,{\lambda }_{{\rm{IWSSIM}}}=0.4$.

### Model training strategy

During the training process of human dataset, we used a batch size of 4 and the Adam optimizer with an initial learning rate of 0.002, implementing a cosine annealing scheduler with warmup. To prevent gradient explosion in the early training phase, we adopted a staged loss function strategy: the model was initially trained with MSE loss only, and SSIM and cross-entropy losses were introduced for multi-task training after the MSE loss decreased below 1.5. To enhance the model’s generalization ability and robustness, we employed various data augmentation techniques. For all three types of sequence data inputs, we randomly applied Gaussian noise to ATAC-seq sequence data, swapped small sequence segments, and slightly shifted sequence positions, each with a probability of 0.3.

Our constant input data consisted of the DNA sequences and generalized CTCF ChIP-seq signals. The variable training input dataset included ATAC-seq data from 6 cell lines (IMR90, GM12878, K562, CT27 EVT, Huh1 and SNU449) at five different sequencing depths (2e5, 5e5, 1e6, 2e6, and bulk). In terms of chromosomes, all chromosomes except Chr2 (used as the validation set) and Chr11 (used as the test set) were used for training. The ATAC-seq signals for the validation and test set chromosomes were derived from depths of 8e5, 3e6, and bulk. The Hi-Compass model presented in this paper was trained for ten epochs under these settings. The total training time was approximately 120 hours on a computing cluster equipped with four NVIDIA RTX 3090 GPUs.

### Cross-species fine-tuning for mouse genome

To extend Hi-Compass to the mouse genome, we employed a transfer learning strategy. The model was initialized with pre-trained human weights and fine-tuned on mouse Hi-C data. ATAC-seq data from two mouse cell types (TT2 and mESC), along with the generalized mouse CTCF profile and mouse reference genome sequence, were used for training. Chromosomes 15−17 served as the validation set and chromosomes 18–19 as the test set. The same multi-depth sampling strategy as human model training was applied, with ATAC-seq data at five different sequencing depths (2e5, 5e5, 1e6, 2e6, and bulk). The fine-tuning was performed for 3 epochs with a reduced learning rate of 0.001. Other training settings remained the same as the human model training.

### Hi-C prediction and integration

The prediction unit of Hi-Compass is a Hi-C fragment with a size of 2 Mb and a resolution of 10 kb (actual matrix dimension of 209×209). To generate genome-wide prediction results, we employ a sliding window prediction pipeline. Specifically, we slide the prediction window along chromosomes with a fixed step size, generating prediction results for each window. The step size can be specified by users according to their task requirements for the maximum number of complete diagonals. For example, when the step size is set to 159, the final number of complete diagonals obtained is 50. A smaller step size means more complete diagonals, and also implies slower inference speed. In practice, generating a genome-wide Hi-C prediction for a human sample with 50 complete diagonals (step size = 159) takes approximately 8 min on a personal computer equipped with an NVIDIA RTX 4070 or a server with RTX 3090, including data loading, prediction, and cool file assembly. For the mouse genome under the same settings, the process takes approximately 6 minutes due to the smaller genome size. The integrated chromosome-level prediction results are saved in.cool file format using the create_cooler function from Cooler.

### Insulation score and correlation

Insulation score is an important indicator for evaluating chromatin topological structure features, suitable for quantifying the strength of TAD boundaries. It essentially calculates the average chromatin contact frequency within a sliding window at each position. When calculating the insulation score for Hi-Compass prediction results, since cool files have already been generated, we directly use the insulation function from cooltools (v0.7.1) to calculate the overall insulation score.

When evaluating model performance, we calculated the Pearson correlation coefficient between the insulation scores of predicted Hi-C and experimentally measured Hi-C. For both, we used the insulation function from cooltools with identical parameters (window size = 20 bins) to obtain insulation scores. Since insulation scores at 10 kb resolution are relatively long, we segmented the insulation scores of each chromosome into shorter sequences suitable for correlation calculation, using a step size of 200 bins. Finally, we quantified and compared the overall performance of Hi-Compass under different input data conditions through the distribution statistics of these fragment correlations.

### TAD boundary calling and recall assessment

TAD boundaries were identified from insulation score profiles using cooltools with four window sizes (5, 10, 15, and 25 bins). For each genomic bin, cooltools returns a boolean value indicating whether it is a TAD boundary at each window size. A genomic position was classified as a TAD boundary if it was identified as a boundary at any of the four window sizes. This criterion was applied consistently to both experimental Hi-C and Hi-Compass-predicted Hi-C data. To evaluate prediction performance, we calculated the recall rate as the proportion of experimentally identified TAD boundaries that were also detected in the predicted Hi-C data.

### Performance comparison with previous methods

To evaluate the performance of Hi-Compass, we conducted a systematic comparison with five existing methods (Akita, C.Origami, Epiphany, ChromaFold and AlphaGenome). To ensure the fairness of the evaluation, we used the same chromosomal regions for testing and the same evaluation metrics for all methods. The test regions included sequence segments from different chromosomes, covering different types of chromatin regions across the genome. For each method, we used their respective published pre-trained models for prediction, with input data being the default sequence types required by each method. Since different methods operate at different native resolutions (Akita and AlphaGenome at 2 kb; C.Origami, Epiphany, ChromaFold, and Hi-Compass at 10 kb), we calculated insulation scores at each method’s native resolution and compared correlations with experimental Hi-C data processed at matching resolutions.

For the evaluation metric, we uniformly used the Pearson correlation and Spearman correlation of insulation score. Since the different models used drastically different gold standard data formats during training, and none of the methods except Hi-Compass can generate complete cool files, we consistently calculated the insulation score directly on each model’s predicted segments against the corresponding gold standard segments. Specifically, we applied AdaptiveAvgPool2d from the PyTorch library with a window size of 20, then selected the 22nd diagonal, which represents the insulation score of the segment with a window size of 20 while ignoring the first two diagonals. We then calculated the Pearson correlation coefficient of these segment insulation scores between the predicted results and the experimental values.

To visually compare the performance differences of different methods, we used box plots to show the correlation distribution of all methods across all tested windows. At the same time, we also selected typical genomic regions for side-by-side visual comparison of prediction results from different methods.

### Single-cell ATAC-seq and Multiome data processing

We collected and processed various single-cell ATAC-seq (scATAC-seq) and Multiome datasets. For human scATAC-seq, we used seven different cell lines (IMR90, GM12878, K562, HCT116, A549, A673, H9_hESC) from the same laboratory, as well as the PBMC3k dataset provided by 10X Genomics. For mouse scATAC-seq, we used splenic CD4^+^ T cell data from a published study. For multiome data, we utilized the 105-day female human embryonic heart tissue data from ENCODE^62^.

All single-cell sequencing data were generated on the 10x Genomics platform. The scATAC-seq data were processed using Cell Ranger ATAC (v1.2.0), while Multiome data were processed with Cell Ranger ARC (v2.0.2). The resulting fragments files were imported into the R language environment and converted into Signac objects using the Signac (v1.6.0) and Seurat (v5.1.0) packages. The data preprocessing workflow included normalization using RunTFIDF in Signac, feature selection with FindTopFeatures, and dimension reduction using RunSVD based on the LSI method.

For cell type annotation, we adopted different strategies for different data types: For the seven cell lines’ scATAC-seq data, we processed each dataset separately and then merged them into a single entity using Signac’s merge function, directly using the original cell line names as cell type labels. For the PBMC scATAC-seq data, we downloaded an expert-annotated 10X PBMC scRNA-seq dataset as a reference, identified anchors using FindTransferAnchors in Signac, and then transferred cell type annotations from scRNA-seq to scATAC-seq cells using TransferData. For the mouse spleen T cells, we use the gene activity score of Foxp3 to directly identify conventional T cells and regulatory T cells. For the embryonic heart Multiome data, we annotated cell types based on the expression patterns of known marker genes and chromatin accessibility features, combined with reference information from existing literature.

### Meta-cell strategy and meta-cell Hi-C prediction

The core concept of the meta-cell strategy is to integrate ATAC signals from multiple single cells of the same cell states to build input data that reaches the depth threshold required for prediction. In implementation, we used k-means clustering within each annotated cell type to construct meta-cells. The number of clusters k was determined based on the expected number of cells per meta-cell: if m cells were expected in each meta-cell and the total number of cells was n, then k was set as the nearest integer to $\frac{{\rm{n}}}{{\rm{m}}}$.

After determining the cell barcodes corresponding to each meta-cell, we used the sinto (v0.10.1) software to extract the corresponding meta-cell bam files from the original scATAC-seq bam files. Subsequently, we converted the bam files to bedGraph format using the genomecov command in bedtools, and then generated bigWig (bw) files using UCSC’s bedGraphToBigWig tool. These bw files served directly as ATAC-seq input for Hi-Compass to predict Hi-C contact maps for the corresponding meta-cells. The ATAC-seq signal depth for each meta-cell was obtained by counting the number of reads in the corresponding bam file.

To evaluate the quality and cell type specificity of the prediction results, we performed feature extraction and dimensionality reduction analysis on each predicted meta-cell Hi-C. Specifically, we extracted the data from the first 50 diagonals of each predicted Hi-C matrix and flattened it into a one-dimensional vector as the feature representation of that meta-cell. These features were then dimensionally reduced through PCA and visualized in two dimensions using the UMAP algorithm to assess clustering patterns and separation degrees between meta-cells of different cell types.

### Loop calling

For chromatin loops identification in Hi-Compass predicted Hi-C data, we employed Mustache (v1.3.3). When comparing these results with gold standard data, we consistently applied Mustache to the experimental Hi-C datasets to ensure methodological consistency. For aggregate peak analysis (APA), we utilized HICCUPS in Juicebox (v2.3.5) as it is optimized for this specific analytical approach. All analyses were performed using default parameters unless otherwise specified.

### APA procedures

We identified chromatin loops in the experimental Hi-C data using HICCUPS and extracted all loops with genomic distances less than 50 bins between bin1 and bin2. For each identified loop, we extracted 11×11 bin submatrices centered on the loop anchors from both the experimental Hi-C and Hi-Compass predicted Hi-C matrices. By averaging these submatrices across all loops, we generated aggregate peak analysis (APA) matrices for both the experimental and predicted data, enabling quantitative assessment of prediction accuracy against experimental observations.

### Validation against orthogonal protein-mediated interaction data

To validate Hi-Compass predictions against orthogonal protein-mediated interaction data, we performed APA using two external loop datasets in GM12878: cohesin (Smc1a) HiChIP loops and RAD21 ChIA-PET loops. Both datasets were converted to hg38 coordinates and mapped to 10 kb resolution. APA was performed following the same procedure described above.

### Differential loop detection

In addition to identifying loops in individual Hi-C datasets, we performed differential loop analysis between distinct Hi-C datasets using the diff_mustache function of Mustache. To ensure high confidence in the differential loops identified, we implemented a dual-criteria filtering approach: differential loops were required to pass the FDR statistical significance threshold (FDR < 0.01) in diff_mustache, and additionally, the contact frequency at the loop location in the enriched Hi-C dataset had to be at least 1.5-fold higher than the corresponding location in the comparison dataset. This stringent filtering strategy ensured that the identified differential loops represented biologically meaningful chromatin interaction changes.

### Statistical analysis of cell-type-specific loop predictions

To quantitatively assess whether Hi-Compass predictions captured cell-type-specific chromatin loops, we performed Mann-Whitney U tests comparing predicted contact frequencies at loop peaks between the target cell type and other cell types. For each experimentally identified cell-type-specific loop, we extracted the predicted contact values at the loop peak across all meta-cells and compared the distribution between meta-cells of the corresponding cell type versus meta-cells of other cell types. Effect size was calculated as the rank-biserial correlation coefficient (r), which ranges from -1 to 1, with values > 0.6 indicating a large effect.

### Loop annotation and loop-associated gene identification

We defined promoter regions using transcription start sites (TSSs) from the GENCODE v38 reference annotation database. For all chromatin loops identified by the methods described above, we employed the pybedtools (v0.12.0) Python package to pair loop anchor regions and gene TSS regions. Specifically, genes whose TSS regions overlapped with either anchor of a chromatin loop were classified as “loop-related genes”. By annotating differential loops in each cell type’s Hi-C data relative to the average matrix of all other Hi-C datasets, we identified cell-type-specific loop-related genes.

### SNP-to-gene linking strategy

To identify potential regulatory relationships between genetic variants and target genes, we developed a computational pipeline that leverages Hi-Compass-predicted chromatin loops to connect non-coding regulatory regions with gene promoters. For each predicted Hi-C matrix from embryonic heart tissue data, we identified significant chromatin interactions using Mustache with default parameters. Loop anchors were then annotated to identify those overlapping with heart disease-associated SNPs from the GWAS Catalog at one end and gene promoter regions at the other end.

A SNP-to-gene link was established when one anchor of a chromatin loop overlapped with a GWAS SNP location and the corresponding partner anchor overlapped with a gene promoter region. To validate the biological relevance of these computationally identified SNP-to-gene links, we compared them with tissue-specific expression quantitative trait loci (eQTL) from the GTEx project (v10 release). For each predicted SNP-to-gene pair, we checked whether the same SNP (or any SNP in high linkage disequilibrium, r² > 0.8) was reported as an eQTL for the linked gene in heart-related tissues (Artery Coronary, Left Ventricle, and Atrial Appendage). We calculated the overlap percentage between our predicted links and experimentally determined eQTLs to assess the accuracy of our approach.

To evaluate the disease specificity of chromatin interactions identified from Hi-Compass-predicted Hi-C, we performed enrichment analysis of GWAS variants from the GWAS Catalog (v1.0.2, accessed March 2025). For each disease trait, we calculated the enrichment ratio as the proportion of trait-associated SNPs that could be functionally annotated through predicted chromatin loops. A SNP was considered loop-annotated if it overlapped with one anchor of a predicted loop while the paired anchor overlapped with a gene promoter region (TSS ± 2 kb, based on GENCODE v38 annotations). For comparison, we also calculated the proportion of SNPs overlapping with ATAC-seq peaks. Enrichment ratios were compared between cardiac-related traits and non-cardiac traits to assess disease specificity.

### Meta-spot integration and meta-spot Hi-C prediction for spatial ATAC-seq

We obtained spatial ATAC-seq data and corresponding spatial RNA-seq data from the GEO database, downloading fragment files and matrix files, respectively. We performed dimensionality reduction and clustering analyses on spatial ATAC-seq and spatial RNA-seq data using the Signac and Seurat packages, respectively. To generate meta-spots for Hi-Compass Hi-C prediction, we developed two integration approaches: (1) a low-resolution method that uniformly divided the original 50×50 spatial positions into 100 meta-spots of 5×5 size, enabling rapid prediction; and (2) a high-resolution method that applied a 5×5 sliding window with a step size of one spot across the original spatial positions, generating meta-spot ATAC-seq data at the same resolution as the original spatial map for enhanced visualization and prediction.

### Reporting summary

Further information on research design is available in the Nature Portfolio Reporting Summary linked to this article.

## Supplementary information

Supplementary Information

Description of Additional Supplementary Files

Supplementary Data 1

Supplementary Data 2

Supplementary Data 3

Supplementary Data 4

Reporting Summary

Transparent Peer Review file

## Data Availability

The Hi-C, CTCF ChIP–seq and ATAC–seq datasets used in the study were all public data from the ENCODE, 4DN and GEO database, with the detailed information listed in the Supplementary Data 1-3. Cohesin HiChIP loop calls in GM12878 were obtained from Mumbach et al.^43^ and RAD21 ChIA-PET loops from Grubert et al.^44^ The single-cell ATAC-seq, Multiome and spatial ATAC-seq data were obtained from 10X Genomics (https://www.10xgenomics.com/datasets/), ENCODE and GEO database, with the detailed information listed in the Supplementary Data 4. GWAS SNPs data was obtained from GWAS Catalog^70^ (https://www.ebi.ac.uk/gwas/), eQTLs data from GTEx^71^ v10 (https://www.gtexportal.org/).

## Code availability

The Hi-Compass framework was implemented in the ‘hicompass’ Python package, which is available at https://github.com/EndeavourSyc/Hi-Compass. The specific version of the code associated with this publication is archived in Zenodo (10.5281/zenodo.19283061).
