# Supplementary material for: Hi-Compass: a depth-aware deep learning framework for predicting cell-type-specific 3D genome organization from single-cell to spatial resolution
Source: Nat Commun. 2026 Apr 14;17:5172. doi: 10.1038/s41467-026-71877-z (PMC13250166; doi:10.1038/s41467-026-71877-z)
Supplement: Supplementary file 1 — Supplementary Information [file 41467_2026_71877_MOESM1_ESM.pdf]

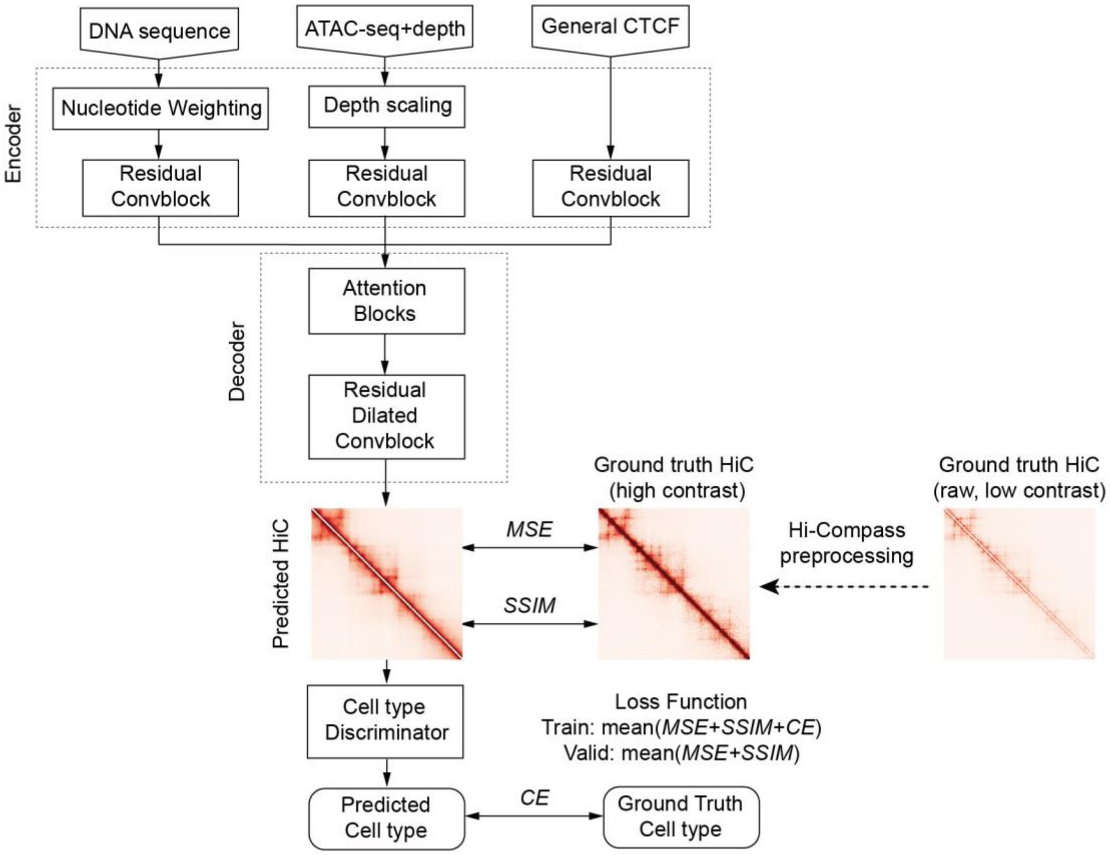

Supplementary Fig. 1 | The model architecture of Hi-Compass. The model employs three parallel convolutional networks to process different types of input data, including DNA sequence, ATAC-seq signal, and generalized CTCF binding site signal. The ATAC-seq processing branch includes a depth adaptive module. After fusion, all features are processed through a Transformer decoder to generate the final Hi-C matrix prediction through dilated convolutional block. Prior to model training, raw Hi-C matrices with low contrast are preprocessed using contrast stretching normalization to enhance the visibility of chromatin interaction features while preserving structural patterns. During model training, MSE, SSIM, and cell type discrimination cross entropy loss functions are optimized simultaneously. MSE, mean squared error. SSIM, structural similarity. CE, cross entropy.

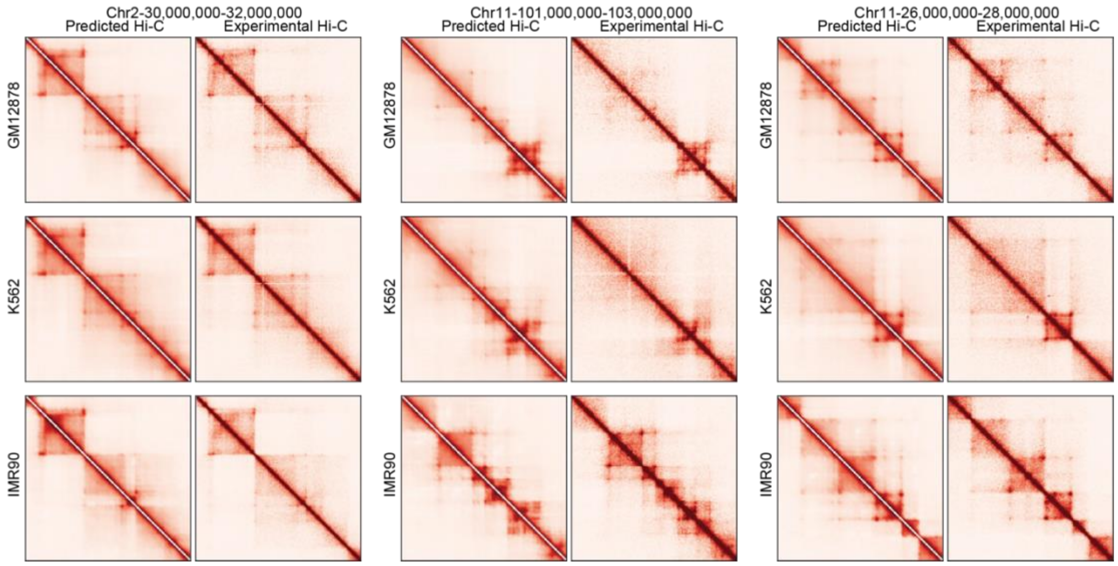

Supplementary Fig. 2 | Cross-cell-type comparison of Hi-Compass predictions at identical genomic regions. Predicted (left) and experimental (right) Hi-C contact maps for three cell lines (GM12878, K562, and IMR90) across three representative genomic regions shown in **Fig. 1b**. All maps are displayed at 10 kb resolution.

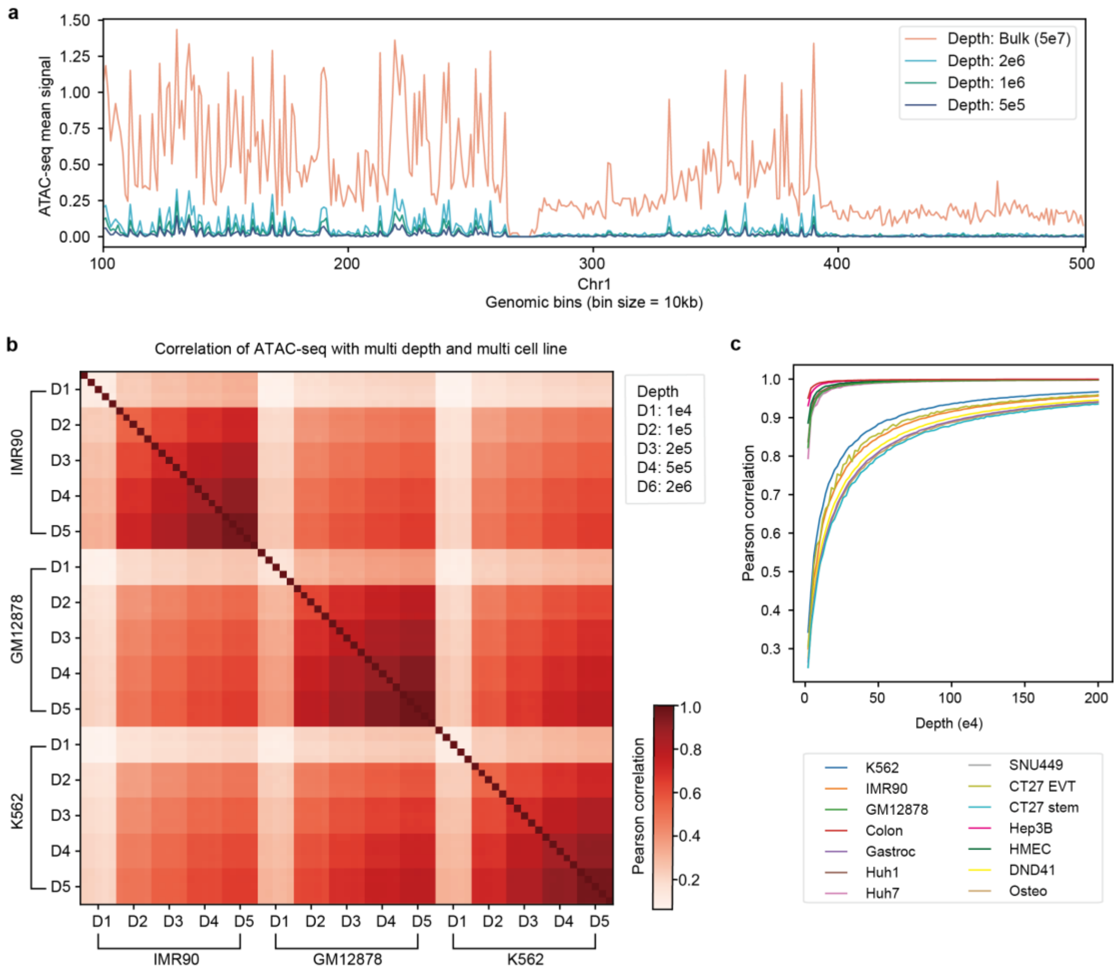

Supplementary Fig. 3 | Downsampled ATAC-seq data maintains high fidelity to original bulk signals within specific sequencing depth thresholds. a, Signal intensity profiles comparing different sequencing depth, including bulk and three downsampled data, across a 4 Mb genomic region (Chr1-1,000,000-5,000,000) in IMR90 ATAC-seq data. **b,** Pairwise Pearson correlation matrix of ATAC-seq signals among three cell lines' ATAC-seq data at five different downsampling depths. **c,** Quantitative assessment of data fidelity showing the relationship between downsampling depths and signal preservation, represented by Pearson correlation coefficients between downsampled and bulk ATAC-seq data across multiple cell lines.

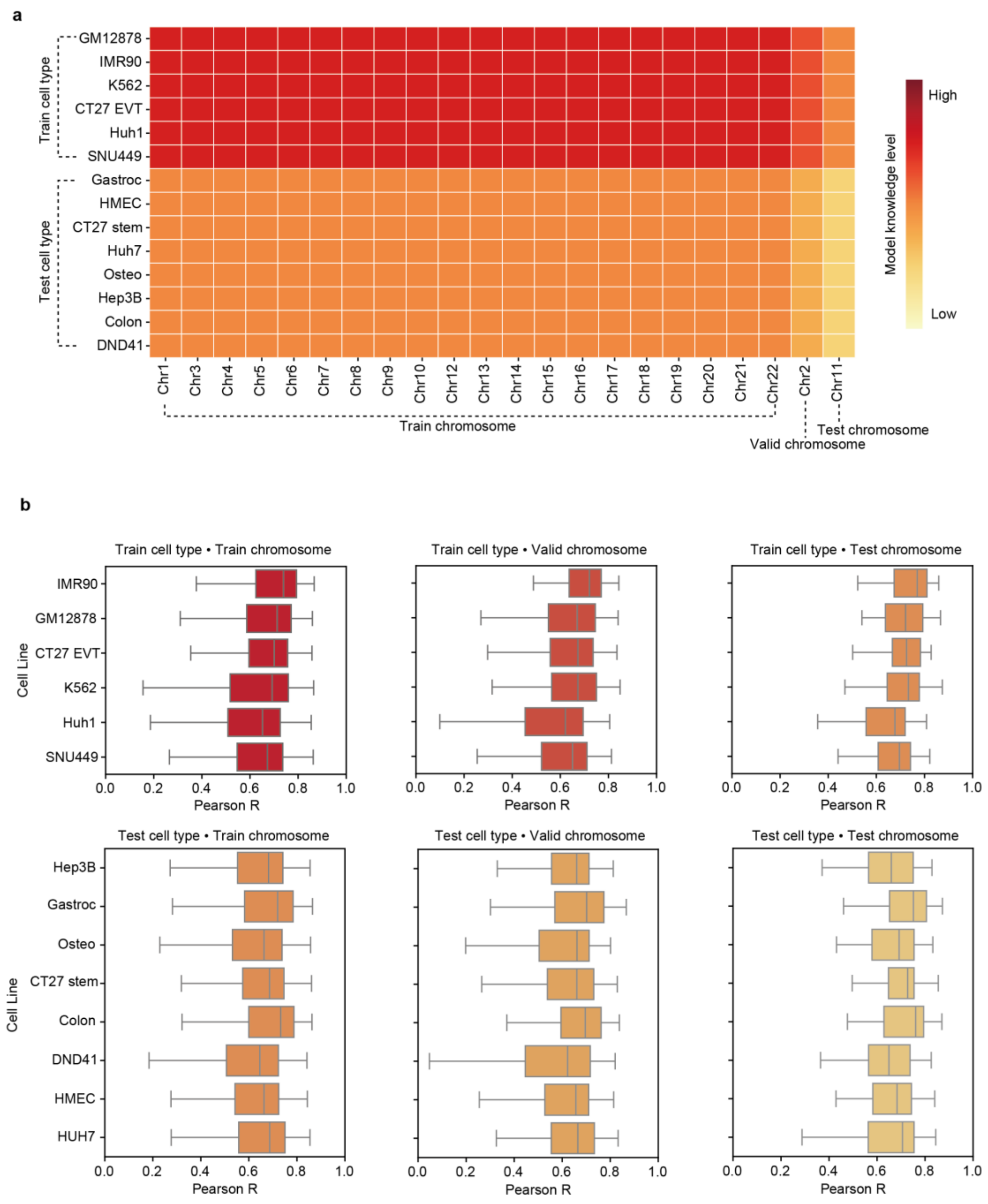

Supplementary Fig. 4 | Evaluation of Hi-Compass generalization across cell types and chromosomes in human. a, Heatmap showing model knowledge level across cell types (rows) and chromosomes (columns). Cell types are divided into training set and test set. Chromosomes are categorized into training, validation (Chr2), and test (Chr11) sets. Gastroc: Gastrocnemiu; Osteo: Osteogenesis; Colon: Transverse colon; HMEC: Human Mammary Epithelial Cells; CT27 EVT: CT27 Extravillous Trophoblast; CT27 stem: CT27 Trophoblast Stem Cells. **b**, Box plots showing insulation score correlation across different cell type and chromosome combinations. Box plot colors correspond to the model knowledge levels in panel **a**. The center line represents the median, box bounds the 25th and 75th percentiles, and whiskers extend to 1.5 times the interquartile range (n = 1,039 for training chromosomes, 113 for validation chromosome, 63 for test chromosome).

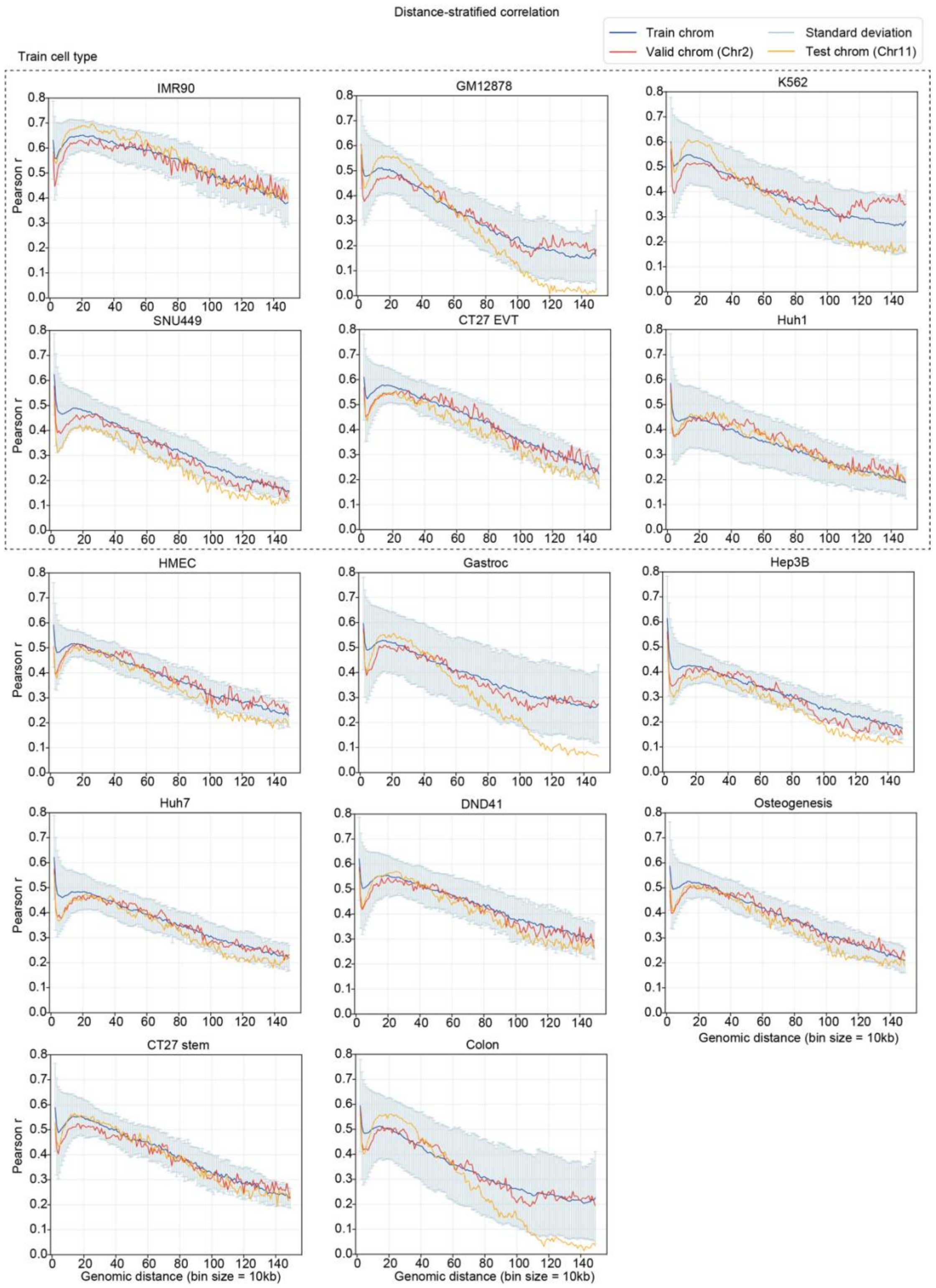

Supplementary Fig. 5 | Genome-wide distance-stratified correlation analysis of Hi-Compass predictions across multiple human cell types. Line plots display Pearson correlation coefficients across genomic separation distances (matrix diagonals) for training set chromosomes (average over multiple chromosomes), validation chromosome (Chr2), and test chromosome (Chr11). Cell types in the dashed box are used for training.

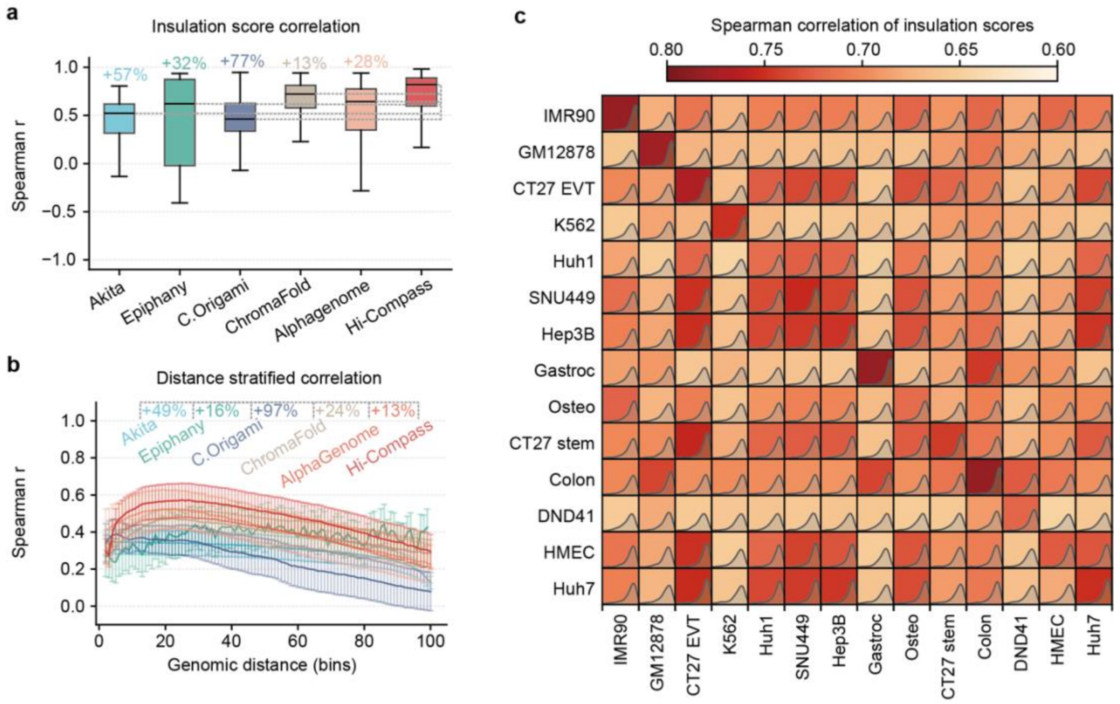

Supplementary Fig. 6 | Spearman correlation analysis of Hi-Compass prediction performance. a, Spearman correlation coefficients of insulation scores between predicted and experimental Hi-C across all cell types for each method. The center line represents the median, box bounds the 25th and 75th percentiles, and whiskers extend to 1.5 times the interquartile range (n = 7,840 for Akita, n = 2,566 for AlphaGenome, n = 1,058 for Epiphany, n = 1,011 for C.Origami, n = 1,253 for ChromaFold, n = 1,215 for Hi-Compass). **b,** Distance-stratified Spearman correlation between predicted and experimental Hi-C contact maps as a function of genomic distance for all methods. Lines and shaded bands represent mean and standard deviation. **c,** Pairwise Spearman insulation score correlations across 14 cell types, with embedded density plots showing the distribution of per-window correlations.

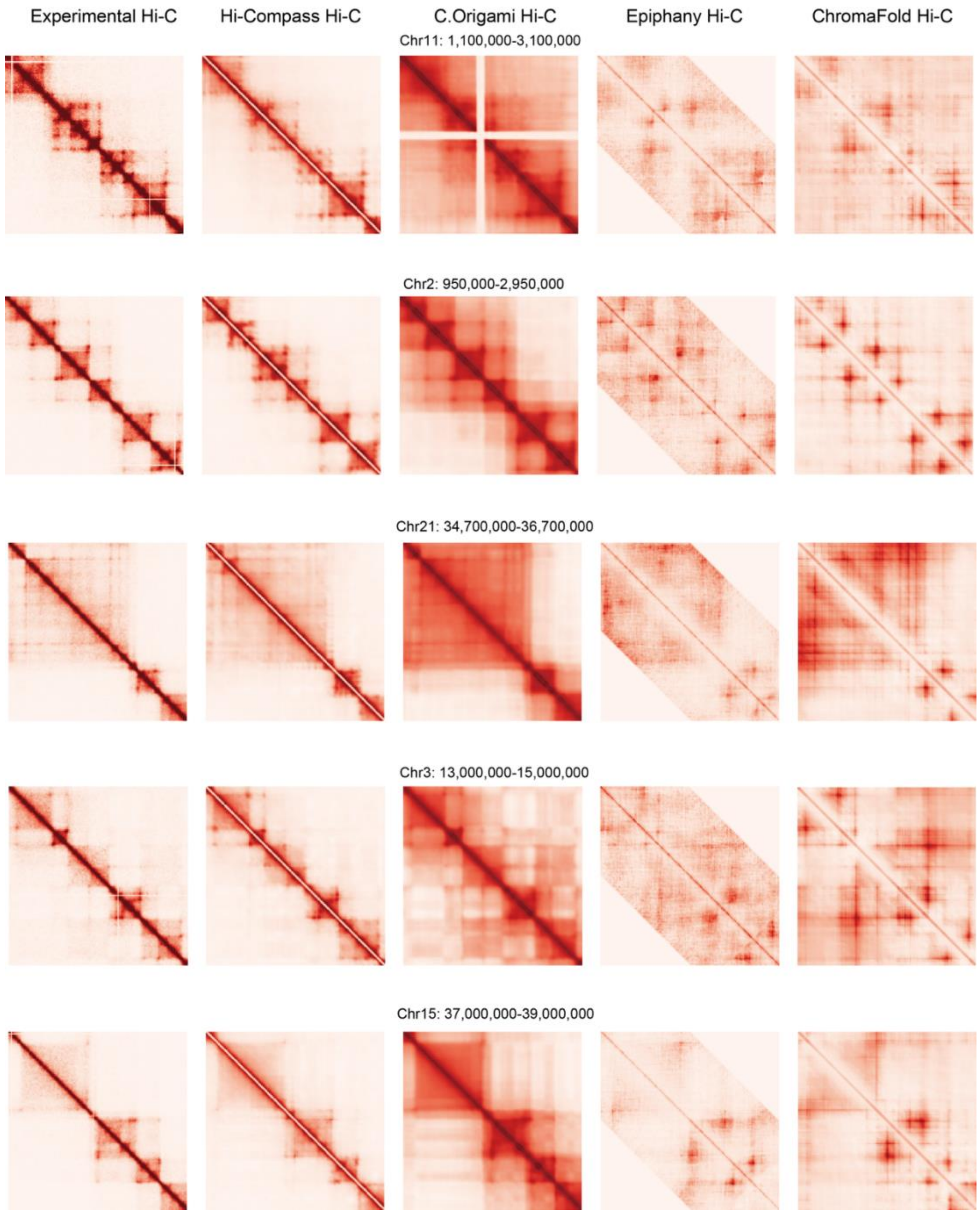

Supplementary Fig. 7 | Compared to existing methods, Hi-Compass predictions demonstrate enhanced structural resolution and prediction accuracy. Comparison of chromatin interaction predictions across representative genomic regions from Hi-Compass, C.Origami, Epiphany, and ChromaFold against experimental Hi-C data.

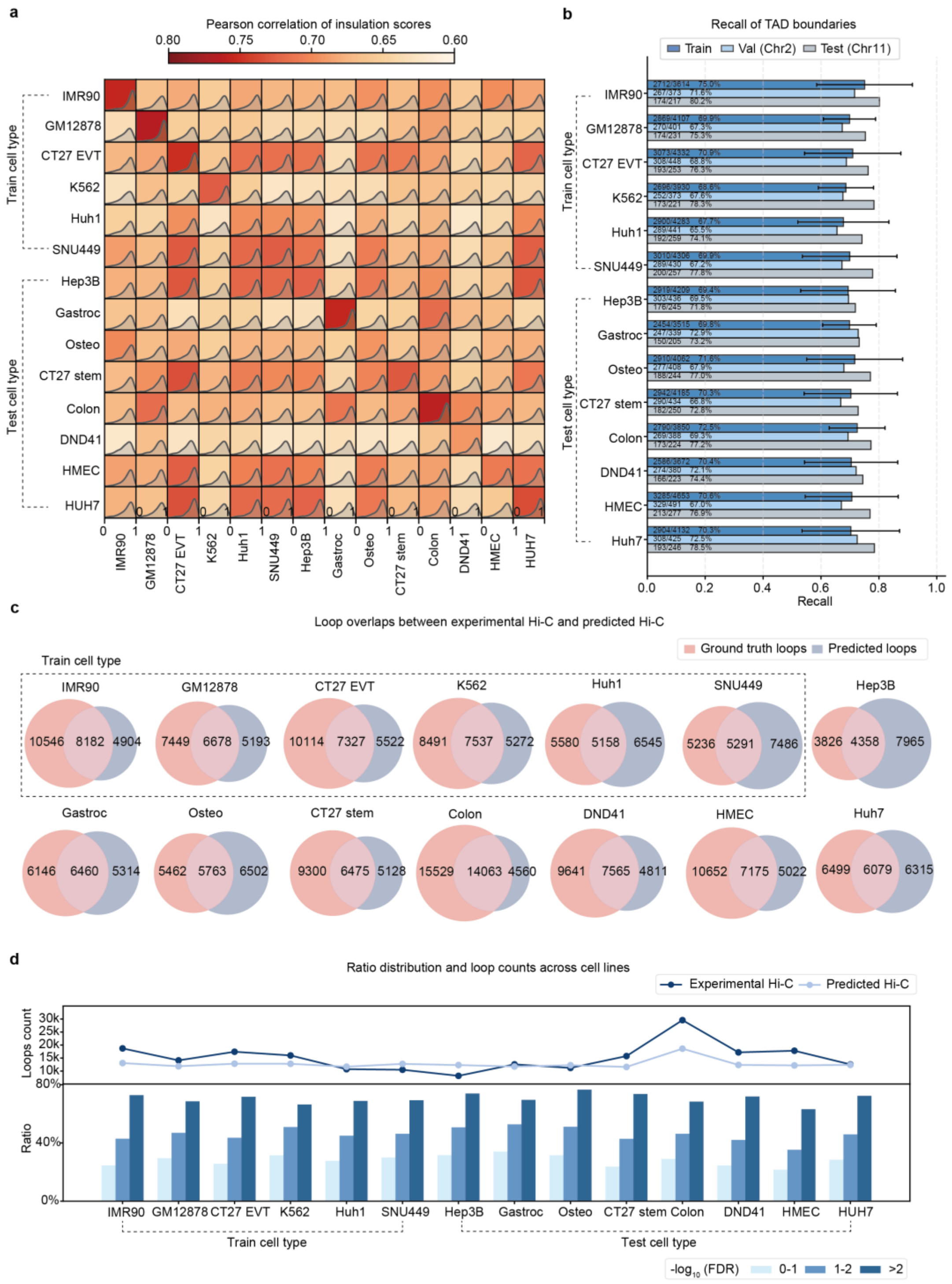

Supplementary Fig. 8 | Hi-Compass accurately recapitulates experimentally detected chromatin loops. a, Heatmap displays Pearson correlation coefficients of insulation scores between Hi-Compass predictions and experimental Hi-C data across all examined samples. **b**, Recall of TAD boundary prediction for each cell type, stratified by training chromosomes (dark blue), validation chromosome (Chr 2), and test chromosome (Chr 11). Numbers on each bar indicate the number of recalled boundaries over total boundaries and the corresponding recall percentage. Error bars represent SD across individual training chromosomes; center values represent mean recall. **c,** Venn diagrams showing the overlap between loops identified from predicted and experimental Hi-C across all samples, with loop calling performed using Mustache with identical parameters. **d,** Quantitative assessment of chromatin loop

detection performance using Hi-Compass-predicted Hi-C maps. Top: Number of loops detected by predicted and experimental Hi-C across multiple samples. Bottom: The fraction of gold-standard loops captured by predicted Hi-C maps, stratified by FDR thresholds in experimental data.

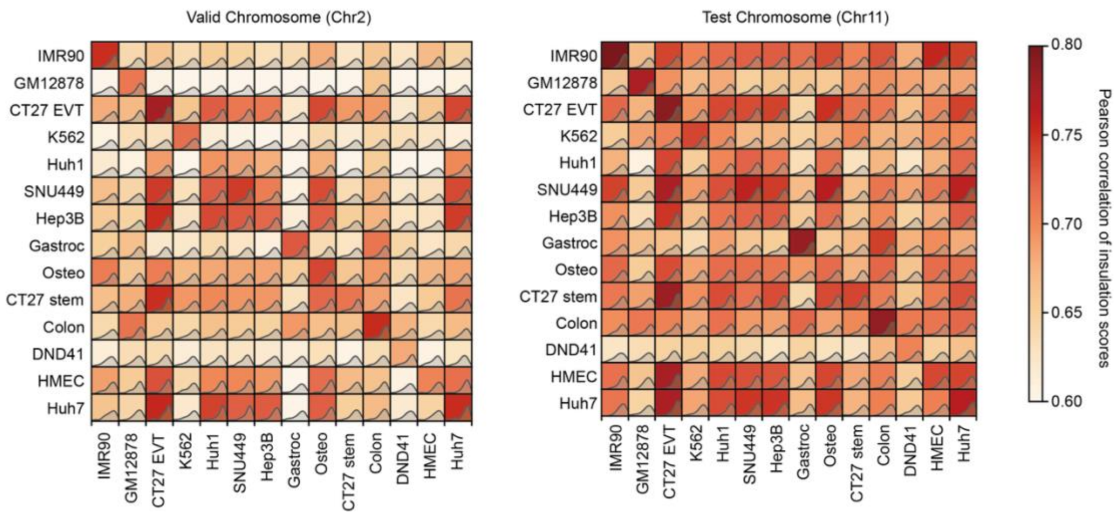

Supplementary Fig. 9 | Insulation score correlation between predicted and experimental Hi-C on held-out chromosomes. Heatmaps display Pearson correlation coefficients of insulation scores across all cell types for the validation chromosome (Chr 2) and the test chromosome (Chr 11).

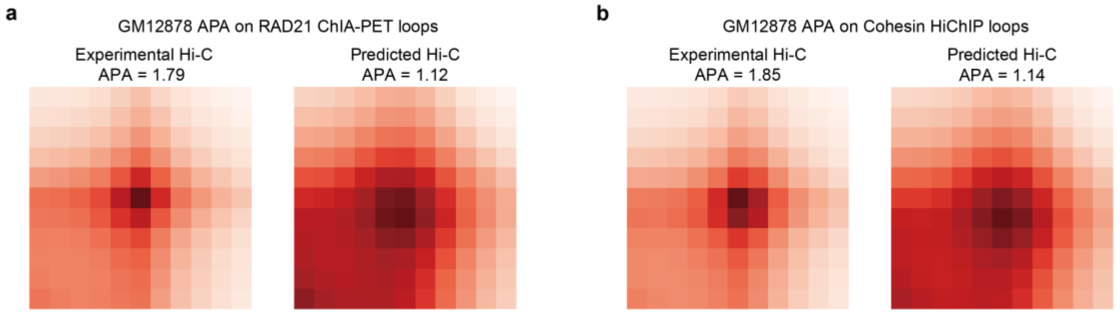

Supplementary Fig. 10 | Validation of Hi-Compass predictions against orthogonal protein-mediated chromatin interaction data. Aggregate Peak Analysis was performed by mapping RAD21 ChIA-PET loop positions (**a,** n = 5,983) and cohesin HiChIP loop positions (**b,** n = 5,337) onto experimental and Hi-Compass predicted Hi-C contact maps in GM12878. APA scores are indicated above each heatmap.

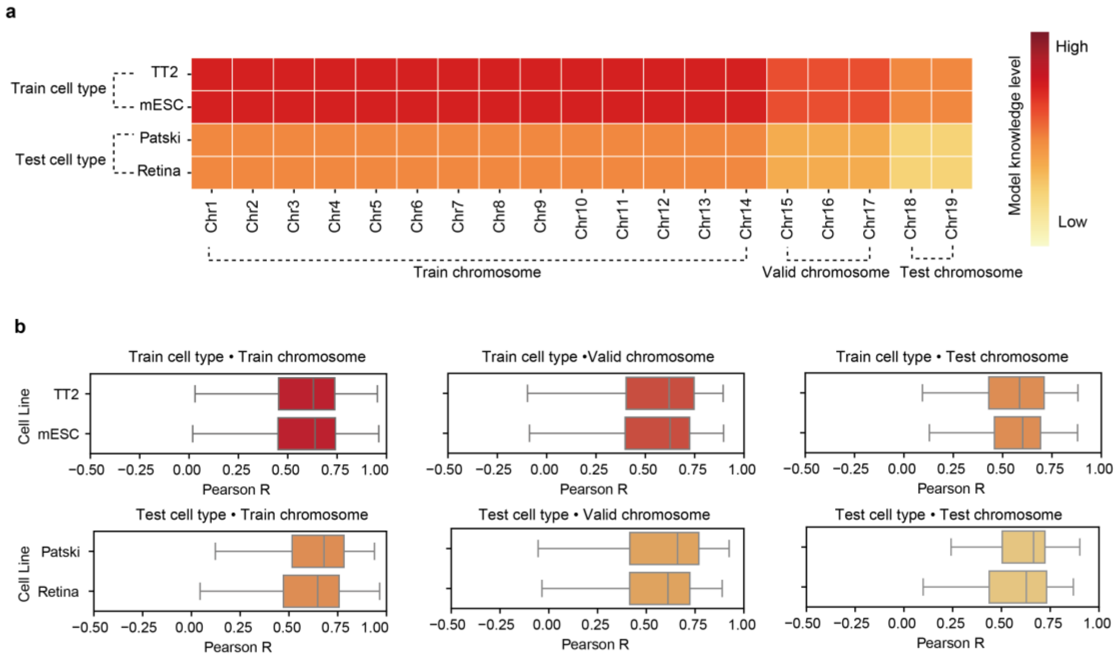

Supplementary Fig. 11 | Cross-species application of Hi-Compass to mouse genome. a, Heatmap illustrating the model knowledge level for mouse (mm10) cell types and chromosomes. The Hi-Compass model was fine-tuned on mouse Hi-C data after initial training on human data. Cell types are divided into training set and test set. Chromosomes are categorized into training set, validation set (Chr15-17), and test set (Chr18-19). **b,** Box plots showing insulation score correlation across different cell type and chromosome combinations in mouse data. Box plot colors correspond to the model knowledge levels in panel **a**. The center line represents the median, box bounds the 25th and 75th percentiles, and whiskers extend to 1.5 times the interquartile range (n = 824 for training chromosomes, 129 for validation chromosomes, 70 for test chromosomes).

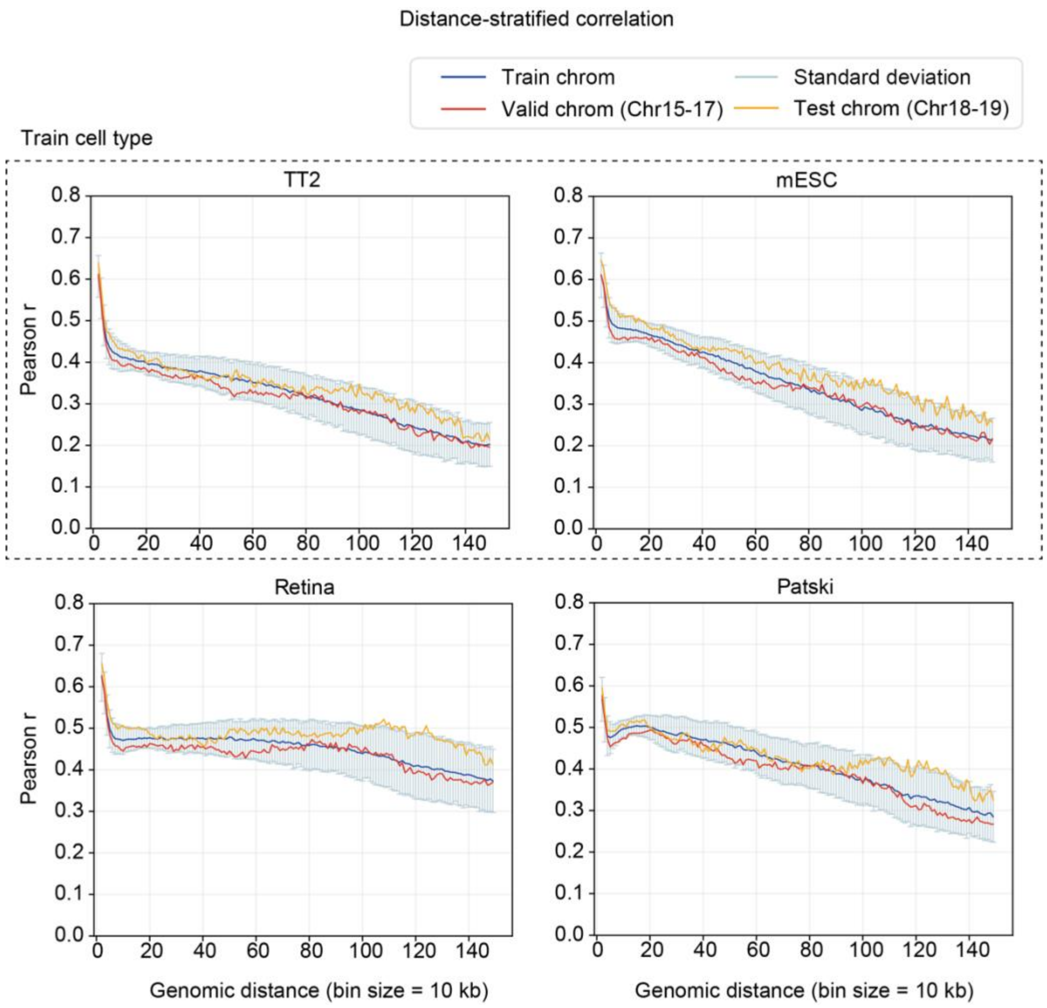

Supplementary Fig. 12 | Genome-wide distance-stratified correlation analysis of Hi-Compass predictions across mouse cell types. Line plots display Pearson correlation coefficients across genomic separation distances (matrix diagonals) for training set chromosomes (average over multiple chromosomes), validation chromosomes (Chr15-17), and test chromosomes (Chr18-19). Cell types in the dashed box are used for training.

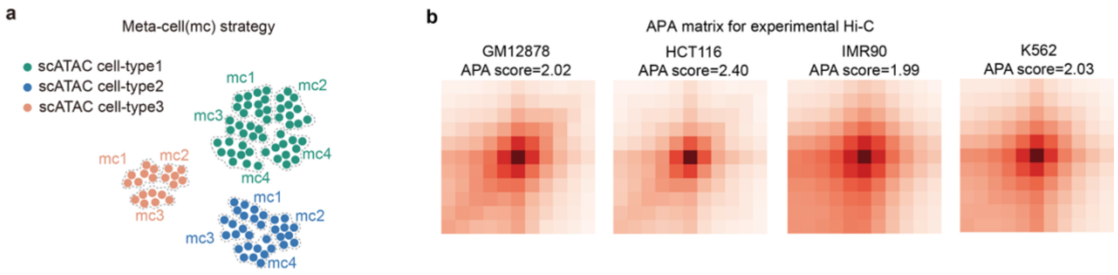

Supplementary Fig. 13 | Meta-cell strategy applied to scATAC-seq data prior to Hi-Compass prediction. a. Schematic diagram of meta-cell strategy. ATAC-seq signals from adjacent single cells are integrated, with different colors representing different cell types. **b.** APA plots show the aggregate Hi-C signals from detected loops in experimental Hi-C data of four cell lines (IMR90, HCT116, GM12878, K562).

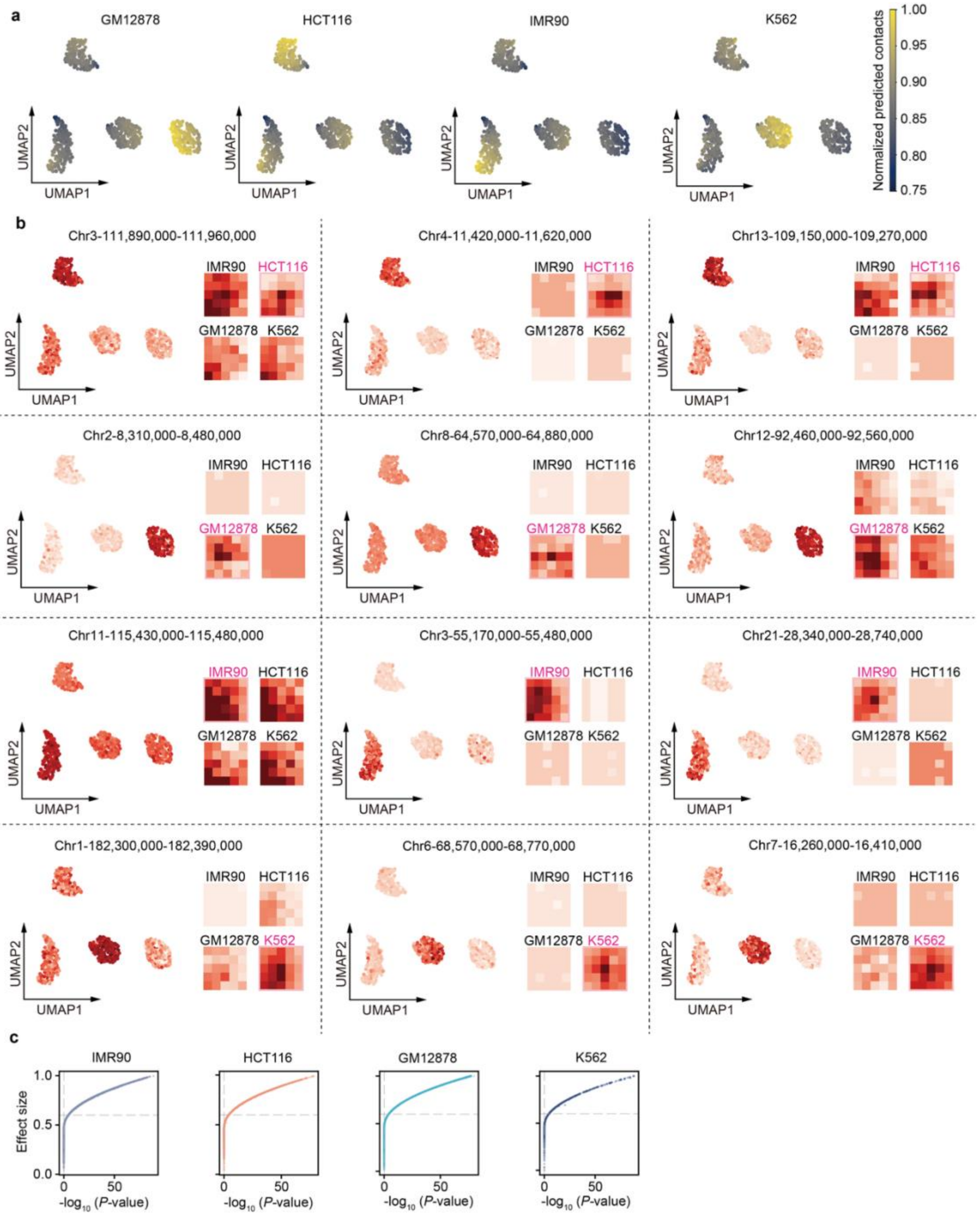

Supplementary Fig. 14 | Cell-type-specific loop analysis of Hi-Compass predictions. a. Feature plots showing the aggregated contacts at loop peaks identified from experimental Hi-C. **b.** Representative cell line-specific loops visualized by feature plots of predicted mcHi-C data and loop signal plots of experimental Hi-C data in four cell lines. Each panel shows one cell line-specific chromatin loop as example. Feature plot (left) displays the normalized predicted contacts at the loop peak in each of all meta cells. Loop signal plot (right) displays the experimental Hi-C signals at the same loop peak. **c.** Statistical analysis on loop discovery rate of predictions against experiments in four cell lines. The dashed lines indicate statistical thresholds (Mann-Whitney U test, Effect size > 0.6, $p < 0.05$).

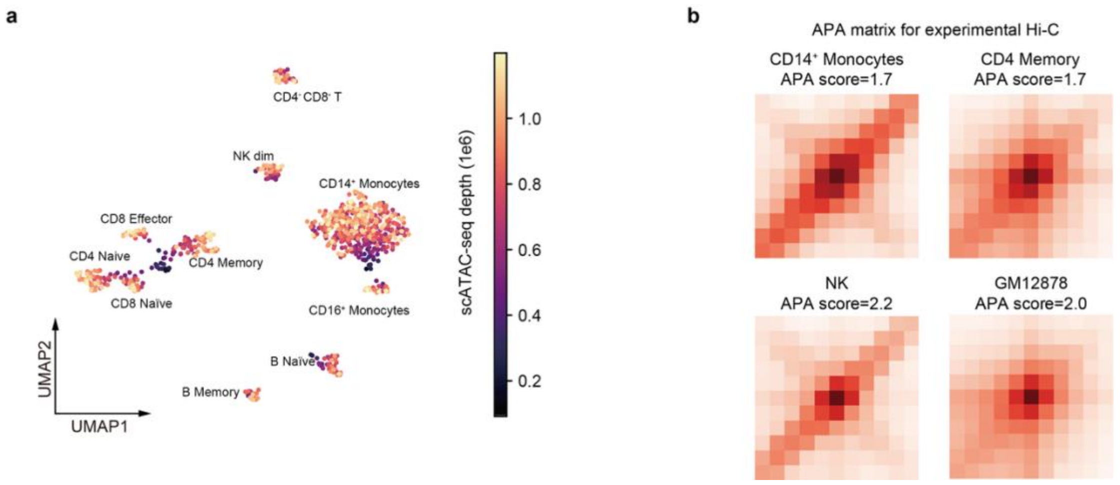

Supplementary Fig. 15 | Sequencing depth and cell type characteristic analysis of PBMC single-cell Data. a, UMAP visualization of PBMC scATAC-seq data colored by sequencing depth of input meta cell ATAC-seq signal. **b**, APA plots show the aggregate Hi-C signals from detected loops in experimental Hi-C data of four immune cell types ($CD14^+$ Monocytes, CD4 Memory, NK, and GM12878).

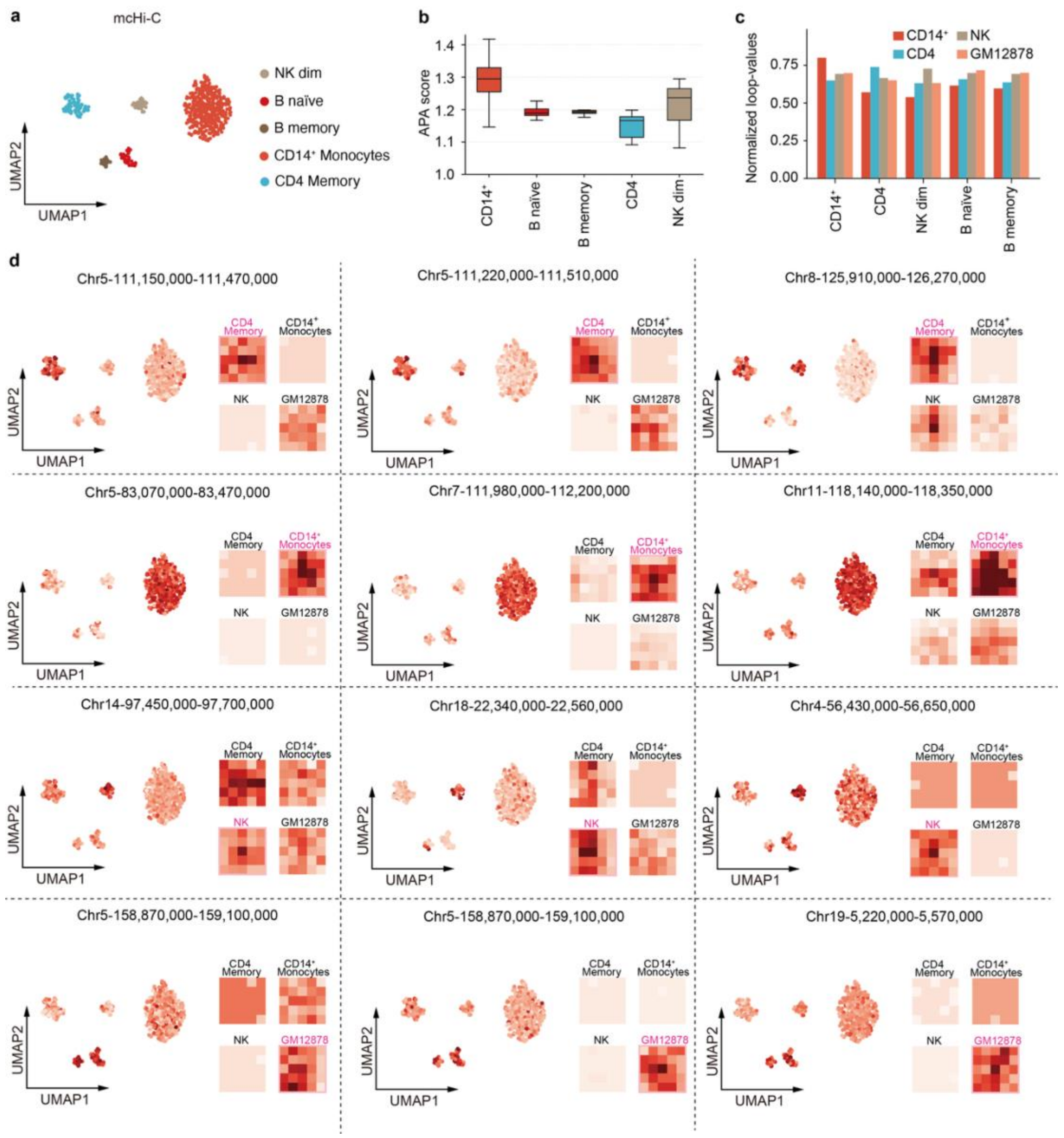

Supplementary Fig. 16 | Cell-type-specific analysis of predicted mcHi-C from PBMC scATAC-seq. **a**, UMAP visualization of predicted mcHi-C in five immune cell subtypes (NK dim, B naïve, B memory, CD14⁺ Monocytes, and CD4 Memory), with corresponding experimental Hi-C data available. This is a subset of data shown in **Fig. 4b**. **b**, Boxplot showing the distribution of APA scores per meta cell across cell types. The center line represents the median, box bounds the 25th and 75th percentiles, and whiskers extend to 1.5 times the interquartile range (n = 34 for NK dim, 34 for B naïve, 20 for B memory, 389 for CD14⁺ Monocytes, 70 for CD4 Memory). **c**, Cross-validation of cell type specificity depicted by bar plot showing normalized predicted contacts on experimentally detected loop peaks across cell types. **d**, Representative cell-type-specific loops visualized by feature plots of predicted mcHi-C data and loop signal plots of experimental Hi-C data in the five cell types. Each panel shows one cell-type-specific chromatin loop as example. Feature plot (left) displays the normalized predicted contacts at the loop peak in each of all meta cells. Loop signal plot (right) displays the experimental Hi-C signals at the same loop peak.

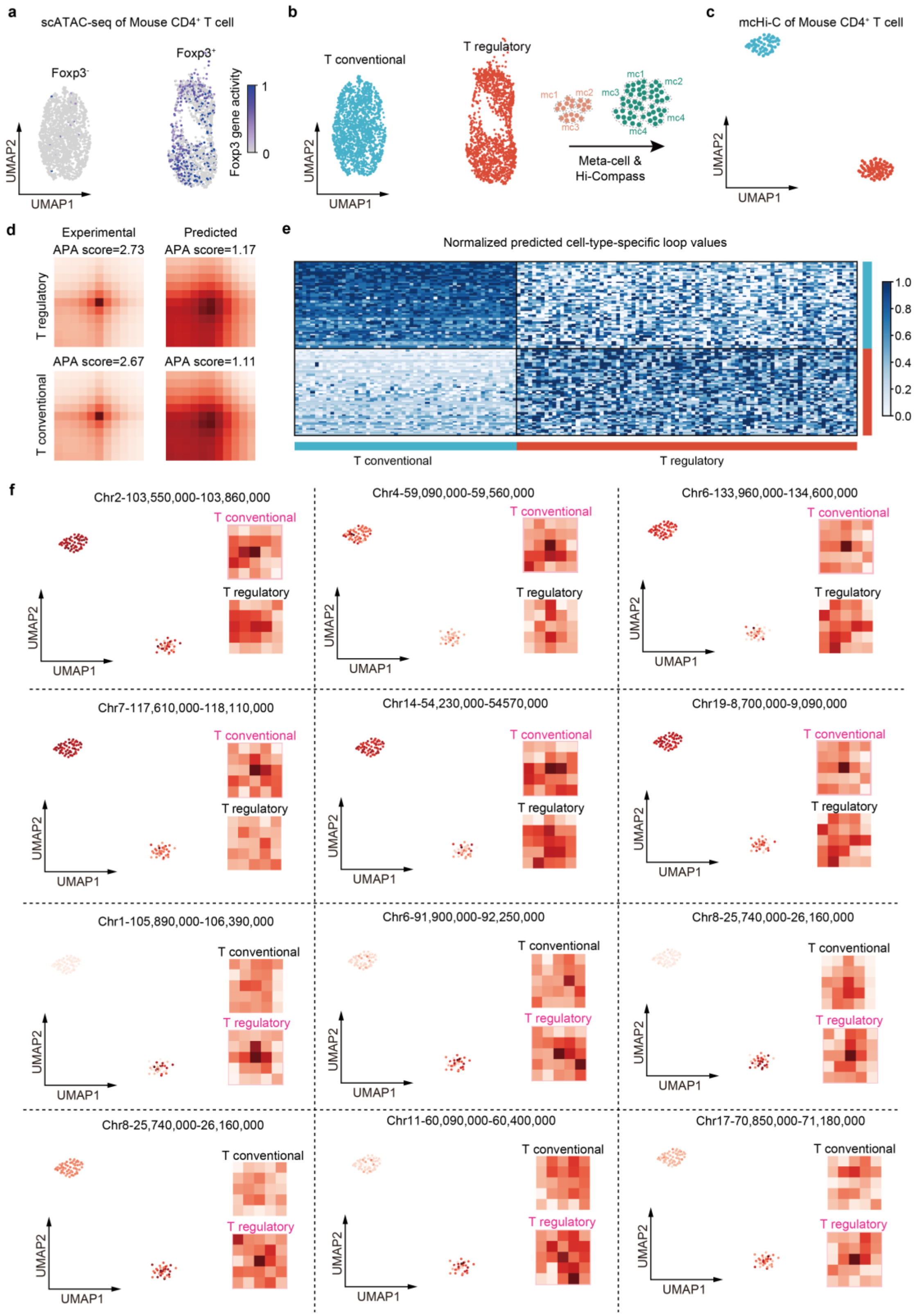

Supplementary Fig. 17 | Hi-Compass predicts cell-type-specific chromatin interactions in mouse CD4$^+$ T cells. a, UMAP projection of mouse CD4$^+$ T cell scATAC-seq data colored by Foxp3 gene activity. **b**, UMAP visualization showing regulatory T (Foxp3$^+$) and conventional T (Foxp3$^-$) cell populations. **c**, UMAP visualization of predicted mcHi-C. **d**, APA plots showing aggregated Hi-C signals from experimentally detected loops (left) and predicted mcHi-C (right) for regulatory and conventional T cells.

e, Heatmap displaying normalized predicted contacts at cell-type-specific loop peaks. Each row represents an individual loop peak, columns represent different cell types. **f**, Representative subtype-specific loops visualized by feature plots of predicted mcHi-C data. Each panel shows one subtype-specific chromatin loop. Feature plot (left) displays the normalized predicted contacts at the loop peak in each meta cell. Loop signal plot (right) displays the experimental Hi-C signals at the same loop peak.

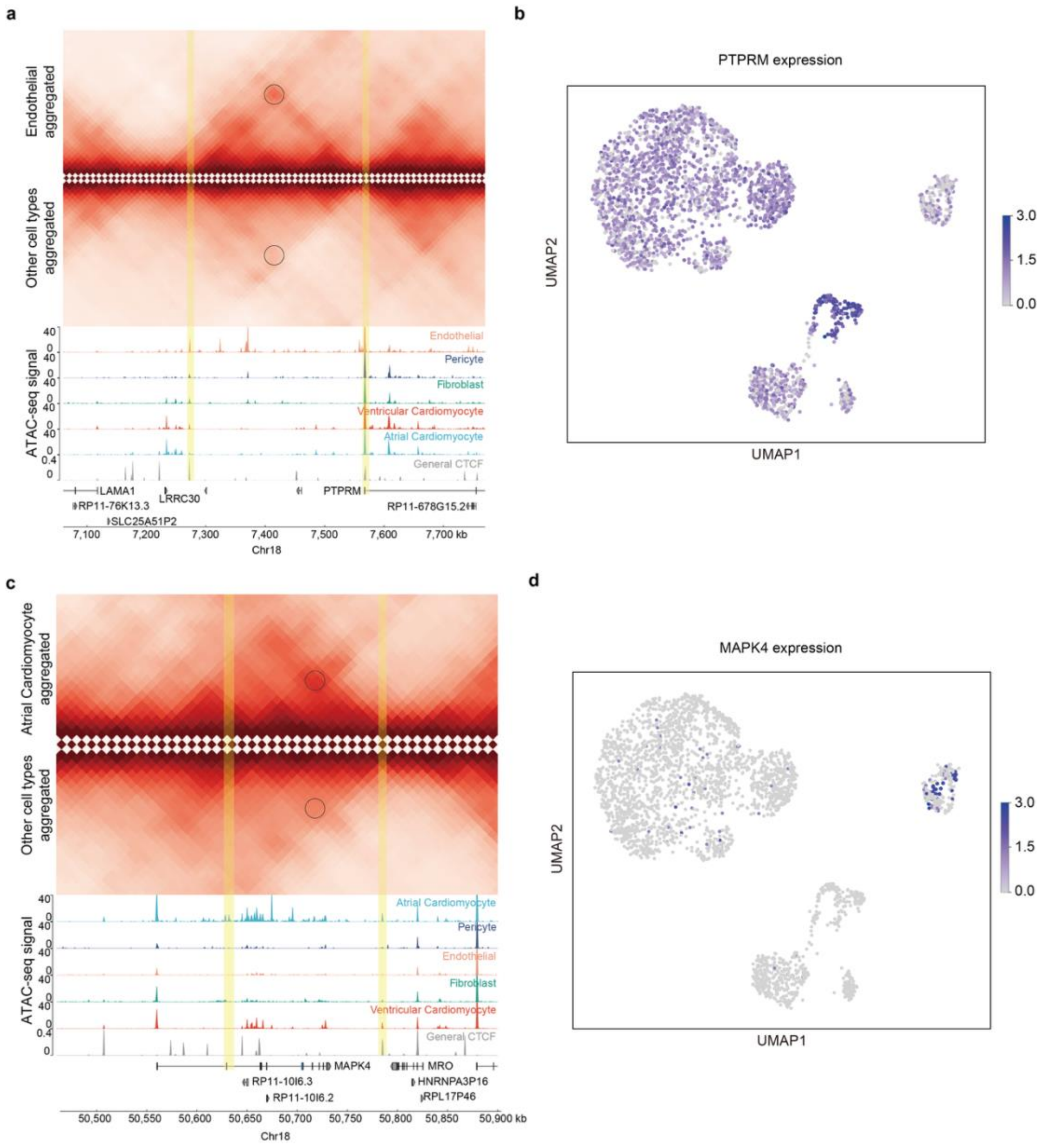

Supplementary Fig. 18 | Examples of cell-type-specific loops and associated genes. a, Predicted Hi-C profiles and corresponding ATAC-seq signal tracks for endothelial versus other cell types in the PTPRM genomic region. The loop anchored at PTPRM TSS is marked in black. Loop anchors are highlighted in yellow. **b**, Feature plot showing PTPRM is specifically expressed in endothelial cells. **c**, Predicted Hi-C profiles and corresponding ATAC-seq signal tracks for atrial cardiomyocytes versus other cell types in the MAPK4 genomic region. The loop anchored at MAPK4 TSS is marked in black. Loop anchors are highlighted in yellow. **d**, Feature plot showing MAPK4 is specifically expressed in atrial cardiomyocytes.

a

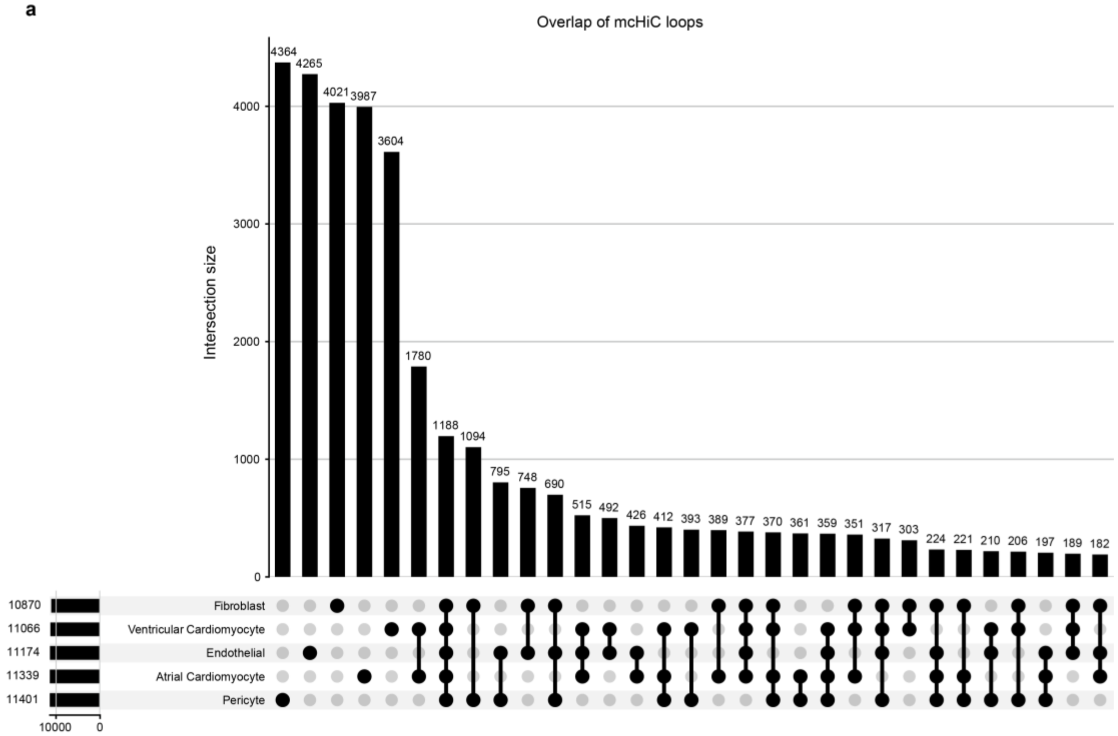

b

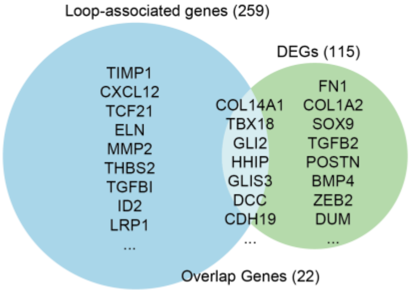

Supplementary Fig. 19 | Distribution and functional analysis of cell-type-specific loops. a, Overlap counts of mcHi-C predicted loops among cell types. The upper bar plot shows the intersection size of different combinations. The lower UpSet plot displays the distribution pattern of cell-type-specific or shared loops. **b**, Venn diagram of cell-type-specific loop-associated genes and DEGs in fibroblasts. Representative gene names are listed. Overlap contains key ECM-related genes.

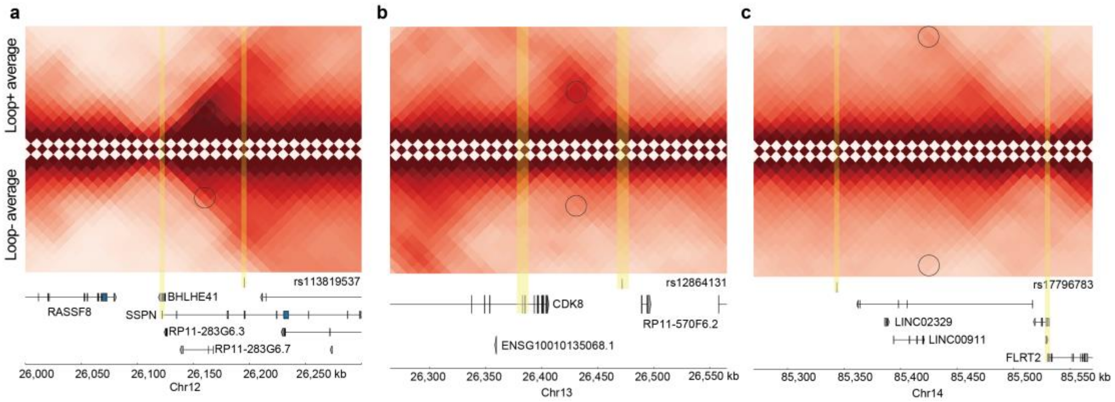

Supplementary Fig. 20 | Hi-Compass links disease variants to pathogenic genes through loops identified from predicted Hi-C. a-c, Representative examples of predicted Hi-C contact maps showing chromatin loops connecting heart disease variants to their potential target genes SSPN (**a**), CDK8 (**b**), FLRT2 (**c**). Each panel shows Hi-C interactions in embryonic heart tissue with loops (yellow highlights) connecting GWAS variants with promoters of cardiac-related genes.

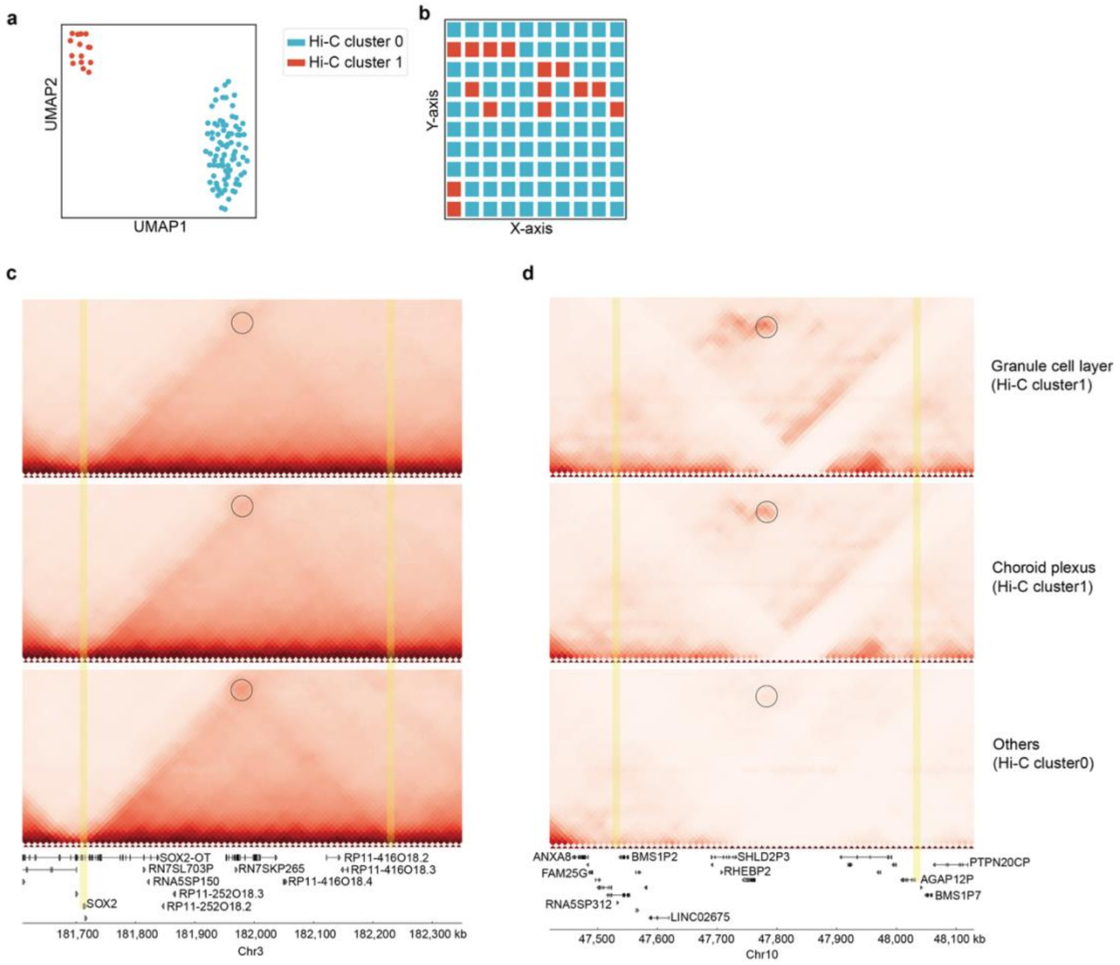

Supplementary Fig. 21 | Spatially resolved meta-spot Hi-C in different hippocampal spatial domains. a, UMAP visualization of predicted meta-spot Hi-C. **b**, Spatial distribution of the two meta-spot Hi-C clusters. **c, d**, Predicted Hi-C contact maps for SOX2 (**c**) and AGAP12P (**d**) gene regions across different hippocampal spatial domains (top: granular layer; middle: choroid plexus; bottom: others), with corresponding gene annotations displayed below the heatmaps.
