# Supplementary material for: Hi-Compass: a depth-aware deep learning framework for predicting cell-type-specific 3D genome organization from single-cell to spatial resolution
Source: Nat Commun. 2026 Apr 14;17:5172. doi: 10.1038/s41467-026-71877-z (PMC13250166; doi:10.1038/s41467-026-71877-z)
Supplement: Supplementary file 2 — Description of Additional Supplementary Files [file 41467_2026_71877_MOESM2_ESM.pdf]

Description of Additional Supplementary Files

Supplementary Data 1: CTCF ChIP-seq data used to obtain generalized CTCF profile.

Supplementary Data 2: Paired bulk ATAC-seq and Hi-C data used in this study.

Supplementary Data 3: Training and test data used for Hi-Compass.

Supplementary Data 4: Single-cell ATAC-seq, multiome and spatial ATAC-seq data used in this study.
