# Supplementary material for: Hi-Compass: a depth-aware deep learning framework for predicting cell-type-specific 3D genome organization from single-cell to spatial resolution
Source: Nat Commun. 2026 Apr 14;17:5172. doi: 10.1038/s41467-026-71877-z (PMC13250166; doi:10.1038/s41467-026-71877-z)
Supplement: Supplementary file 8 — Transparent Peer Review file [file 41467_2026_71877_MOESM8_ESM.pdf]

nature portfolio

Peer Review File

Hi-Compass: a depth-aware deep learning framework for predicting cell-type-specific 3D genome organization from single-cell to spatial resolution

Corresponding Author: Professor Hua-Jun Wu

This file contains all reviewer reports in order by version, followed by all author rebuttals in order by version.

Version 0:

Reviewer comments:

Reviewer #1

(Remarks to the Author)
Sun et al, have proposed a method for prediction of chromatin interaction using DNA sequence, open-chromatin profile and CTCF binding profile using GANs. They claim that their method can predict cell type chromatin interactions. Further for single-cell open chromatin profile and spatial scATAC-seq profile, they claim to have shown the abiliy to predict the cell-type specific interaction. Their figures look well presented and clean. Here are my comments
1.The study itself is only incremental and does not lead to conceptual or substantial advancement.
There are many existing methods doing the same thing : see ChromaFold (Gao et al., Nature Communications volume 15, 9432 (2024) , piecyk et al. (https://doi.org/10.1016/j.csbj.2022.06.047)
May be Nature communication has started publishing such incremental studies.

2. Some more serious issues are as such.
a) If you use DNA sequence to predict chromatin interaction half of the work is done as the GANs remembers the sequence form the traing-set and produces similar background interaction given some support from open-chromatin profile. It is a serious issue of information leakage in deep learning between train and test data. The training and test data share same DNA sequence and CTCF binding profile as CTCF binding is quiet conserved. The best demonstartion would be to train the GANs on few chromosomes and predict on other chromosomes. The weakness of their method is itself evident from their own statement that it does not work on mouse data, “our model was primarily trained on human cell
448 lines, and although it demonstrates good generalization to primary tissues, its performance with other
449 species or highly specialized cell types may require additional optimization. ”
Kindly prove that there is no information leakage from training to test set.

3. The visualisation based comparison is a matter brightness and histogram adustment just as I did here for their extended figure 2

Now result of C. origami looks comparable.

4. The prediction of HIC profile itself is not a good idea as it is well accepted in the field that HiC profiles are noisy and have low resolution. It is not trivial to find exact interaction between enhancers and promoters using HiC profile.
Authors should try predicting ChiA-pet or HI-ChiP, which would be more specific to cell types.

5. Authors have used classical structure of GANs, only the cost function is changed. I can not see where is the conceptual advancement on problem-novelty or out-of-box-solution .

6. while there is lack of substantial computational method advancement, there is also no fundamental finding or biological insight from trend in the predicted chromatin interaction.
The paper is suitable for journals like Briefings in bioinformatics, Bioinformatics or IEEE journals.

(Remarks on code availability)

Reviewer #2

(Remarks to the Author)
Sun, Jiang, Cai, and colleagues present Hi-Compass, a new method for predicting chromatin interactions (in the form of Hi-C maps) using DNA sequence and ATAC-seq data. Hi-Compass predictions show strong concordance with experimental Hi-C data across multiple datasets, highlighting its potential to extend chromatin studies to rare cell types where generating high-quality Hi-C maps is challenging. The manuscript is well written, logically organized, and presents an interesting approach. I have only the following suggestions that I believe could further strengthen the study:
1. In Figure 1b, it would be more informative to display the same genomic region across the three cell types. This would allow readers to assess how well Hi-Compass predicts shared versus cell-type–specific chromatin interactions, and how these compare to experimental Hi-C data. Please also clarify the resolution of Hi-C maps used in Figure 1b and elsewhere in the manuscript, as well as the resolution applied in the APA analysis. Additionally, what are the resolution limits of Hi-Compass predictions?
2. In addition to comparisons with Akita, C.Origami, and Epiphany, the authors should consider benchmarking against the latest AlphaGenome. Ideally, test sets should include Hi-C datasets published after AlphaGenome's release. It would also strengthen the study to include comparisons of prediction accuracy across multiple resolutions.
3. It is unclear whether Figures 2d and 2e represent Hi-Compass predictions or experimental Hi-C data. If they show predictions, it would be valuable to include corresponding experimental Hi-C data (especially in Fig. 2e) to demonstrate whether contrast-stretch normalization also improves visualization of real Hi-C maps.
4. The statement, "Further analysis revealed that most of the experimentally identified cell-type-specific loops were precisely captured by mcHi-C predictions with statistical significance (effect size > 0.6, $p < 0.05$)" needs clarification. Please specify the statistical test used and how effect size was defined.
5. In addition to the interesting single-cell analysis presented, I believe a broader and highly impactful application would be to predict the target genes of non-coding GWAS SNPs/mutations and identify the associated cell types. Since most GWAS signals are derived from PBMCs and may not reflect the true causal cell type, this type of analysis could greatly enhance the translational relevance of Hi-Compass. For instance, a user-friendly web application allowing researchers to upload ATAC-seq tracks and infer the most likely cell types could provide immediate, practical value to the community.
6. Currently, the package seems limited to prediction. To broaden utility, it would be helpful to provide a demo or workflow for training new models. Additionally, including WashU Browser or Juicebox session files for the predicted Hi-C maps would help readers and reviewers directly visualize and evaluate predictions.

(Remarks on code availability)
The current implementation hard-codes chromatin sizes to human, which limits applicability to widely used mouse models. In addition, only the prediction function is available, whereas the ability to train new models is equally important and should be supported. Finally, it is unclear to this reviewer why the parameter --cell_type is required—further clarification or justification would be helpful.

Reviewer #3

(Remarks to the Author)
In this paper by Sun et al, entitled "Systematic prediction of cell-type-specific chromatin interactions reveals regulatory mechanisms across biological scales", the authors present Hi-Compass, a framework for the prediction of Hi-C maps starting from cell-specific ATAC-seq data, DNA sequence and generalized CTCF binding profile. They compare the performances of the tool to other Hi-C maps predictors and demonstrate the generalization potential to other cell types. They also demonstrate the feasibility of applying the framework to sparse chromatin accessibility data such as single cell and spatial ATAC-seq. The manuscript is well written, the methods section is accurate, and the description of the analyses is in general sound and convincing. The documentation in the github page and tutorial are sufficient. In general, the manuscript can be improved with some additional details and clarifications, I provide here some suggestions to the authors.
First of all, the chose title doesn't seem appropriate and fitting to the content of the manuscript. By reading the title, one might expect a loop caller and a systematic application to identify regulatory mechanisms. However, the manuscript is more generally focused on the prediction of chromatin organization (Hi-C maps) and only provides some examples of possible chromatin loops. I suggest changing the title into one that better reflects the content.
I suggest clarifying in the main test, maybe with an additional panel in figure 1, the make up of the training/test/validation set that is, as described now, a bit confusing. Cell lines are split into training and test but also some chromosomes are split, this should be clarified. For example, in line 107 we read "To assess the generalizability of Hi-Compass, we evaluated its prediction performance on multiple unseen samples" but are these the same HiC maps generated in the previous paragraph (E.g. figure 2g with general CTCF)?
The Authors report the time and computational make up used for training, but what are computational time/requirements when applying the tool for inference?
The tutorial should contain more details on the format of the input files and on the tuning (or not) of the parameters. E.g., one of the parameters is the "depth", but how can the user define this value?
The software allows specifying the value for resolution but changing this value has not been discussed in the manuscript. Is it feasible to enhance resolution? Under which conditions? Should the user instead in some cases increase the bin size?
TAD and TAD boundaries are commented in the results and discussion sections but are never explicitly shown or calculated, as only the insulation score and its correlation are used. Authors could use the thresholding and call boundaries to verify that in fact there is a good overlap between predicted and experimental maps.

Did matrices generated with the other tools have the same resolution as Hi-Compass? E.g, in the introduction it is reported that Akita generates maps at a resolution of 2kb.
Line 173: the "image preprocessing approach" is presented as one of the key aspect of HiCompass but it has not been previously discussed in the test. It should be mentioned when the pipeline is presented.
Figure 2g: I suggest highlighting in figure that the last three cell lines are not used during training.
Figure 2i: What is the continuous signal shown at the top and bottom of this figure? Also the figure legend contains a text fragment that does not belong to this figure (line 787-790).
Line 225: the reference to figure 2c here is wrong. Other than Ext fig 3b there does not seem to be a panel related to this result.
Line 226: I find the expressions "experimentally identified loops" and "Hi-Compass-predicted loops" a bit misleading because what is meant here is "loops identified from experimental Hi-C and loops identified from predicted Hi-C". Hi-Compass does not directly predict loops.
Fig 5f: I would use a different combination of colors as red/blue is normally used for enrichment in up-and down-regulated genes.
Line 335: The reference to "developmental stages" is not clear. What is this referring to?
Line 337: This phrase is missing a verb, please review.
Line 345: this enrichment analysis is not clearly discussed, what is the ratio depicted in figure 5g?
Line 380: why were these 2 genes chosen? Are these known specific markers? Moreover, they have opposite behavior in gene expression, this should be better explained in the text.
Line 386: it is not clear how the super resolution is obtained and how it is different from the meta-spot approach described previously.
Line 422: the discussion mentions "multi-omics integration capability of Hi-Compass" but it appears that Hi-Compass doesn't perform direct integration of different modalities as the RNA was used in downstream analyses to validate the quality of the differential loops.

(Remarks on code availability)
I did not try to install the code but the documentation should be improved, as reported in my comments for the authors.

Version 1:

Reviewer comments:

Reviewer #1

(Remarks to the Author)
Even though Sun et al. have tried their best to response to the comments, there are a few serious concerns

1. They are able to recall only 65% of TAD boundaries by prediction. Given the fact that TAD boundaries are highly preserved even across cell-type and there is information leakage of genomic sequence (or loci), it is not a great result. As even if you do not run any prediction model and overlap boundary from one cell-type to other you can get 65% or more overlap. (see McArthur et al. , AJHG, https://doi.org/10.1016/j.ajhg.2021.01.001)
Moreover authors have not shown results about TAD boundary preservation on test chromosomes for which there was no training.

2. Kindly try to show spearman correlation, not pearson correlation.

3. In supplementary figure-4, author have shown that on test data, (chromosomes 11) the prediction is better than even training data (other chromosomes), how can it be theoretically possible. May be it is at level of insulation score. However in mouse genome based prediction for test chromosome how can distance-stratified correlation be better than training set chromosomes. Can author provides some insight about it or is it just a mistake.

4. One comment completely ignored : Authors are using high-resolution ATAC-seq profile and they claim high resolution interaction prediction, then, why they could not produce result to my comment on overlapping with ChiA-pet and Hi-ChIP interaction. Authors should also benchmark on interaction from ChiA-pet or Hi-ChIP.

(Remarks on code availability)

Reviewer #2

(Remarks to the Author)
The authors have addressed all my suggestions.

(Remarks on code availability)
The authors have addressed all my suggestions. The code was also revised according to reviewers' suggestions.

Reviewer #3

(Remarks to the Author)
I would like to thank the authors for their thorough responses to my previous comments. I am satisfied with the majority of the revisions made; however, I have one remaining concern and a couple minor observations.
In the application of Hi-Compass on spatial data, the text reports “AGAP12P, which showed elevated expression in the granular layer (Fig. 6e), and SOX2, a well-characterized transcription factor essential for neural stem cell maintenance and neurogenesis that was enriched in the choroid plexus” but all figures seem to point out that AGAP12P is expressed both in granular layer and choroid plexus (Fig 6g and clusters R4 and R6 in the UMAP of figure 6i) whereas SOX2 is not expressed in such tissues and expressed in all other areas (Figure 6h and 6j). Also interactions in Figure 6k and 6l and supplementary figure 18c 18d support this.
Moreover, the panels of figure 6 are mis-aligned with the description in the figure legend and are also not correctly referenced with the text in the results section.
Finally, I appreciate that the authors included a scheme about the training and test. When not explicitly stated, should it be assumed that the shown results refer to the test chromosomes? E.g. in supplementary figure 7a are correlation scores relative to only the test chromosome?

(Remarks on code availability)

Version 2:

Reviewer comments:

Reviewer #1

(Remarks to the Author)
No comment from me

(Remarks on code availability)
the manuscript has improved now

but I could not install the tool according to the instruction on github page

Reviewer #3

(Remarks to the Author)
The authors have addressed all my previous points. I am satisfied with the current version of the paper and have no further comments to add.

(Remarks on code availability)

Open Access This Peer Review File is licensed under a Creative Commons Attribution 4.0 International License, which permits use, sharing, adaptation, distribution and reproduction in any medium or format, as long as you give appropriate credit to the original author(s) and the source, provide a link to the Creative Commons license, and indicate if changes were made.
In cases where reviewers are anonymous, credit should be given to 'Anonymous Referee' and the source.
The images or other third party material in this Peer Review File are included in the article's Creative Commons license, unless indicated otherwise in a credit line to the material. If material is not included in the article's Creative Commons license and your intended use is not permitted by statutory regulation or exceeds the permitted use, you will need to obtain permission directly from the copyright holder.
To view a copy of this license, visit https://creativecommons.org/licenses/by/4.0/

Dear Reviewers,

We would like to express our sincere gratitude to the three reviewers for providing constructive and insightful comments. We have conducted a major revision following these suggestions. Specifically, we have rewritten the related text, added new results, updated figures and supplementary materials, and substantially expanded the software functionality (newly added: **Supplementary Figs. 2, 4, 5, 8, 9, 14;** revised: **Figs. 2d-j, 5f, Supplementary Figs. 6, 7;**). To accommodate the reviewers' comments, we made the following major changes to the paper:

1. **Enhanced evaluation of generalization:** We added systematic evaluation of model performance stratified by both cell type and chromosome categories, demonstrating that Hi-Compass maintains robust performance even on completely unseen combinations of genomic regions and cellular contexts (new: **Supplementary Fig. 6**).

2. **Cross-species generalization**: We collected four matched ATAC-seq and Hi-C datasets from mouse cell lines and demonstrated that Hi-Compass can be efficiently adapted to the mouse genome through fine-tuning, achieving robust performance on unseen mouse cell types (new: **Supplementary Figs. 7-8;** revised: **Supplementary Table 2**).

3. **Single-cell application in mouse**: We applied the fine-tuned mouse model to scATAC-seq data of mouse splenic $CD4^+$ T cells (Treg and Tcon subtypes), demonstrating its cross-species generalization capacity at the single-cell level (new: **Supplementary Fig. 14**).

4. **Software expansion**: We have substantially expanded the Hi-Compass software package to include: (1) data preprocessing pipelines for ATAC-seq downsampling and Hi-C matrix normalization; (2) model training functionality supporting human, mouse, and custom species; and (3) prediction capabilities for any user-defined genome. All available at https://github.com/EndeavourSyc/Hi-Compass. A revised advanced tutorial demonstrating the complete workflow, from meta-cell Hi-C prediction to loop calling, loop annotation, and GWAS SNP-to-gene annotation, is now updated and available at https://github.com/EndeavourSyc/Hi-Compass/blob/main/hicompass/doc/Advanced_Tutorial.md.

5. **Methodological clarifications**: We clarified the training/validation/test set design, resolution settings, TAD boundary calling methods, statistical tests, and computational time requirements throughout the manuscript and Methods section.

6. **Improved descriptions**: We revised multiple descriptions for clarity, including the distinction between "loops identified from experimental Hi-C" versus "loops identified from predicted Hi-C", the GWAS enrichment analysis methodology, and the super-resolution approach for spatial data.

Please find below a point-by-point response to each of the comments raised by referees (in blue) and our response (in black). The corresponding edits in the revised manuscript are highlighted in red.

Reviewer #1 (Remarks to the Author):

Sun et al, have proposed a method for prediction of chromatin interaction using DNA sequence, open-chromatin profile and CTCF binding profile using GANs. They claim that their method can predict cell type chromatin interactions. Further for single-cell open chromatin profile and spatial scATAC-seq profile, they claim to have shown the abiliy to predict the cell-type specific interaction. Their figures look well presented and clean.

Response: We thank the reviewer for the careful evaluation of our manuscript. We have thoroughly addressed all concerns raised by the reviewer, particularly regarding the novelty of our approach, potential information leakage, and cross-species generalization. Below, we provide detailed point-by-point responses to each specific comment.

Comment 1: The study itself is only incremental and does not lead to conceptual or substantial advancement. There are many existing methods doing the same thing: see ChromaFold (Gao et al., Nature Communications volume 15, 9432 (2024)), piecyk et al. (https://doi.org/10.1016/j.csbj.2022.06.047). May be Nature communication has started publishing such incremental studies.

Response: We thank the reviewer for this comment and the opportunity to clarify the contributions of Hi-Compass. While several methods exist for Hi-C prediction, Hi-Compass addresses critical limitations that have not been solved by previous approaches.

First, Hi-Compass requires only ATAC-seq as cell-type-specific input, substantially expanding its applicability to cell types lacking matched CTCF ChIP-seq data. This is in contrast to C.Origami which requires cell-type-specific CTCF data that is often unavailable. Second, unlike existing methods such as ChromaFold which require joint profiling of bulk Hi-C and scATAC-seq for each sample of interest and necessitate model retraining for new applications, Hi-Compass is designed with multi-cell-type training and operates in a true zero-shot manner, enabling direct prediction on unseen cell types without requiring additional training. Third, Hi-Compass incorporates a depth-aware training framework that dynamically adapts to varying sequencing depths, enabling systematic prediction of cell-type-specific Hi-C maps from single-cell and spatial ATAC-seq data, which is an application that can't be achieved by any existing method. Fourth, in this revision, we demonstrate that Hi-Compass can be efficiently adapted across species through lightweight fine-tuning, achieving robust performance on unseen cell types in mouse (new **Supplementary Figs. 7-8, 13** below). Lastly, Hi-Compass significantly outperforms previous methods in terms of insulation score correlation and structural similarity (19% and 31% improvement over the second-best performing method) (revised **Fig. 2f-h** below). We think such improvement is critical for Hi-C prediction field.

As a computational method, Hi-Compass is designed to serve as a tool that enables researchers to explore 3D genome biology in previously inaccessible contexts. By enabling Hi-C prediction from single-cell and spatial ATAC-seq data, researchers can now investigate cell-type-specific and spatially resolved

chromatin interactions with low cost. The applications demonstrated in this work illustrate how this approach can support new biological discovery across diverse research domains.

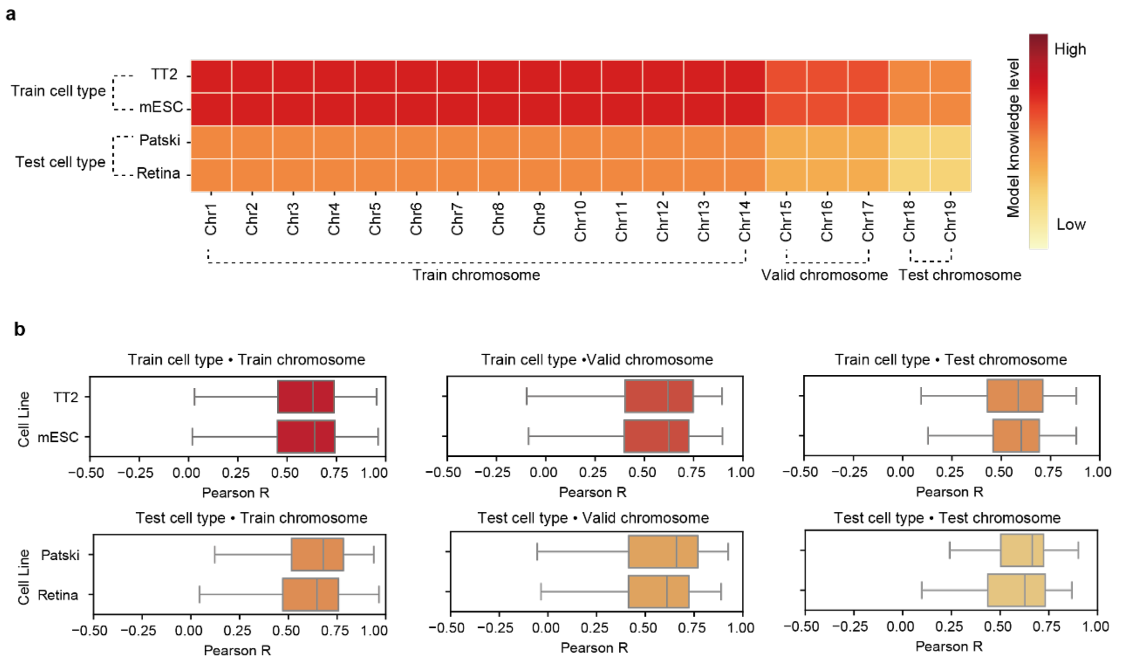

(Legends: ***Supplementary Fig. 8 | Cross-species application of Hi-Compass to mouse genome. a,*** *Heatmap illustrating the model knowledge level for mouse (mm10) cell types and chromosomes. The Hi-Compass model was fine-tuned on mouse Hi-C data after initial training on human data. Cell types are divided into training set and test set. Chromosomes are categorized into training set, validation set (Chr15-17), and test set (Chr18-19).* ***b,*** *Box plots showing insulation score correlation across different cell type and chromosome combinations in mouse data. Box plot colors correspond to the model knowledge levels in panel* ***a****.*)

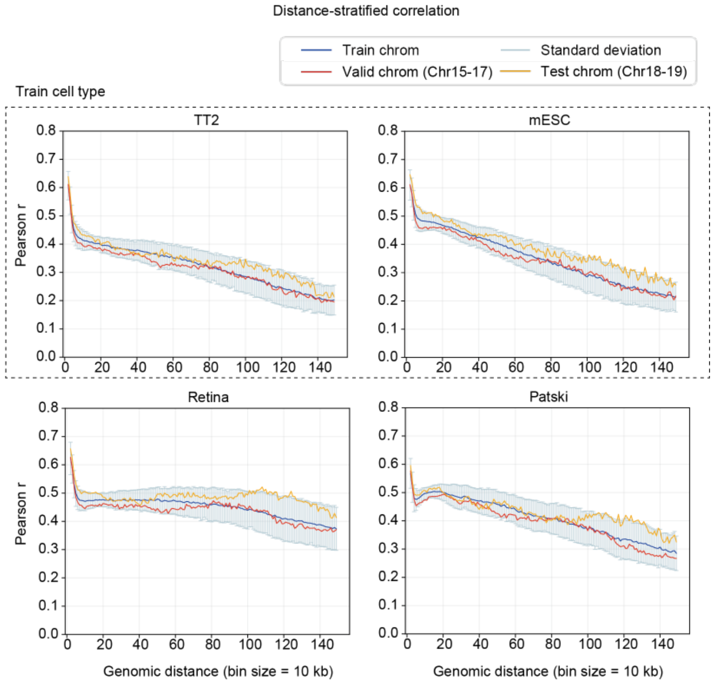

(Legends: ***Supplementary Fig. 9 | Genome-wide distance-stratified correlation analysis of Hi-Compass predictions across mouse cell types.*** *Line plots display Pearson correlation coefficients across genomic separation distances (matrix diagonals) for training set chromosomes (average over multiple chromosomes), validation chromosomes (Chr15-17), and test chromosomes (Chr18-19). Cell types in the dashed box are used for training.)*

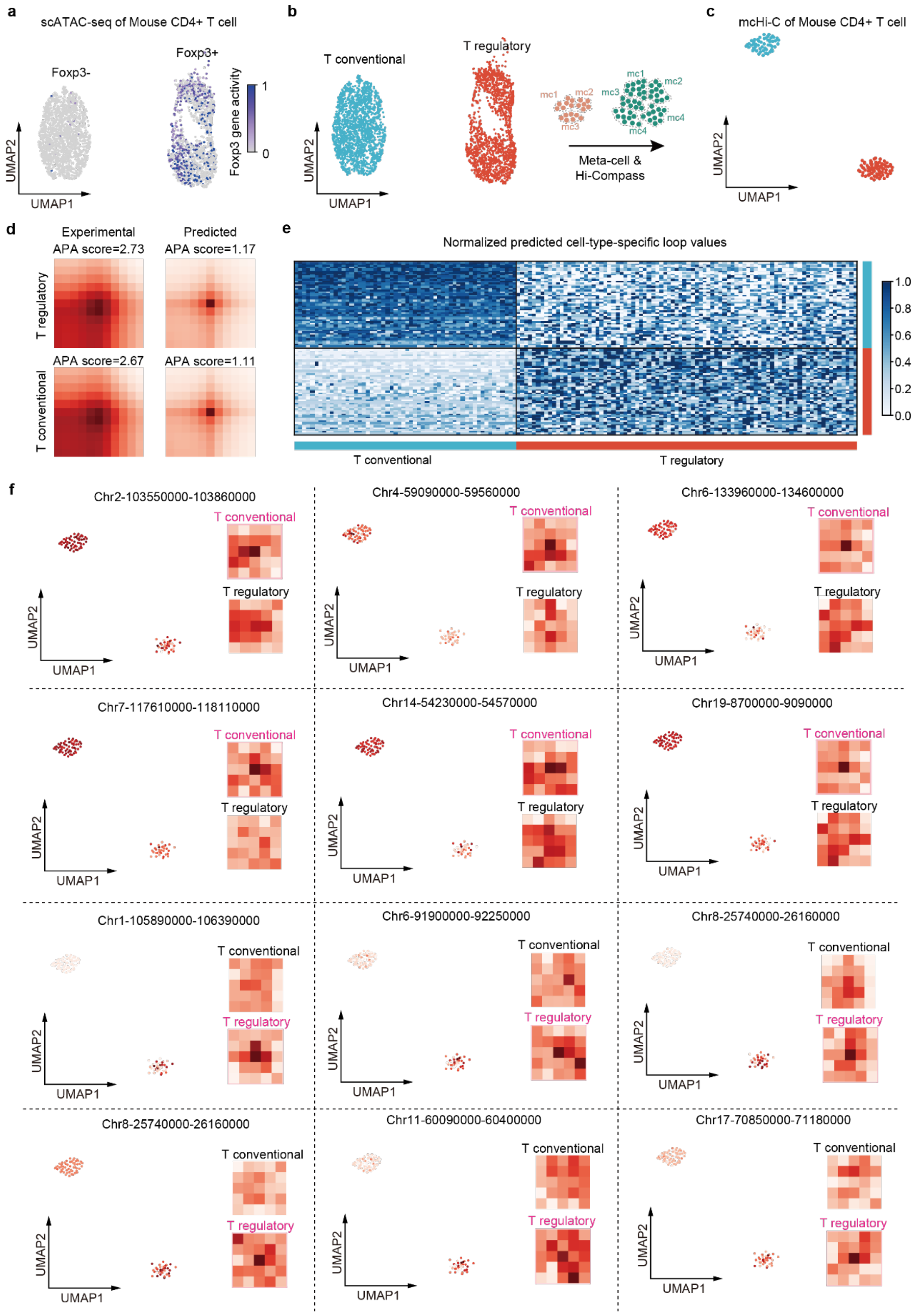

*(Legends: **Supplementary Fig. 14 | Hi-Compass predicts cell-type-specific chromatin interactions in mouse CD4⁺ T cells. a**, UMAP projection of mouse CD4⁺ T cell scATAC-seq data colored by Foxp3 gene activity. **b**, UMAP visualization showing regulatory T (Foxp3⁺) and conventional T (Foxp3⁻) cell populations. **c**, UMAP visualization of predicted mcHi-C. **d**, APA plots showing aggregated Hi-C signals from experimentally detected loops (left) and predicted mcHi-C (right) for regulatory and conventional T cells. **e**, Heatmap displaying normalized predicted contacts at cell-type-specific loop peaks. Each row represents an individual loop peak, column represents different cell types. **f**, Representative subtype-specific loops visualized by feature plots of predicted mcHi-C data. Each panel shows one subtype-specific chromatin loop. Feature plot (left) displays the normalized predicted contacts at the loop peak in each meta cell. Loop signal plot (right) displays the experimental Hi-C signals at the same loop peak.)*

Comment 2: Some more serious issues are as such. a) If you use DNA sequence to predict chromatin interaction half of the work is done as the GANs remembers the sequence from the training-set and produces similar background interaction given some support from open-chromatin profile. It is a serious issue of information leakage in deep learning between train and test data. The training and test data share same DNA sequence and CTCF binding profile as CTCF binding is quiet conserved. The best demonstration would be to train the GANs on few chromosomes and predict on other chromosomes. The weakness of their method is itself evident from their own statement that it does not work on mouse data, "our model was primarily trained on human cell lines, and although it demonstrates good generalization to primary tissues, its performance with other species or highly specialized cell types may require additional optimization." Kindly prove that there is no information leakage from training to test set.

Response: We thank the reviewer for raising this concern. We would like to address several points.

First, we would like to clarify that Hi-Compass does not employ a GAN (Generative Adversarial Network) architecture. The defining feature of GANs is adversarial training, where a generator produces synthetic samples to deceive a discriminator that distinguishes real from fake data. In Hi-Compass, the discriminator is simply an auxiliary classifier that predicts cell type identity from the output Hi-C matrix, providing additional supervision through cross-entropy loss. This is a standard multi-task learning design rather than adversarial training. The model architecture is detailed in **Supplementary Fig. 1**.

Regarding the concern about information leakage between training and test data: our training/test split is designed precisely to address this point. Chromosomes 2 and 11 are completely held out from training (as validation and test sets, respectively), and 8 cell types are reserved exclusively for testing. This means our most stringent evaluation is performed on genomic regions the model has never seen during training. We have clarified this design in the revised manuscript with a new figure (new **Supplementary Fig. 4a**) and added systematic evaluation showing that Hi-Compass maintains robust performance even on held-out chromosomes in held-out cell types (new **Supplementary Fig. 4b**). If the model were simply memorizing DNA sequences, we would expect a dramatic performance drop on unseen chromosomes, which is not observed.

As for CTCF, the generalized profile is an intentional design choice, not a limitation. By using a pan-tissue CTCF profile rather than cell-type-specific data, Hi-Compass can be applied to cell types lacking CTCF

ChIP-seq data. The cell-type specificity of our predictions comes primarily from ATAC-seq input, as evidenced by the strong diagonal pattern in our cross-cell-type correlation analysis in **Fig. 2i**.

Regarding cross-species generalization, we have now collected matched ATAC-seq and Hi-C data from mouse cell lines and systematically evaluated this point. We fine-tuned the pre-trained human model on two mouse cell types (TT2, mESC) and tested on another two completely unseen mouse cell types (Patski, Retina), with chromosomes 15-17 as validation and chromosomes 18-19 as test set (new **Supplementary Fig. 8a**). After fine-tuning, Hi-Compass achieved robust performance on mouse data, with insulation score correlations comparable to human samples (new **Supplementary Fig. 8b**). Distance-stratified correlation analysis also confirmed consistent prediction accuracy across genomic distances (new **Supplementary Fig. 9**). We further applied the fine-tuned model to mouse scATAC-seq data of splenic $CD4^+$ T cells and successfully distinguished regulatory T cells from conventional T cells based on predicted chromatin interactions (new **Supplementary Fig. 14**).

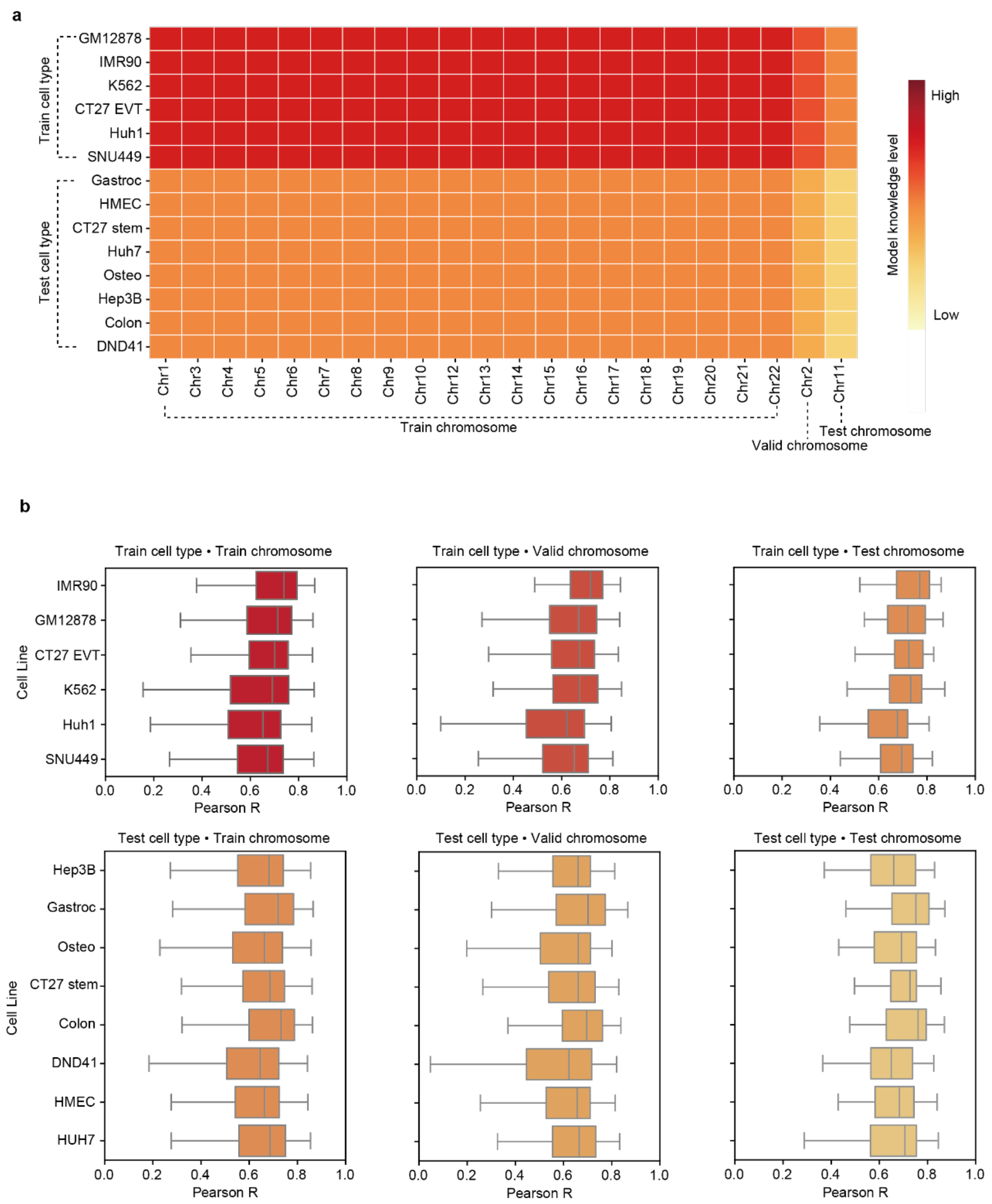

(Legends: ***Supplementary Fig. 4 | Evaluation of Hi-Compass generalization across cell types and chromosomes in human. a****, Heatmap showing model knowledge level across cell types (rows) and chromosomes (columns). Cell types are divided into training set and test set. Chromosomes are categorized into training, validation (Chr2), and test (Chr11) sets.* ***b****, Box plots showing insulation score correlation across different cell type and chromosome combinations. Box plot colors correspond to the model knowledge levels in panel* ***a****.)*

Comment 3: The visualization based comparison is a matter brightness and histogram adjustment just as I did here for their extended figure 2. Now result of C. origami looks comparable.

Response: We thank the reviewer for this observation. Our contrast stretching normalization is indeed designed to enhance the visibility of Hi-C structures. We would like to note that this preprocessing is a standardized transformation applied uniformly to all Hi-C data before model training, ensuring consistency across all samples and methods in our comparisons.

More importantly, the clearer visualization in our results reflects more accurate reconstruction of fine-scale chromatin structures. While one may view **Supplementary Fig. 6** as a visual comparison, the underlying difference is that Hi-Compass better captures structural details such as TAD boundaries and local interaction patterns. This improved structural fidelity is supported by our quantitative evaluations, in terms of insulation score correlation and structural similarity (19% and 31% improvement over the second-best performing method) (revised **Fig. 2f-h**), demonstrating that the visual clarity corresponds to genuine improvements in prediction quality.

Comment 4: The prediction of HIC profile itself is not a good idea as it is well accepted in the field that HiC profiles are noisy and have low resolution. It is not trivial to find exact interaction between enhancers and promoters using HiC profile. Authors should try predicting ChiA-pet or HI-ChiP, which would be more specific to cell types.

Response: We thank the reviewer for this suggestion. We agree that ChIA-PET and HiChIP are valuable techniques that provide specific views of protein-mediated interactions. Our focus on Hi-C prediction is motivated by several considerations. Hi-C captures genome-wide chromatin interactions without targeting specific proteins, providing a comprehensive view of chromatin organization that includes both TAD structures and chromatin loops within a single assay. This comprehensive nature is beneficial not only for downstream biological applications, but also for model training, enabling the model to learn generalizable principles of chromatin folding rather than protein-specific interaction patterns. Regarding enhancer-promoter interactions, Hi-C has been extensively used to identify chromatin loops, including enhancer-promoter and SNP-promoter interactions (Applied to cell line – Jin et, al. 2013 Nature [PMID: 24141950] and Rao et, al. 2014 Cell [PMID: 25497547]; Applied to human brain - Won et, al. 2016 Nature [PMID: 27760116]; Applied to cancer - Flavahan et, al 2016 Nature [PMID: 26700815] and Rahme et, al. 2023 Cell [PMID: 37494934]; Applied to neural development and diseases - Lu et, al. 2020 Mol Cell [PMID: 32592681]) in the field with > 20 loop-calling methods developed in recent years. It has also been employed in established models, such as the Activity-By-Contact (ABC) model and related studies, to systematically interpret enhancer-promoter regulatory relationships.

Furthermore, Hi-C prediction has been an active research area with demonstrated value, as reflected by multiple methods including Akita, C.Origami, Epiphany, and ChromaFold published in recent years. Hi-Compass builds upon this foundation and contributes novel capabilities particularly for single-cell and cell type specific predicting contexts. While computational methods for ChIA-PET or HiChIP prediction also have their own merits, extending to these data types would represent a different research direction beyond the scope of this work.

Comment 5: Authors have used classical structure of GANs, only the cost function is changed. I can not see where is the conceptual advancement on problem-novelty or out-of-box-solution.

Response: We thank the reviewer for this comment. As clarified in our response to Comment 2, Hi-Compass does not employ a GAN architecture. Our model uses a CNN-Transformer encoder-decoder framework, with the discriminator serving as a cell-type classifier for multi-task learning rather than adversarial training.

Regarding the contributions of Hi-Compass, our work addresses challenges at multiple levels. For bulk Hi-C prediction, Hi-Compass significantly outperforms existing methods including Akita, C.Origami, Epiphany, ChromaFold and AlphaGenome in insulation score correlation, structural similarity, and distance-stratified correlation (revised **Fig. 2f-h**). Beyond bulk data, Hi-Compass addresses a problem that has not been tackled before: predicting cell-type-specific Hi-C maps from sparse single-cell and spatial chromatin accessibility data. None of the previous methods have demonstrated the ability to handle input data spanning several orders of magnitude in sequencing depth, from high-coverage bulk profiles to sparse scATAC-seq. Our solution involves a depth-aware module that dynamically adjusts feature extraction based on sequencing depth, combined with a multi-depth training strategy and the use of a generalized CTCF profile to eliminate the requirement for cell-type-specific ChIP-seq data. These designs enable Hi-Compass to be the first method capable of predicting Hi-C from scATAC-seq and spatial ATAC-seq data, while achieved ~20% performance improvement on bulk data. Other contributions of Hi-Compass are listed in the response to Comment 1.

Comment 6: While there is lack of substantial computational method advancement, there is also no fundamental finding or biological insight from trend in the predicted chromatin interaction. The paper is suitable for journals like Briefings in bioinformatics, Bioinformatics or IEEE journals.

We thank the reviewer for this comment. We have clarified and discussed the contribution of Hi-Compass in our response to Comment 1 and 5.

Here, we'd like to emphasize that Hi-Compass's primary contribution is methodological, but it does enable discoveries that would be difficult to obtain through existing approaches. By predicting Hi-C from scATAC-seq data, we were able to identify cell-state-specific chromatin loops that distinguish closely related immune cell populations in PBMC. In embryonic heart tissue, we integrated predicted chromatin interactions with gene expression data to reveal cell-type-specific regulatory networks, finding that chromatin loop-associated genes in fibroblasts are enriched for distinct functional pathways compared to differentially expressed genes alone. We also demonstrated systematic annotation of GWAS variants to their potential target genes through predicted loops, with validation against tissue-specific eQTL data. In spatial ATAC-seq data, we revealed region-specific chromatin interactions within hippocampal tissue that correspond to spatial gene expression patterns.

Through applying Hi-Compass to diverse datasets, we have demonstrated its potential for uncovering biological insights from chromatin accessibility data. To facilitate broader adoption, we have updated our

software package with complete documentation and tutorials, providing the community with an accessible tool for exploring 3D genome organization in their own biological systems of interest.

Reviewer #2 (Remarks to the Author):

Sun, Jiang, Cai, and colleagues present Hi-Compass, a new method for predicting chromatin interactions (in the form of Hi-C maps) using DNA sequence and ATAC-seq data. Hi-Compass predictions show strong concordance with experimental Hi-C data across multiple datasets, highlighting its potential to extend chromatin studies to rare cell types where generating high-quality Hi-C maps is challenging. The manuscript is well written, logically organized, and presents an interesting approach. I have only the following suggestions that I believe could further strengthen the study:

We sincerely thank the reviewer for the positive assessment of Hi-Compass. We are grateful for the constructive suggestions, which have helped us improve the manuscript substantially. Below, we provide detailed point-by-point responses to each specific comment.

Comment 1: In Figure 1b, it would be more informative to display the same genomic region across the three cell types. This would allow readers to assess how well Hi-Compass predicts shared versus cell-type–specific chromatin interactions, and how these compare to experimental Hi-C data. Please also clarify the resolution of Hi-C maps used in Figure 1b and elsewhere in the manuscript, as well as the resolution applied in the APA analysis. Additionally, what are the resolution limits of Hi-Compass predictions?

Response: We thank the reviewer for this helpful suggestion. Following the reviewer's suggestion, we have added new **Supplementary Fig. 2** below, which displays the same three genomic regions from **Fig. 1b** across all three cell types (GM12878, K562, and IMR90). This allows readers to directly compare both shared chromatin structural features and cell-type-specific interactions at identical genomic loci.

Regarding resolution, Hi-Compass predicts Hi-C maps at 10 kb resolution, which is consistently applied across all analyses in this study including APA analysis. We have clarified this throughout the revised manuscript.

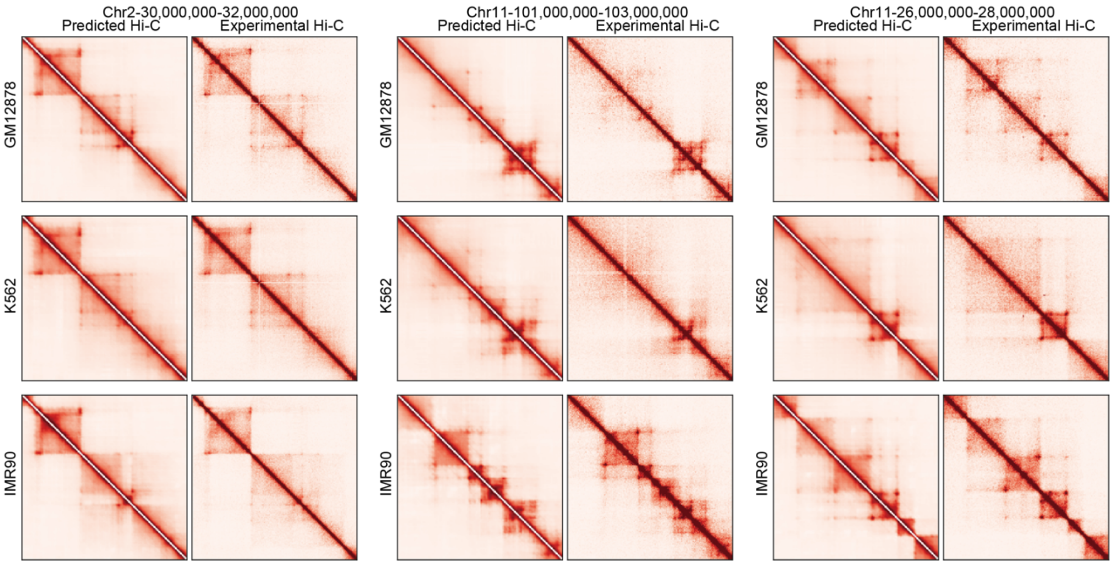

*(Legends: **Supplementary Fig. 2 | Cross-cell-type comparison of Hi-Compass predictions at identical genomic regions.** Predicted (left) and experimental (right) Hi-C contact maps for three cell lines (GM12878, K562, and IMR90) across three representative genomic regions shown in Fig. 1b. All maps are displayed at 10 kb resolution.)*

Comment 2: In addition to comparisons with Akita, C.Origami, and Epiphany, the authors should consider benchmarking against the latest AlphaGenome. Ideally, test sets should include Hi-C datasets published after AlphaGenome's release. It would also strengthen the study to include comparisons of prediction accuracy across multiple resolutions.

Response: We thank the reviewer for this suggestion. We have added comparisons with AlphaGenome as suggested, as well as ChromaFold, the most recently published Hi-C prediction method. The quantitative results are shown in revised **Fig. 2f-h** below, where Hi-Compass outperforms all compared methods in insulation score correlation, structural similarity, and distance-stratified correlation. We note that AlphaGenome can only generate predictions for a preset set of cell lines, which limits its application to new cell types. Regarding multi-resolution comparison, Hi-Compass and other existing methods including Akita, C.Origami, Epiphany, ChromaFold, and AlphaGenome all operate at fixed resolutions, making systematic comparison across multiple resolutions infeasible without retraining all models.

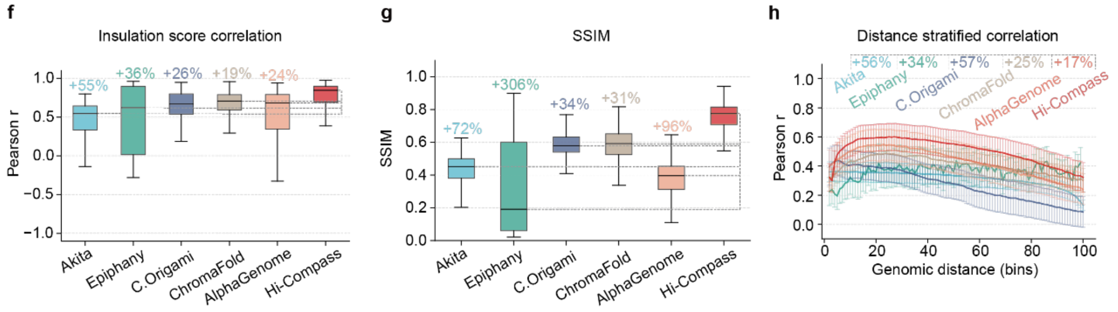

*(Legends: **Fig. 2f-h,** Performance comparison of Hi-Compass with previously published Hi-C prediction methods: Akita, Epiphany, C.Origami, ChromaFold and AlphaGenome. Box plots show the correlation of insulation scores (**f**), SSIM (**g**) and distance-stratified correlation (**h**) between predictions and experimental Hi-C data.)*

Comment 3: It is unclear whether Figures 2d and 2e represent Hi-Compass predictions or experimental Hi-C data. If they show predictions, it would be valuable to include corresponding experimental Hi-C data (especially in Fig. 2e) to demonstrate whether contrast-stretch normalization also improves visualization of real Hi-C maps.

Response: We thank the reviewer for pointing out this ambiguity. **Fig. 2d** and **Fig. 2e** display experimental Hi-C data, not Hi-Compass predictions. These figures are intended to demonstrate the effect of our contrast stretching normalization on real Hi-C matrices, showing how this preprocessing enhances the

visibility of fine-scale chromatin structures while preserving cell-type-specific differences. We have clarified this in the revised manuscript and figure legend.

Comment 4: The statement, "Further analysis revealed that most of the experimentally identified cell-type-specific loops were precisely captured by mcHi-C predictions with statistical significance (effect size > 0.6, p < 0.05)" needs clarification. Please specify the statistical test used and how effect size was defined.

Response: We thank the reviewer for this comment. We used the Mann-Whitney U test to compare predicted contact frequencies at loop peaks between the target cell type and other cell types. Effect size was calculated as the rank-biserial correlation coefficient, which ranges from -1 to 1, with values > 0.6 indicating a large effect. We have clarified this in the revised manuscript and added a detailed description in the Methods section.

Comment 5. In addition to the interesting single-cell analysis presented, I believe a broader and highly impactful application would be to predict the target genes of non-coding GWAS SNPs/mutations and identify the associated cell types. Since most GWAS signals are derived from PBMCs and may not reflect the true causal cell type, this type of analysis could greatly enhance the translational relevance of Hi-Compass. For instance, a user-friendly web application allowing researchers to upload ATAC-seq tracks and infer the most likely cell types could provide immediate, practical value to the community.

Response: We thank the reviewer for this insightful suggestion. We have updated the Hi-Compass software package and provided a detailed tutorial covering the complete workflow from (sc)ATAC-seq tracks and a list of GWAS SNPs to infer the SNP-to-gene links in the most likely cell types (https://github.com/EndeavourSyc/Hi-Compass/doc/Advanced_tutorial.md). Users can now use this workflow to study their own list of GWAS catalogs. Given the Hi-Compass prediction is typically requires substantial computational resources, we think a local analysis pipeline with comprehensive documentation is more suitable than a web application.

Comment 6: Currently, the package seems limited to prediction. To broaden utility, it would be helpful to provide a demo or workflow for training new models. Additionally, including WashU Browser or Juicebox session files for the predicted Hi-C maps would help readers and reviewers directly visualize and evaluate predictions.

Response: We thank the reviewer for these practical suggestions. We have substantially expanded the Hi-Compass software package to address these concerns. For model training, we have added complete training functionality to the package. Users can now preprocess their own paired ATAC-seq and Hi-C data through our pipelines and train models for human, mouse, or custom species. Detailed documentation and example scripts are available at https://github.com/EndeavourSyc/Hi-Compass.

For visualization, the output of Hi-Compass predictions is in standard .cool format, which can be directly

loaded into common visualization tools including HiGlass, Juicebox, and WashU Epigenome Browser.

Reviewer #2 (Remarks on code availability):

The current implementation hard-codes chromatin sizes to human, which limits applicability to widely used mouse models. In addition, only the prediction function is available, whereas the ability to train new models is equally important and should be supported. Finally, it is unclear to this reviewer why the parameter --cell_type is required—further clarification or justification would be helpful.

Response: We thank the reviewer for these comments on code availability. We have addressed all three concerns. For species support, we have updated Hi-Compass to support human (hg38), mouse (mm10), and any user-defined custom species. Users can now specify chromosome sizes for their organism of interest through a configuration file.

For training functionality, as mentioned in our response to Comment 6, we have expanded the package to include complete training capabilities with documentation and example scripts.

For the --cell_type parameter, we apologize for the confusion. This parameter was poorly named in the previous version and was only used to specify the output file name, with no effect on the prediction itself. We have renamed it to --output in the updated package to avoid ambiguity.

Reviewer #3

In this paper by Sun et al, entitled "Systematic prediction of cell-type-specific chromatin interactions reveals regulatory mechanisms across biological scales", the authors present Hi-Compass, a framework for the prediction of Hi-C maps starting from cell-specific ATAC-seq data, DNA sequence and generalized CTCF binding profile. They compare the performances of the tool to other Hi-C maps predictors and demonstrate the generalization potential to other cell types. They also demonstrate the feasibility of applying the framework to sparse chromatin accessibility data such as single cell and spatial ATAC-seq. The manuscript is well written, the methods section is accurate, and the description of the analyses is in general sound and convincing. The documentation in the github page and tutorial are sufficient. In general, the manuscript can be improved with some additional details and clarifications, I provide here some suggestions to the authors.

We sincerely thank the reviewer for the positive assessment of Hi-Compass and constructive suggestions for improving our manuscript. Below, we provide detailed point-by-point responses to each specific comment.

Comment 1: First of all, the chosen title doesn't seem appropriate and fitting to the content of the manuscript. By reading the title, one might expect a loop caller and a systematic application to identify regulatory mechanisms. However, the manuscript is more generally focused on the prediction of chromatin organization (Hi-C maps) and only provides some examples of possible chromatin loops. I suggest changing the title into one that better reflects the content.

Response: We thank the reviewer for this suggestion. We agree that the original title may have set expectations that do not fully align with the manuscript's focus. We have revised the title to:

"**Hi-Compass: a depth-aware deep learning framework for predicting cell-type-specific 3D genome organization from single-cell to spatial resolution**"

This revised title more accurately reflects that Hi-Compass is a prediction framework for chromatin organization and highlights its key capability of handling single-cell and spatial data.

Comment 2: I suggest clarifying in the main text, maybe with an additional panel in figure 1, the make up of the training/test/validation set that is, as described now, a bit confusing. Cell lines are split into training and test but also some chromosomes are split, this should be clarified. For example, in line 107 we read "To assess the generalizability of Hi-Compass, we evaluated its prediction performance on multiple unseen samples" but are these the same HiC maps generated in the previous paragraph (E.g. figure 2g with general CTCF)?

Response: We thank the reviewer for pointing out this confusion. We have clarified the

training/validation/test set design in the revised manuscript and added new **Supplementary Fig. 4a** to illustrate this.

Our data splitting strategy operates on two dimensions. For chromosomes, we designated chromosome 2 as the validation set and chromosome 11 as the test set, with all other chromosomes used for training. For cell types, we used 6 cell types for training and reserved 8 cell types exclusively for testing. This dual held-out design allows us to evaluate model performance across different combinations of seen/unseen cell types and chromosomes.

To address the reviewer's specific question: the evaluation in **Fig. 2g-i** uses this same design, where the first three cell types (GM12878, K562, IMR90) are from the training set and the last three (DND41, Gastroc, Colon) are from the test set. We have updated the figure to indicate this more clearly.

a

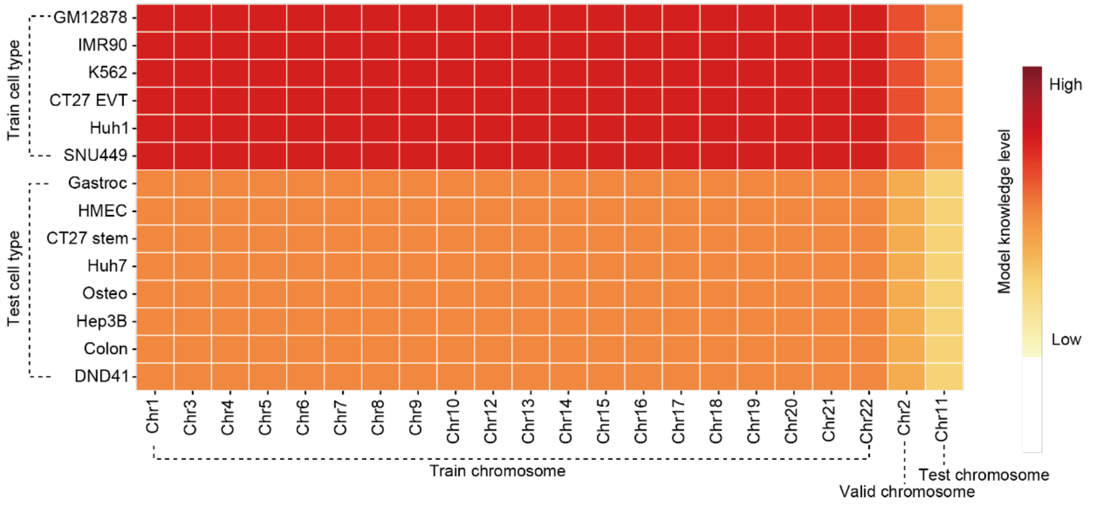

*(Legends: **Supplementary Fig. 4a**, Heatmap showing model knowledge level across cell types (rows) and chromosomes (columns). Cell types are divided into training set and test set. Chromosomes are categorized into training, validation (Chr2), and test (Chr11) sets.)*

Comment 3: The Authors report the time and computational make up used for training, but what are computational time/requirements when applying the tool for inference?

Response: We thank the reviewer for this question. We have added detailed information about inference time in the revised Methods section. Generating a genome-wide Hi-C prediction for a human sample takes approximately 8 minutes on a personal computer equipped with an NVIDIA RTX 4070Ti or a server with RTX 3090. Hi-Compass inference is not computationally demanding and can be efficiently performed on standard personal computers with consumer-grade GPUs.

Comment 4: The tutorial should contain more details on the format of the input files and on the tuning (or not) of the parameters. E.g., one of the parameters is the "depth", but how can the user define this value?

Response: We thank the reviewer for this suggestion. We have expanded our documentation to include detailed specifications for all input file formats, including ATAC-seq data (bigWig format), Hi-C data (.cool format at 10 kb resolution for training), DNA sequence (FASTA format), and CTCF profile (bigWig format, optional as we provide pre-computed generalized profiles).

The depth parameter represents the total number of reads in the input ATAC-seq data. Users can obtain this value by counting the total reads in their BAM file (e.g., using samtools view -c). We have added clear instructions for calculating and specifying this parameter in our updated documentation.

Comment 5: The software allows specifying the value for resolution but changing this value has not been discussed in the manuscript. Is it feasible to enhance resolution? Under which conditions? Should the user instead in some cases increase the bin size?

Response: We thank the reviewer for this question. Hi-Compass operates at a fixed 10 kb resolution, similar to most existing Hi-C prediction methods including C.Origami, Epiphany, and ChromaFold. The resolution parameter in the previous version of our software may have caused confusion, and we have updated the documentation to clarify this.

Comment 6: TAD and TAD boundaries are commented in the results and discussion sections but are never explicitly shown or calculated, as only the insulation score and its correlation are used. Authors could use the thresholding and call boundaries to verify that in fact there is a good overlap between predicted and experimental maps.

Response: We thank the reviewer for this suggestion. We have added TAD boundary calling and evaluation in the revised manuscript (new **Supplementary Fig. 7b**). TAD boundaries were identified from insulation score profiles using cooltools with multiple window sizes. We evaluated prediction performance using recall rate, defined as the proportion of experimentally identified TAD boundaries that were also detected in predicted Hi-C data. We chose recall rather than overall accuracy because TAD boundaries are sparse across the genome (only a small fraction of genomic bins are boundaries), making accuracy an uninformative metric (always very high). Across all tested samples, Hi-Compass achieved TAD boundary recall rates exceeding 65%. Details of the TAD boundary calling procedure are described in the Methods section.

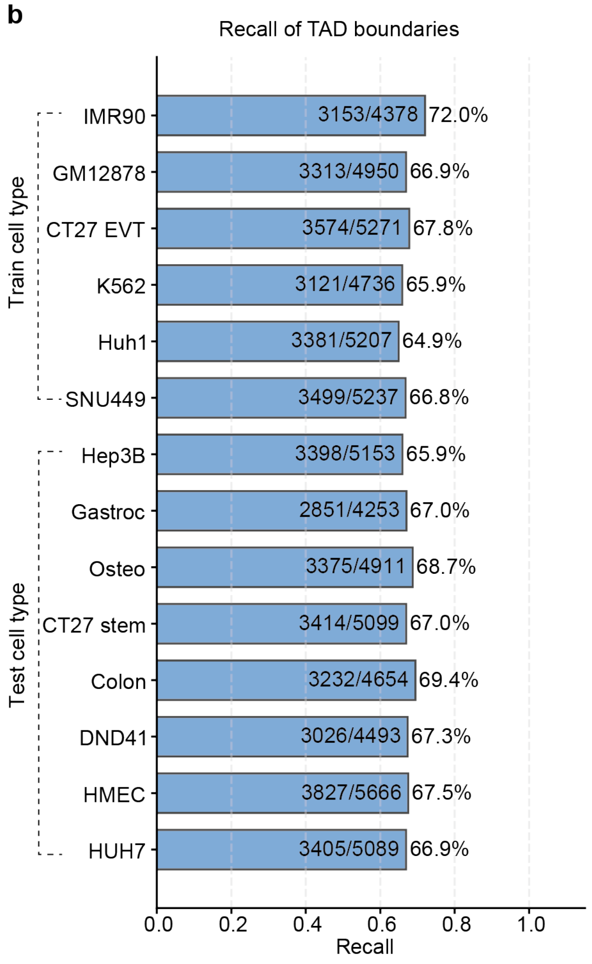

*(Legends: **Supplementary Fig. 7b**, Bar plots show the recall of TAD boundary prediction for each cell type.)*

Comment 7: Did matrices generated with the other tools have the same resolution as Hi-Compass? E.g, in the introduction it is reported that Akita generates maps at a resolution of 2kb.

Response: We thank the reviewer for this question. Different methods operate at different native resolutions: Akita and AlphaGenome at 2 kb, while C.Origami, Epiphany, ChromaFold, and Hi-Compass at 10 kb. For fair comparison, we evaluated each method at its native resolution and compared insulation scores with experimental Hi-C data processed at matching resolutions. We have clarified this in the revised Methods section.

Comment 8: Line 173: the "image preprocessing approach" is presented as one of the key aspect of Hi-Compass but it has not been previously discussed in the text. It should be mentioned when the pipeline is presented.

Response: We thank the reviewer for this observation. We have revised the manuscript to introduce the contrast stretching normalization approach earlier when describing the Hi-Compass pipeline, before it is referenced in the ablation studies.

Comment 9: Figure 2g: I suggest highlighting in figure that the last three cell lines are not used during training.

Response: We thank the reviewer for this suggestion. We have updated the relevant figures to clearly indicate which cell lines were used for training and which were held out for testing. This labeling has been added to **Fig. 2d, 2e, 2i** as well as **Supplementary Fig. 7**.

Comment 10: Figure 2i: What is the continuous signal shown at the top and bottom of this figure? Also the figure legend contains a text fragment that does not belong to this figure (line 787-790).

Response: We thank the reviewer for pointing this out. The continuous signal shown at the top and bottom of the Hi-C matrices represents insulation scores calculated with a window size of 10 bins. We have clarified this in the revised figure legend. We have also identified and removed the misplaced text fragment from the figure legend.

Comments 11-12: Line 225: the reference to figure 2c here is wrong. Other than Ext fig 3b there does not seem to be a panel related to this result.

Line 226: I find the expressions "experimentally identified loops" and "Hi-Compass-predicted loops" a bit misleading because what is meant here is "loops identified from experimental Hi-C and loops identified from predicted Hi-C". Hi-Compass does not directly predict loops.

Response: We thank the reviewer for catching these issues. We have corrected the figure reference in the revised manuscript. We have also revised the terminology throughout the manuscript to distinguish "loops identified from experimental Hi-C" from "loops identified from predicted Hi-C", to accurately reflect that Hi-Compass predicts Hi-C maps from which loops are subsequently called using standard tools.

Comments 13-15: Fig 5f: I would use a different combination of colors as red/blue is normally used for enrichment in up-and down-regulated genes.

Line 335: The reference to "developmental stages" is not clear.

Line 337: This phrase is missing a verb, please review.

Response: We thank the reviewer for these suggestions. We have revised **Fig. 5f** to use a different color scheme to avoid confusion with conventional up/down-regulation coloring. For the unclear reference, we

have revised "22 genes common across developmental stages" to "22 genes shared between fibroblast-specific loop-associated genes and DEGs" to accurately describe the intersectional analysis. We have also corrected the grammatical error in the revised manuscript.

Comments 16-18: Line 345: this enrichment analysis is not clearly discussed, what is the ratio depicted in figure 5g?

Line 380: why were these 2 genes chosen? Are these known specific markers? Moreover, they have opposite behavior in gene expression, this should be better explained in the text.

Line 386: it is not clear how the super resolution is obtained and how it is different from the meta-spot approach described previously.

Response: We thank the reviewer for these questions. For **Fig. 5g**, the enrichment ratio represents the proportion of GWAS SNPs that could be linked to target genes through predicted chromatin loops. We have clarified this in the revised manuscript.

For the gene selection in spatial analysis, we chose AGAP12P and SOX2 because they display contrasting spatial expression patterns: AGAP12P is elevated in the granular layer while SOX2 is enriched in the choroid plexus. SOX2 is a well-characterized transcription factor essential for neural stem cell maintenance. Selecting genes with opposite patterns was intentional to demonstrate that Hi-Compass can capture region-specific chromatin interactions across distinct spatial domains. We have clarified this rationale in the revised text.

For the super-resolution approach, we have clarified in the revised manuscript that while the initial meta-spot analysis divided the tissue into non-overlapping 5×5 regions, the super-resolution method applies a 5×5 sliding window with a step size of one spot, generating Hi-C predictions at the native spatial resolution of the original data.

Comment 19: Line 422: the discussion mentions "multi-omics integration capability of Hi-Compass" but it appears that Hi-Compass doesn't perform direct integration of different modalities as the RNA was used in downstream analyses to validate the quality of the differential loops.

Response: We thank the reviewer for this valid point. We have revised the **Discussion** to more accurately state "the compatibility of Hi-Compass predictions with multi-omics downstream analyses", reflecting that Hi-Compass predictions can be combined with other omics data for downstream analyses rather than directly integrating different modalities.

Reviewer #3 (Remarks on code availability): I did not try to install the code but the documentation should be improved, as reported in my comments for the authors.

Response: We have substantially improved our documentation based on the reviewer's comments, including detailed input file format specifications, parameter descriptions, and expanded tutorials covering the complete workflow from data preprocessing to downstream analyses. The updated documentation and advanced tutorial are available at our GitHub repository (https://github.com/EndeavourSyc/Hi-Compass) and (https://github.com/EndeavourSyc/Hi-Compass/blob/main/hicompass/doc/Advanced_Tutorial.md).

Dear Reviewers,

We thank the reviewers for their constructive feedback on our revised manuscript. We have carefully addressed all remaining concerns raised in this round of review. Below we provide point-by-point responses to each comment, with descriptions of the corresponding revisions. All changes in the manuscript are highlighted in red.

Reviewer #1 (Remarks to the Author):

Comment 1: They are able to recall only 65% of TAD boundaries by prediction. Given the fact that TAD boundaries are highly preserved even across cell-type and there is information leakage of genomic sequence (or loci), it is not a great result. As even if you do not run any prediction model and overlap boundary from one cell-type to other you can get 65% or more overlap. (see McArthur et al., AJHG, https://doi.org/10.1016/j.ajhg.2021.01.001) Moreover authors have not shown results about TAD boundary preservation on test chromosomes for which there was no training.

Response: Thank you for this thoughtful comment and for directing our attention to the work of McArthur et al. We noticed that McArthur et al. reported substantial variability in TAD boundary positioning across cell lines, with boundary sharing ranging from 20% to 80%—highlighting the degree of cell-type specificity.

To directly assess whether Hi-Compass's cross-cell-type prediction performance could be explained by simple boundary transfer, we conducted a systematic pairwise analysis of TAD boundary overlap across all 14 cell lines used in this study, based on experimental Hi-C data (**see figure below for reviewer reference**). For each query cell line, we computed the fraction of TAD boundaries shared with every other reference cell line. The resulting off-diagonal overlap values ranged from 11% to 50%, consistently falling below 65%. This empirical observation demonstrates that the predictive accuracy of Hi-Compass across cell types cannot be attributed to trivial transfer of boundary annotations.

In addition, following the reviewer's suggestion, we now present TAD boundary recall results separately for training chromosomes, the validation chromosome (Chr 2), and the test chromosome (Chr 11) across all 14 cell lines, including both training and test cell types (**Supplementary Fig. 8b**). Error bars indicate the standard deviation of recall across individual training chromosomes.

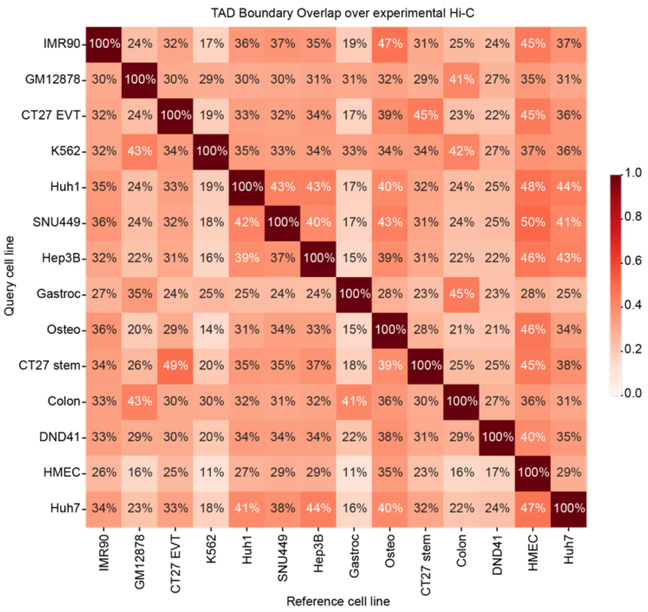

(Legends: **Pairwise TAD boundary overlap across 14 cell types from experimental Hi-C data.** Heatmap showing the fraction of TAD boundaries in each query cell line (rows) that overlap with TAD boundaries in each reference cell line (columns), computed from experimental Hi-C contact maps at 10 kb resolution.*)*

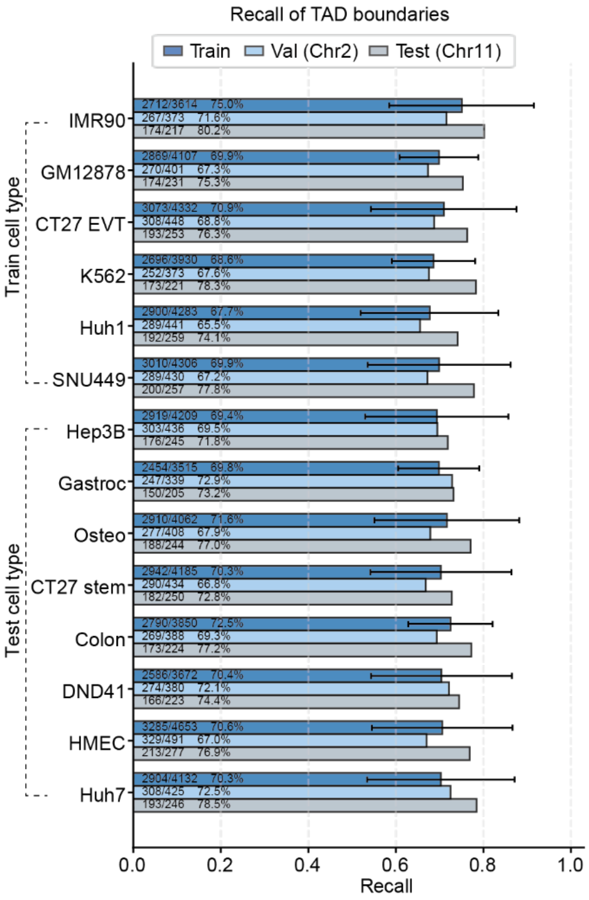

(*Legends:* ***Supplementary Fig. 8b*****,** Recall of TAD boundary prediction for each cell type, stratified by training chromosomes, validation chromosome (Chr 2), and test chromosome (Chr 11). Numbers on each bar indicate the number of recalled boundaries over total boundaries and the corresponding recall percentage. Error bars represent the standard deviation of recall across individual training chromosomes.*)*

Comment 2: Kindly try to show spearman correlation, not pearson correlation.

Response: We thank the reviewer for this suggestion. We have computed Spearman correlation coefficients for all major performance comparisons and added the results into **Supplementary Fig. 6**. The results are highly concordant with the Pearson-based evaluation presented in **Fig. 2**.

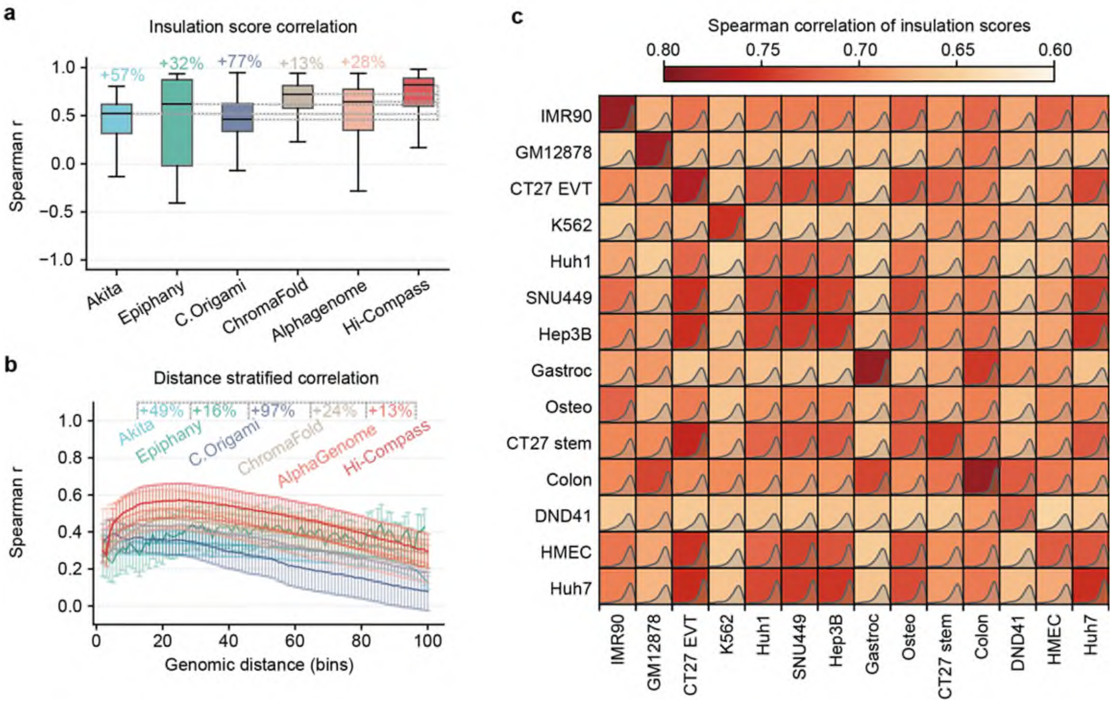

(Legends: ***Supplementary Fig. 6 | Spearman correlation analysis of Hi-Compass prediction performance. a,*** *Spearman correlation coefficients of insulation scores between predicted and experimental Hi-C across all cell types for each method.* ***b,*** *Distance-stratified Spearman correlation between predicted and experimental Hi-C contact maps as a function of genomic distance for all methods.* ***c,*** *Pairwise Spearman insulation score correlations across 14 cell types, with embedded density plots showing the distribution of per-window correlations.)*

Comment 3: In Supplementary Fig.-4, author have shown that on test data, (chromosomes 11) the prediction is better than even training data (other chromosomes), how can it be theoretically possible. May be it is at level of insulation score. However in mouse genome based prediction for test chromosome how can distance-stratified correlation be better than training set chromosomes. Can author provides some insight about it or is it just a mistake.

Response: We thank the reviewer for this observation. This is not a mistake. It's because the model was trained on a larger set of chromosomes than those used for testing. In the figure, the curves represent

mean performance averaged across chromosomes. Individual chromosomes differ in structural characteristics such as length, gene density, and compartmentalization patterns, which can affect prediction difficulty. As a result, per-chromosome performance naturally varies, and any given chromosome may fall above or below the mean. To address this concern, we provide the per-chromosome distance-stratified correlation for all chromosomes (**figure shown below for reviewer reference**). Training chromosomes (gray) exhibit substantial spread, and the test chromosomes (Chr 18 and Chr 19, red) fall within this distribution.

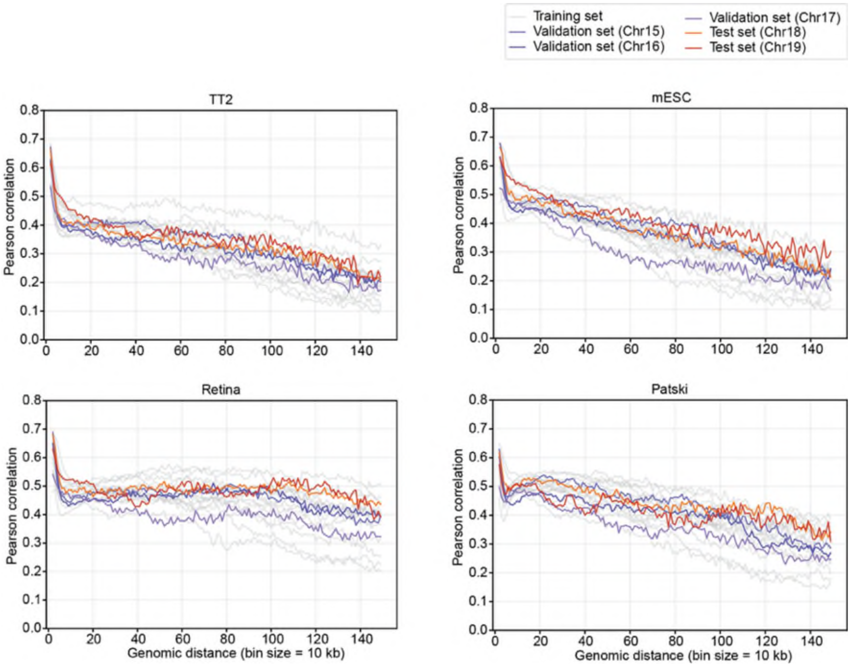

(Legends: Per-chromosome distance-stratified correlation analysis of Hi-Compass predictions across mouse cell types. Line plots display Pearson correlation coefficients across genomic separation distances for individual training set chromosomes (gray), validation chromosomes (Chr15–17), and test chromosomes (Chr18–19).)

Comment 4: One comment completely ignored: Authors are using high-resolution ATAC-seq profile and they claim high resolution interaction prediction, then, why they could not produce result to my comment on overlapping with ChiA-pet and Hi-ChIP interaction. Authors should also benchmark on interaction from ChiA-pet or Hi-ChIP.

Response: We thank the reviewer for the further clarification of this point. We have now benchmarked Hi-Compass predictions against two independent cohesin-mediated interaction datasets in GM12878.

Specifically, we performed Aggregate Peak Analysis (APA) using cohesin HiChIP loop calls from Mumbach et al. (Nature Methods, 2016) (n = 5,337) and RAD21 ChIA-PET loops from Grubert et al. (Nature, 2020) (n = 5,983). Both experimental and Hi-Compass-predicted Hi-C contact maps showed clear focal enrichment at the positions of these loops (**Supplementary Fig. 10**). The consistent enrichment observed across these two orthogonal technologies demonstrates that Hi-Compass predictions reliably capture cohesin-mediated chromatin interactions.

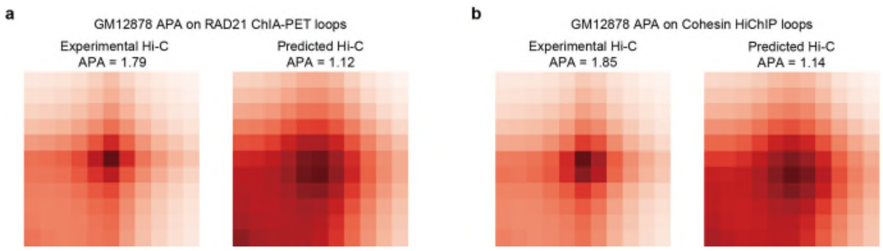

*(Legends: **Supplementary Fig. 10 | Validation of Hi-Compass predictions against orthogonal protein-mediated chromatin interaction data.** Aggregate Peak Analysis was performed by mapping RAD21 ChIA-PET loop positions (**a**, n = 5,983) and cohesin HiChIP loop positions (**b**, n = 5,337) onto experimental and Hi-Compass predicted Hi-C contact maps in GM12878. APA scores are indicated above each heatmap.)*

Reviewer #3 (Remarks to the Author):

I would like to thank the authors for their thorough responses to my previous comments. I am satisfied with the majority of the revisions made; however, I have one remaining concern and a couple minor observations.

Response: We thank the reviewer for the thorough evaluation and are pleased that the majority of revisions were found satisfactory. We have addressed the remaining points below.

Comment1: In the application of Hi-Compass on spatial data, the text reports "AGAP12P, which showed elevated expression in the granular layer (Fig. 6e), and SOX2, a well-characterized transcription factor essential for neural stem cell maintenance and neurogenesis that was enriched in the choroid plexus" but all figures seem to point out that AGAP12P is expressed both in granular layer and choroid plexus (Fig 6g and clusters R4 and R6 in the UMAP of figure 6i) whereas SOX2 is not expressed in such tissues and expressed in all other areas (Figure 6h and 6j). Also interactions in Figure 6k and 6l and Supplementary Fig. 18c 18d support this.

Moreover, the panels of figure 6 are mis-aligned with the description in the figure legend and are also not correctly referenced with the text in the results section.

Response: We thank the reviewer for the careful examination. We have now corrected the spatial expression descriptions for AGAP12P and SOX2 to accurately reflect the data presented. Additionally, we have revised the corresponding figure legend and figure reference in the text.

Comment 2: Finally, I appreciate that the authors included a scheme about the training and test. When not explicitly stated, should it be assumed that the shown results refer to the test chromosomes? E.g. in Supplementary Fig. 7a are correlation scores relative to only the test chromosome?

Response: We thank the reviewer for raising this point. The insulation score correlations presented in previous Supplementary Fig. 7a are computed across all chromosomes by default, representing overall prediction quality for each cell type. We have added this clarification to the figure legend, and have also

added **Supplementary Fig. 9**, which separately displays insulation score correlation heatmaps for the validation chromosome (Chr 2) and the test chromosome (Chr 11) across all cell type comparisons. The results confirm that prediction quality on held-out chromosomes is comparable to the genome-wide evaluation, providing further evidence that Hi-Compass generalizes robustly beyond its training data.

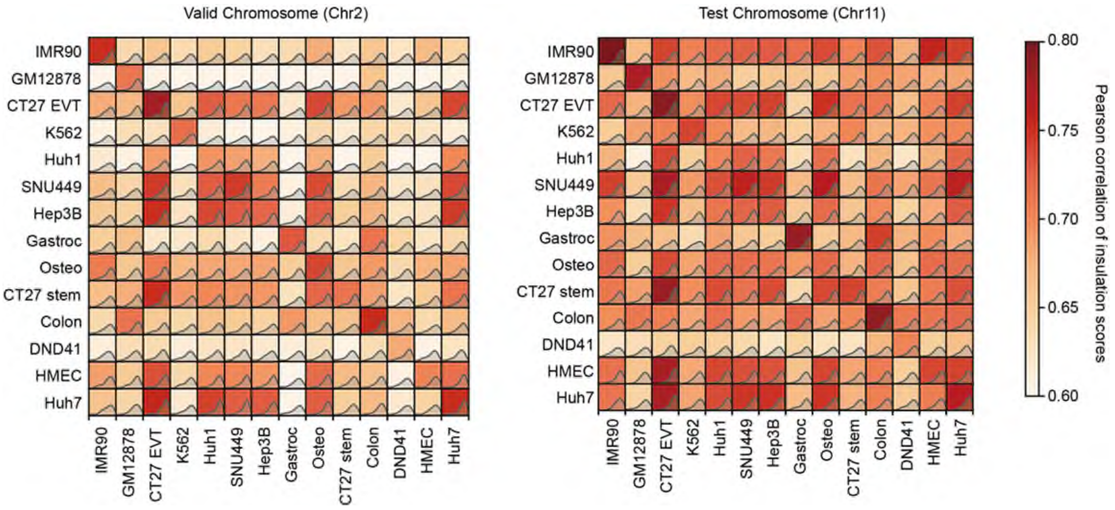

*(Legends: **Supplementary Fig 9.** Insulation score correlation between Hi-Compass-predicted and experimental Hi-C on held-out chromosomes. Heatmaps display Pearson correlation coefficients of insulation scores across all cell types for the validation chromosome (Chr 2) and the test chromosome (Chr 11).)*
